# Exploring the diversity and disparity of rhabdodontomorph ornithopods from the Late Cretaceous European archipelago

**DOI:** 10.1038/s41598-025-98083-z

**Published:** 2025-04-30

**Authors:** Łukasz Czepiński, Daniel Madzia

**Affiliations:** 1 Institute of Paleobiology, Polish Academy of Sciences, Twarda 51/55, Warsaw, 00-818 Poland; 2 Institute of Evolutionary Biology, Biological and Chemical Research Centre, Faculty of Biology, University of Warsaw, ul. Żwirki i Wigury 101, Warsaw, 02-089 Poland

**Keywords:** Palaeontology, Taxonomy, Herpetology

## Abstract

The origin and early diversification of ornithopods, a major clade of ornithischian dinosaurs, remain poorly understood, with conflicting phylogenetic hypotheses regarding rootward neornithischian relationships. Some topological stability is inferred near the basal divergence of Iguanodontia, though the ingroup relationships remain unclear. For instance, Rhabdodontidae, a clade of Late Cretaceous European ornithopods ‘traditionally’ considered to include eight to nine species, presents significant taxonomic challenges. We explore the diversity and disparity of European *Rhabdodon*-lineage iguanodontians. We assembled a novel dataset comprising morphological and morphometric data obtained from rhabdodontomorph dentaries, which are abundant, well-preserved in the majority of the taxa, and distinctive. Special attention is given to *Rhabdodon septimanicus*, a poorly known taxon from the upper Campanian to lower Maastrichtian of southern France, established based on a particularly robust dentary bone that has been subjected to conflicting taxonomic interpretations. Our restudy of the specimen, combined with a multivariate and phylogenetic assessment, shows that this taxon is a clear morphological outlier among European rhabdodontomorphs, providing a basis for its assignment to a new genus, *Obelignathus*. Although further large-scale studies, especially detailed osteological descriptions, are needed to clarify the taxonomic significance of certain European rhabdodontomorphs, our results indicate that the group exhibits greater diversity than currently recognized, with several sympatric taxa co-occurring, at least in southern France and possibly also in Romania.

**Supplementary Information:**

The online version contains supplementary material available at 10.1038/s41598-025-98083-z.

## Introduction

Untangling the complexity surrounding the origin and earliest diversification of ornithopods—a large evolutionary branch of dinosaurs that existed for more than 100 million years and encompassed a wide array of lineages, including some of the most dominant high-fiber herbivores among Mesozoic tetrapods—has proven to be a particularly difficult task. There has been a surge of interest in solving the puzzles surrounding the rootward relationships among Neornithischia, the more inclusive clade that consists of ornithopods, ceratopsians, pachycephalosaurs, and numerous related forms and lineages some of which had long been associated with the base of Ornithopoda^1–3^, but the results have been inconclusive, offering strikingly different phylogenetic hypotheses^4–12^.

Within Ornithopoda, tree structures appear relatively stable around the node marking the basal divergence of Iguanodontia, a species-rich clade of ornithopods that includes dryosaurids, ankylopollexians, and the *Rhabdodon*-lineage ornithopods, long known from a low-diversity group of latest Cretaceous taxa comprised within Rhabdodontidae^13–16^.

**Fig. 1:**
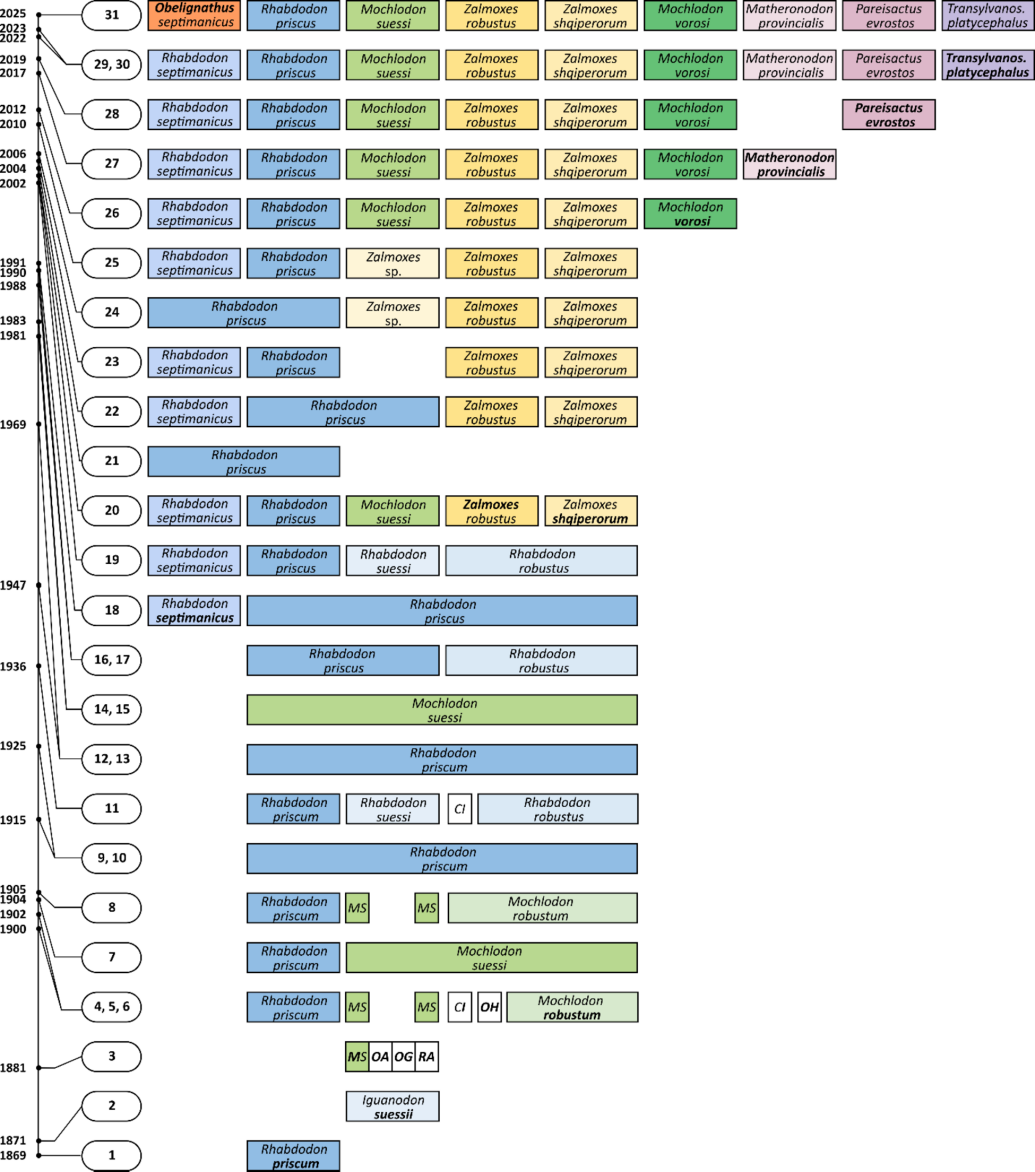
Taxonomic history of the currently existing nominal taxa of the European rhabdodontomorph dinosaurs. Individual species are shown in a different shade of color for the particular genus (blue for *Rhabdodon*, green for *Mochlodon*, orange for *Zalmoxes*, red for *Obelignathus* gen. nov.; other valid taxa are in purple and violet, while the invalid taxa are in white). The first use of the name is shown in bold font. Empty spaces indicate that the taxon was not mentioned in the given paper. Note that originally *Rhabdodon priscus* was spelled *R. priscum*^18,25^, and *Mocholodon suessi* was *‘Iguanodon’ suessii*^17^. References: 1^18^; 2^17^; 3^26^; 4^27^; 5^28^; 6^29^; 7^30^; 8^31^; 9^32^; 10^33^; 11^34^; 12^35^; 13^36^; 14^37^; 15^38^; 16^39^; 17^40^; 18^19^; 19^41^; 20^13^; 21^22^; 22^42^; 23^43^; 24^44^; 25^23^; 26^14^; 27^20^; 28^21^; 29^16^; 30^15^; 31: this study. Abbreviations: CI: *Camptosaurus inkeyi*; MS: *Mochlodon suessi*; OA: *Oligosaurus adelus*; OG: *Ornithomerus gracilis*; OH: *Onychosaurus hungaricus*; RA: *Rhadinosaurus alcimus* (partial); Transylvanos.: *Transylvanosaurus*.

**Fig. 2:**
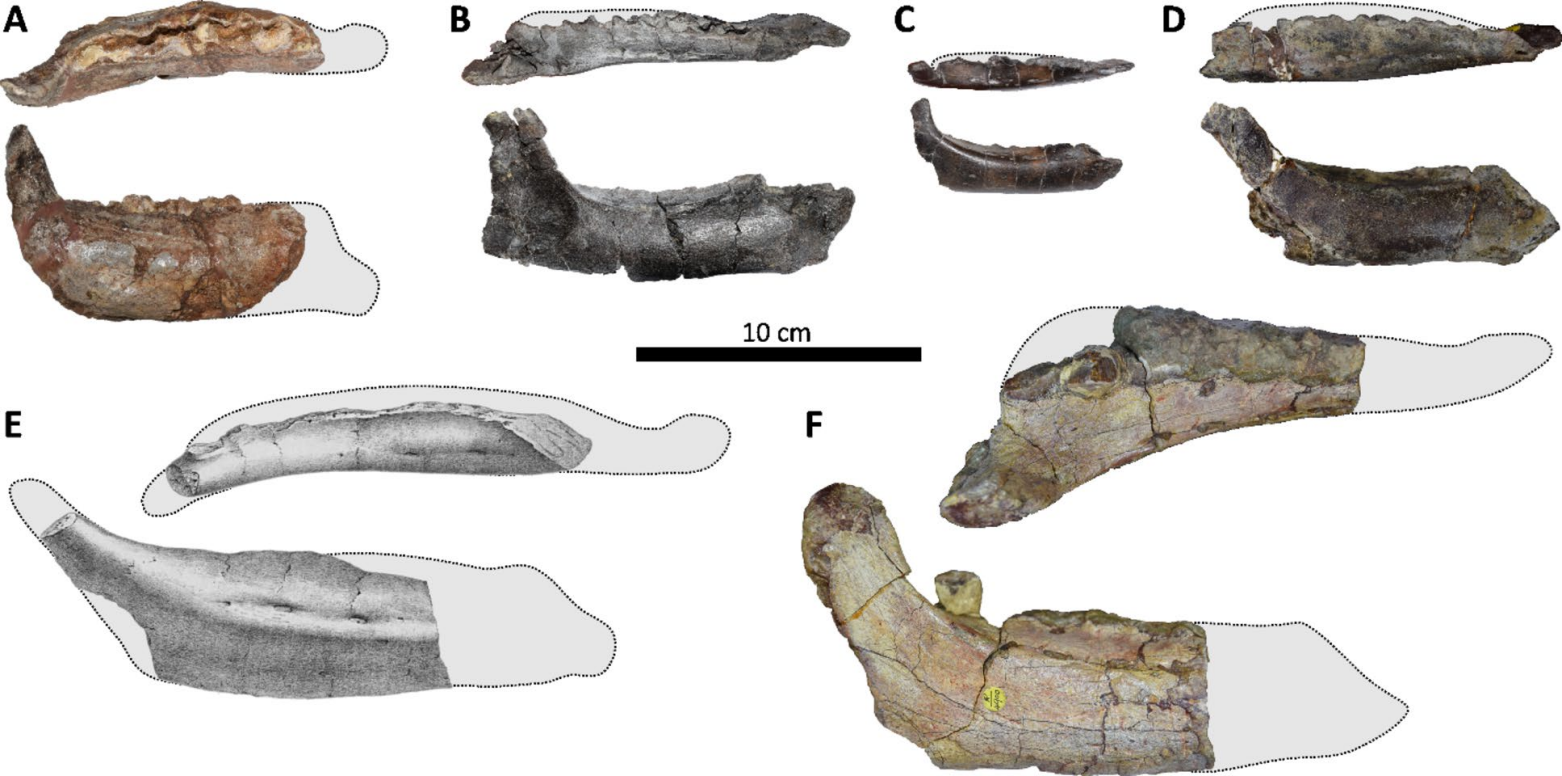
Dentaries of selected European rhabdodontomorphs in dorsal and lateral views. (A) *Obelignathus septimanicus* gen. et comb. nov., holotype MDE D30, right dentary from the ‘Grès à Reptiles’ Formation, Montouliers (France); (B) *Mochlodon vorosi* holotype MTM V 2010.105.1, left dentary from the Csehbánya Formation, Iharkút (Hungary; mirrored); (C) *Mochlodon suessi*, lectotype PIUW 2349/2, right dentary from the Grünbach Formation, Muthmannsdorf (Austria); (D) *Zalmoxes robustus* holotype NHMUK R3392, right dentary from the Sinpetru Formation, Sânpetru (Romania); (E) *Rhabdodon priscus*, lectotype MPLM 30, left dentary from the Marnes Rouges Inférieures Formation, la Nerthe (France; mirrored drawing after Matheron^18^; outline based on specimens MPLM 30 and MPLM 31, size estimation based on Matheron^18^ and Brinkman^39^); (F) *Zalmoxes shqiperorum* holotype NHMUK R4900, right dentary from the Sinpetru Formation of Sânpetru (Romania; outline based on the specimen UBB NVZ1-1).

Rhabdodontids are commonly considered to represent a group of small to medium-sized ornithischian dinosaurs endemic for the Late Cretaceous European archipelago (see Augustin et al.^15^ for a recent review of the evolutionary history of the group). Eight to nine distinct species are currently recognized based on specimens unearthed from eastern Austria^17^, southern France^18–20^, western Hungary^14^, western Romania^13,16^, and northern Spain^21^ (Figs. 1 and 2). Still, the understanding of the diversity of Rhabdodontidae and their intrarelationships remains far from complete. For instance, recent studies focusing on the histology of long bones suggested that the taxic diversity within the clade may be greater than traditionally thought, with possible co-occurrences of multiple sympatric taxa at least in the sample from southern France^14^. The group is therefore in need of a detailed taxonomic reassessment.

Owing to the fact that the type specimens of six of the taxa ‘traditionally’ comprised within Rhabdodontidae (*Mochlodon suessi*, *M. vorosi*, *Rhabdodon priscus*, *R. septimanicus*, *Zalmoxes robustus*, and *Z. shqiperorum*), are known from well-preserved and distinctive dentary bones, determining the diagnostic features present in these elements has the potential to provide crucial information improving the knowledge of their distinguishability and phylogenetic affinities. The main aim of this study is threefold: (1) to provide a thorough reevaluation of the dentary bone morphologies of these taxa, (2) to investigate the taxonomic significance of the new observations, and (3) to explore the phylogenetic implications of the obtained results on the European *Rhabdodon*-lineage iguanodontians.

A major part of the taxonomic section of the present study is dedicated to a thorough reevaluation of the poorly known ornithopod *Rhabdodon septimanicus* Buffetaut & Le Loeuff, 1991, from the upper Campanian-lower Maastrichtian ‘Grès à Reptiles’ Formation in Hérault, southern France. The taxon was established upon a robustly-built right dentary (MDE D30) missing the anteriormost portion. Although originally considered distinct from the type species, *R. priscus*^19^, further studies have provided conflicting taxonomic interpretations. Whereas Allain and Pereda Suberbiola^22^ regarded the differences between *R. priscus* and *R. septimanicus* as merely reflecting infraspecific variability or sexual dimorphism, later studies were in favor of the distinctiveness of the two taxa (Fig. 1)^13,14,23^. However, none of the subsequent studies have assessed *R. septimanicus* specifically.

We provide a detailed description of the type dentary of *R. septimanicus*, identify new diagnostic characters, compare it with the dentaries of all other European rhabdodontomorphs (the largest clade containing *Rhabdodon priscus* Matheron, 1869 but not *Hypsilophodon foxii* Huxley, 1869 and *Iguanodon bernissartensis* Boulenger in Beneden, 1881; see:^24^) in which they are preserved, illustrate it using photogrammetric three-dimensional (3D) modeling, discuss the taxonomic significance of our new findings, and explore the phylogenetic affinities of the taxon.

## Material and methods

### Material

The present study is primarily based on first-hand examination of 31 rhabdodontomorph dentaries unearthed from Upper Cretaceous strata across Europe, including those of ‘*Rhabdodon*’ *septimanicus* (holotype: MDE D30), *Mochlodon suessi* (lectotype: PIUW 2349/2), *Mochlodon vorosi* (holotype: MTM V 2010.105.1; and referred dentaries: MTM V 2010.103.1, MTM V 2010.104.1, MTM V 2010 106.1, MTM V 2010 107.1, MTM V 2010 108.1, MTM V 2010 109.1, MTM V 2010.112.1, MTM PAL 2012.15.1, MTM PAL 2012.16.1), *Zalmoxes robustus* (holotype: NHMUK R3392; and referred dentaries: NHMUK R3406, NHMUK R3407, NHMUK R4912, LPB (FGGUB) R.0088, LPB (FGGUB) R.1258, LPB (FGGUB) R.1372, LPB (FGGUB) R.1523, LPB (FGGUB) R.2369, MCDRD 310), *Zalmoxes shqiperorum* (holotype: NHMUK R4900; and referred dentaries LPB (FGGUB) R.2349, LPB (FGGUB) R.2411, UBB NVZ1-1, UBB NVZ1-2), as well as several other rhabdodontid specimens from Romania (LPB (FGGUB) R.2776, LPB (FGGUB) R.2777, LPB (FGGUB) R.2778, SZTFH Ob.3065).

 The lectotype and paralectotype dentary of *Rhabdodon priscus* (MPLM 30 and MPLM 31) could not be examined in person because they were not re-located in the MPLM collection during the visit of one of us (Ł.C.) to Marseille in November 2023. Moreover, the lectotype specimen was reported to be highly deteriorated^41^. Hence, for the comparative studies, we used the drawings from Matheron^18^ and archival photography from Brinkman^39^.

### Photogrammetry

 The photogrammetric three-dimensional (3D) model was created using photographs of the specimen MDE D30 in the Agisoft Metashape Professional 2.0.1 software. The obtained model was later processed in Meshlab 2021.10^45^, using the Cook-Torrance and Radiance Scaling shaders. The mesh of the specimen MDE D30 is available at MorphoSource (https://www.morphosource.org/concern/media/000697351).

### Multivariate analyses

We explored the disparity in the dentary bones of rhabdodontomorph ornithopods through the assessment of a newly assembled specimen-level dataset comprising 15 continuous and eight discrete characters scored for 47 elements, 45 of which have been unearthed across Europe and two are from North America. The character list is provided in Supplementary Information I, the dataset is in Supplementary Information II, and software-executable scripts are included as Supplementary Information III and IV.

We performed a principal coordinates analysis (PCoA), using the full matrix and the Gower similarity index that is well suited for datasets including both continuous and discrete variables^46^, and a principal component analysis (PCA), using only the continuous variables. In both analyses, all continuous variables were z-transformed. The analyses were conducted in PAST 4.17^47^. Before each analysis, we imposed a 50% completeness threshold to exclude highly incomplete specimens, as their extensive missing data could hinder accurate estimation of pairwise distances. The PCoA utilized data from 34 specimens, whereas the PCA assessed 33 specimens.

### Phylogenetic analyses

The phylogenetic relationships among rhabdodontomorph ornithopods were investigated using a modified version of the matrix assembled by Fonseca et al.^12^, to which we have made the following changes. ‘*Rhabdodon*’ *septimanicus* has been rescored based on personal observations and constructed to include only data obtained from the type specimen (MDE D30). ‘*R.*’ *septimanicus* has typically been assumed to be conspecific with or the sister taxon to *R. priscus*^15,19^, but the material has never been included in a phylogenetic analysis until the study by Fonseca et al.^12^, who reconstructed it as a non-rhabdodontid rhabdodontoid; thus, at an earlier-diverging position than *R. priscus*. However, in their analyses, Fonseca et al.^12^ also included characters present in material from Quarante that has only tentatively been referred to ‘*R.*’ *septimanicus* in a formally unpublished doctoral thesis^23^. Owing to the fact that this material awaits formal description, we decided to keep only those scores that can be obtained from the type specimen (MDE D30), and scored the Quarante material as a separate operational taxonomic unit (OTU), termed here as the ‘Quarante rhabdodontomorph’. We have further reassessed the scores provided for *Rhabdodon priscus* and included two OTUs: one comprising only the scores that can be inferred from the type (lectotype) and paralectotype dentaries ascribed to the taxon by Matheron^18^, and one based on referred material. Additionally, we have rescored a number of character states for the late-diverging rhabdodontoids *Mochlodon suessi*, *Mochlodon vorosi*, *Zalmoxes robustus*, and *Zalmoxes shqiperorum*, as well as for *Convolosaurus marri*, *Iani smithi*, *Leptoceratops gracilis*, *Protoceratops andrewsi*, *Tenontosaurus dossi*, and *Tenontosaurus tilletti*. Finally, we have included the recently described ornithopods *Ampelognathus cogeni*^48^ and *Emiliasaura alessandrii*^49^. *A. coheni* was proposed to represent a rhabdodontomorph^12^ though no phylogenetic analyses have supported such a placement so far. In turn, *E. alessandrii* is of potential interest for inferences concerning the evolutionary history of Rhabdodontomorpha as it was reconstructed to represent the earliest-diverging member of the clade^49^. The final version of the matrix comprised 176 OTUs scored for 943 characters (+ one dummy character); 55 of these were set as ‘additive’ (= ‘ordered’), as originally proposed by Fonseca et al.^12^.

Our analyses used maximum parsimony as the optimality criterion and were performed in TNT 1.6^50^. We have conducted four analyses, all using *Euparkeria capensis* as the outgroup; the first analysis was based on equal weights, whereas the remaining runs used the implied weighting function. The concavity parameters (*K*) were set 12, 15, and 21. Each of the analyses used the same software settings. Owing to the addition of the command “hold 200000;” to the TNT-executable script, the maximum number of most parsimonious trees was automatically set to 200,000 trees upon opening the .tnt file. First, we ran the ‘New Technology’ search, involving 500 addition sequences. We used default settings activated for sectorial searches, ratchet, drift, and tree fusing. After obtaining the initial results, we conducted an additional ‘Traditional’ search using tree bisection-reconnection (TBR) branch-swapping, with trees stored in RAM. For the analysis with equal weighting, Bremer support was calculated using TBR branch-swapping, retaining sub-optimal trees with up to three additional steps. Nodal support in the analyses with implied weighting was assessed through Symmetric Resampling, utilizing a ‘Traditional’ search with 1,000 replicates, a default change probability of 33%, and results expressed as frequency differences (GC). See Supplementary Information V for detailed information regarding the changes applied to the character scores and Supplementary Information VI for the TNT-executable script.

### Nomenclatural acts

This published work and the nomenclatural acts it contains have been registered in ZooBank, the online registration system for the International Code of Zoological Nomenclature (ICZN). The ZooBank LSIDs (Life Science Identifiers) can be resolved and the associated information viewed through any standard web browser by appending the LSIDs to the prefix https://zoobank.org/. The LSIDs are urn:lsid:zoobank.org:pub:40815043-9BE6-4770-A91A-2425EDE032A4 for this publication and urn:lsid:zoobank.org:act:3AB06061-8DE6-4E63-9139-D587A0E30CD9 for the new genus *Obelignathus*.

### Institutional abbreviations

**CM**, Collection Méchin, Vitrolles, France; **LPB** (**FGGUB**), Laboratory of Paleontology, Faculty of Geology and Geophysics, University of Bucharest, Bucharest, Romania; **MC**, Musée de Cruzy, Cruzy, France; **MCDRD**, Museum of Dacian and Roman Civilisation, Deva, Romania; **MDE**, Musée des Dinosaures, Espéraza, France; **MHN-AIX-PV**, Natural History Museum, Aix-en-Provence, France; **MHNH**, National Museum of the Natural History, Paris, France; **MPLM**, Palais Longchamp Museum, Marseille, France; **MTM**, Hungarian Natural History Museum, Budapest, Hungary; **NHMUK**, Natural History Museum, London, UK; **PIUW**, Department of Palaeontology, University of Vienna, Vienna, Austria; **SZTFH**, Collection of the Supervisory Authority for Regulatory Affairs, Budapest, Hungary; **UBB**, Faculty of Biology and Geology, Babeș-Bolyai University, Cluj-Napoca, Romania.

## Results

For the sake of convenience, we refer to ‘*Rhabdodon*’ *septimanicus* by its new genus name, *Obelignathus*, in our description and visualization of the results. For details on the nomenclatural acts, see ‘Material and methods’ above and the ‘Systematic paleontology’ section below.

### Multivariate analyses

**Fig. 3:**
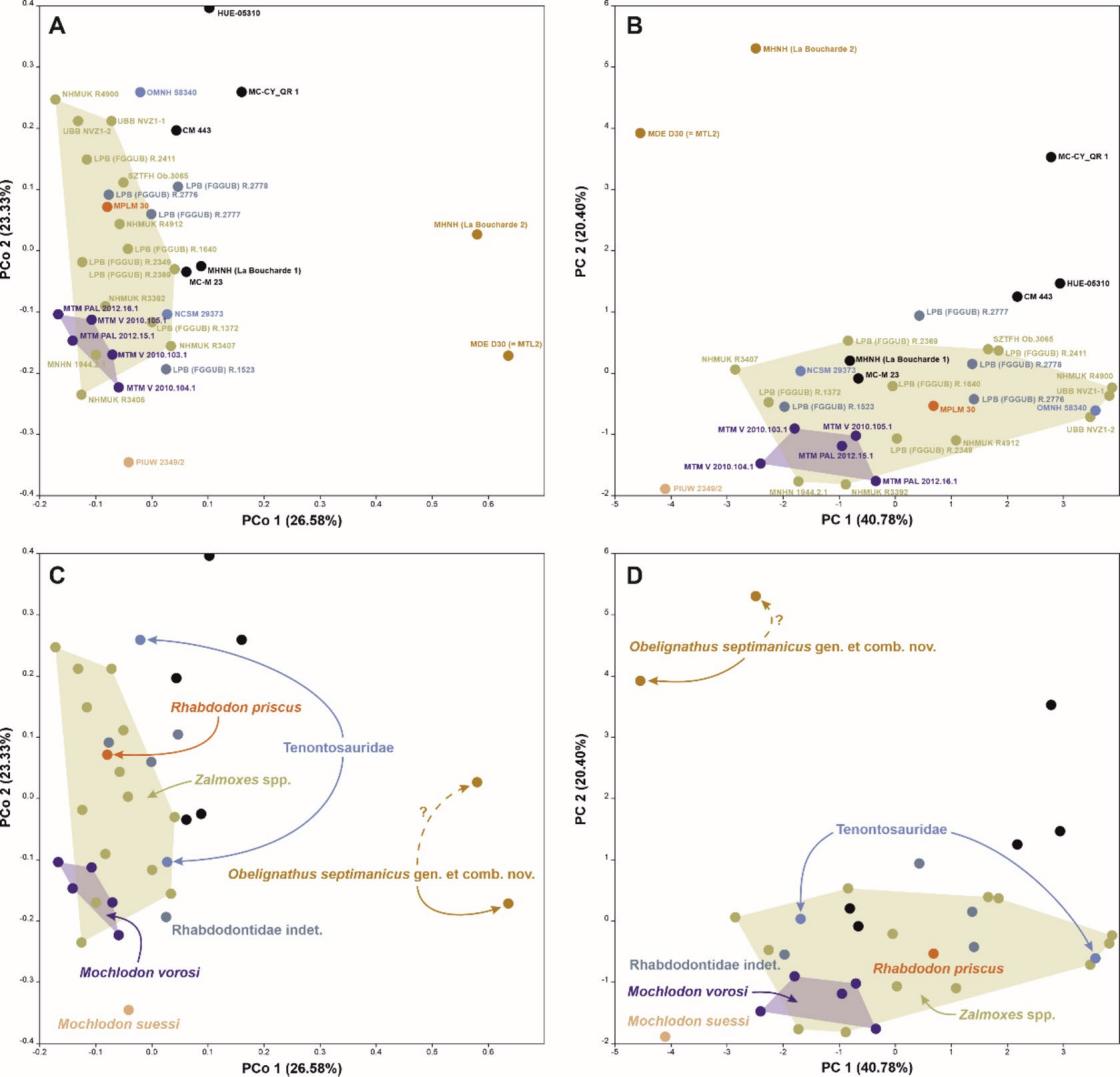
Results of the principal coordinates (**A**, **C**) and principal component (**B**, **D**) analyses showing the morphospace occupation of rhabdodontomorph ornithopods determined through assessment of the morphology and morphometrics of their dentaries.

Our multivariate analyses reveal a distinct separation in the morphospace occupied by *Obelignathus septimanicus* compared to other rhabdodontomorphs, as evidenced by the first two coordinates (PCo1 vs. PCo2) and the first two components (PC1 vs. PC2), establishing it as a clear outlier within the analyzed dataset in both its morphology as well as its morphometrics (Fig. 3). In our PCoA, *O*. *septimanicus* occupies an extreme positive side for the first coordinate, while the majority of rhabdodontomorphs, including the tenontosaurids *Tenontosaurus tilletti* and *Iani smithi*, are grouped near the center of the plot, leaning towards the negative side of coordinate 1. The results of the PCA show a similar pattern. *O*. *septimanicus* is placed on the extreme positive side of the second component, while the rest of the specimens occupy the center of the plot. Owing to the fact that *Zalmoxes* is in need of a thorough reevaluation^51^, all dentaries referred to as *Z. robustus*, *Z. shqiperorum* or *Z*. sp. have been assigned here to a single group (‘*Zalmoxes* spp.’). In both analyses, *Zalmoxes* occupies a broad morphospace that partly overlaps with that of *Mochlodon vorosi* and tenontosaurids, and covers the morphospace occupied by the data point representing the lectotype dentary of *R. priscus*. Interestingly, both analyses also show an apparent separation in the morphospace occupation of *Mochlodon suessi* and the rest of the later-diverging rhabdodontoids included in the dataset, supporting the distinctiveness of the type specimen of that taxon. The full results of our multivariate analyses are provided alongside the dataset in Supplementary Information II.

### Phylogenetic analyses

**Fig. 4:**
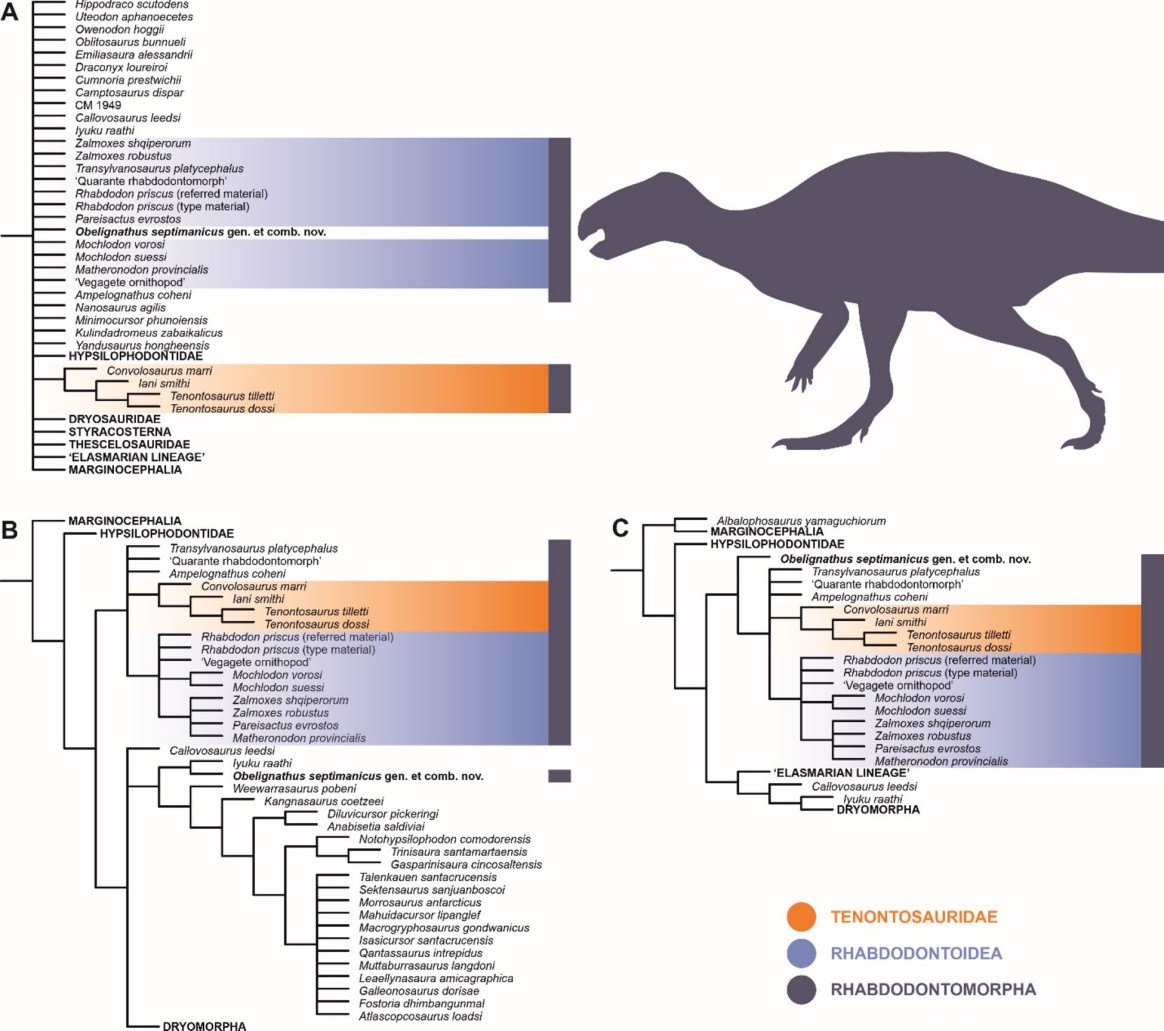
Results of the phylogenetic analyses showing reduced topologies obtained through parsimony analyses using (**A**) equal weights and the implied weighting with *K*-values of (**B**) 12 and (**C**) 15 and 21. Although the results are inconsistent in reconstructing the intrarelationships of rhabdodontomorphs and rhabdodontoids, the topological uncertainties likely arise from incomplete knowledge of the morphology of some rhabdodontomorphs. The indicated extents of particular clade names reflect the hypothesized composition of the clades. Silhouette obtained from phylopic.org (Scott Hartman, CC BY 3.0).

The numerical results of our phylogenetic analyses, including the numbers of most parsimonious trees (MPTs), tree lengths, and Consistency and Retention indices are provided in Table 1; reduced tree topologies, focusing on the rhabdodontomorph segments of the ornithischian phylogenetic trees, are visualized on Fig. 4, and full tree topologies resulting from each run of our phylogenetic analyses, including the nodal support values, are accessible through Supplementary Information VII (Figs S7.1–S7.8). The parsimony analysis using equal weights has resulted in a rather poorly resolved neornithischian segment of the strict consensus tree and failed to reconstruct a monophyletic Rhabdodontomorpha (as inferred by Fonseca et al.^12^; Fig. 4A). Further inspection of the most parsimonious trees (MPTs) and the reconstruction of the 50% majority-rule consensus tree did not improve the resolution substantially though the North American branch, Tenontosauridae (including *Convolosaurus marri*, *Iani smithi*, and *Tenontosaurus* spp.), was inferred monophyletic in all runs. Neither Rhabdodontoidea nor Rhabdodontidae were reconstructed though 99% of the MPTs inferred monophyletic *Mochlodon* spp. and 84% of the shortest trees found an unresolved clade formed by *Zalmoxes robustus*, *Z. shqiperorum*, and *Matheronodon provincialis*.

The tree resolution was higher when using the implied weighting function (Fig. 4B, C). All runs found Rhabdodontomorpha composed of a basal polytomy comprising *Transylvanosaurus platycephalus*, the ‘Quarante rhabdodontomorph’, *Ampelognathus coheni*, and monophyletic tenontosaurids and rhabdodontoids, with the same internal topologies. The only difference in rhabdodontomorph intrarelationships was the placement of *Obelignathus septimanicus* under *K* = 12—a parameter setting that, among the *K*-values tested in this study, applied the strongest downweighting of homoplastic characters. When the *K*-value was set to 12, *O. septimanicus* was excluded from the rhabdodontomorphs and instead nested within the elasmarian lineage. It was positioned as a member of the earliest-diverging clade within this group, which consisted of *O. septimanicus* and *Iyuku raathi* from the Valanginian (Lower Cretaceous) of South Africa^52^.

**Table 1:** Numerical results of the phylogenetic analyses. BS, best score (tree length); CI, Consistency Index; EW, parsimony analysis using equal weighting; IW, parsimony analysis using implied weighting; MPT, number of most parsimonious trees; NT, ‘New Technology’ search; RI, Retention Index; TS, ‘Traditional’ search.

Run	MPT (NT)	BS	MPT (TS)	CI	RI
**EW**	24	7028	200,000	0.162	0.627
**IW (*K*** **= 12)**	19	282.82054	200,000	0.162	0.626
**IW (*K*** **= 15)**	5	245.66792	200,000	0.162	0.626
**IW (*K*** **= 21)**	6	195.13297	200,000	0.162	0.626

### Systematic paleontology

Dinosauria Owen, 1842 [Langer et al., 2020].

Ornithischia Seeley, 1888 [Madzia et al., 2021].

Ornithopoda Marsh, 1881 [Madzia et al., 2021].

Iguanodontia Baur, 1891 [Madzia et al., 2021].

Rhabdodontomorpha Dieudonné et al., 2016 [Madzia et al., 2021].

*Obelignathus* gen. nov.

#### Etymology

A combination of Obélix, the name of a cartoon character in the French comic book series *Asterix*, or *Asterix and Obelix*, by René Goscinny and Albert Uderzo, known for his exceptional strength, as a reference to the unusually robustly-built holotype dentary; and “*gnáthos*” (“γνάθος”), Greek for “jaw”.

#### Diagnosis

As for the type and only species (see below).

*Obelignathus septimanicus* (Buffetaut & Le Loeuff, 1991) comb. nov.

*= Rhabdodon septimanicus* Buffetaut & Le Loeuff, 1991.

#### Holotype

**Fig. 5:**
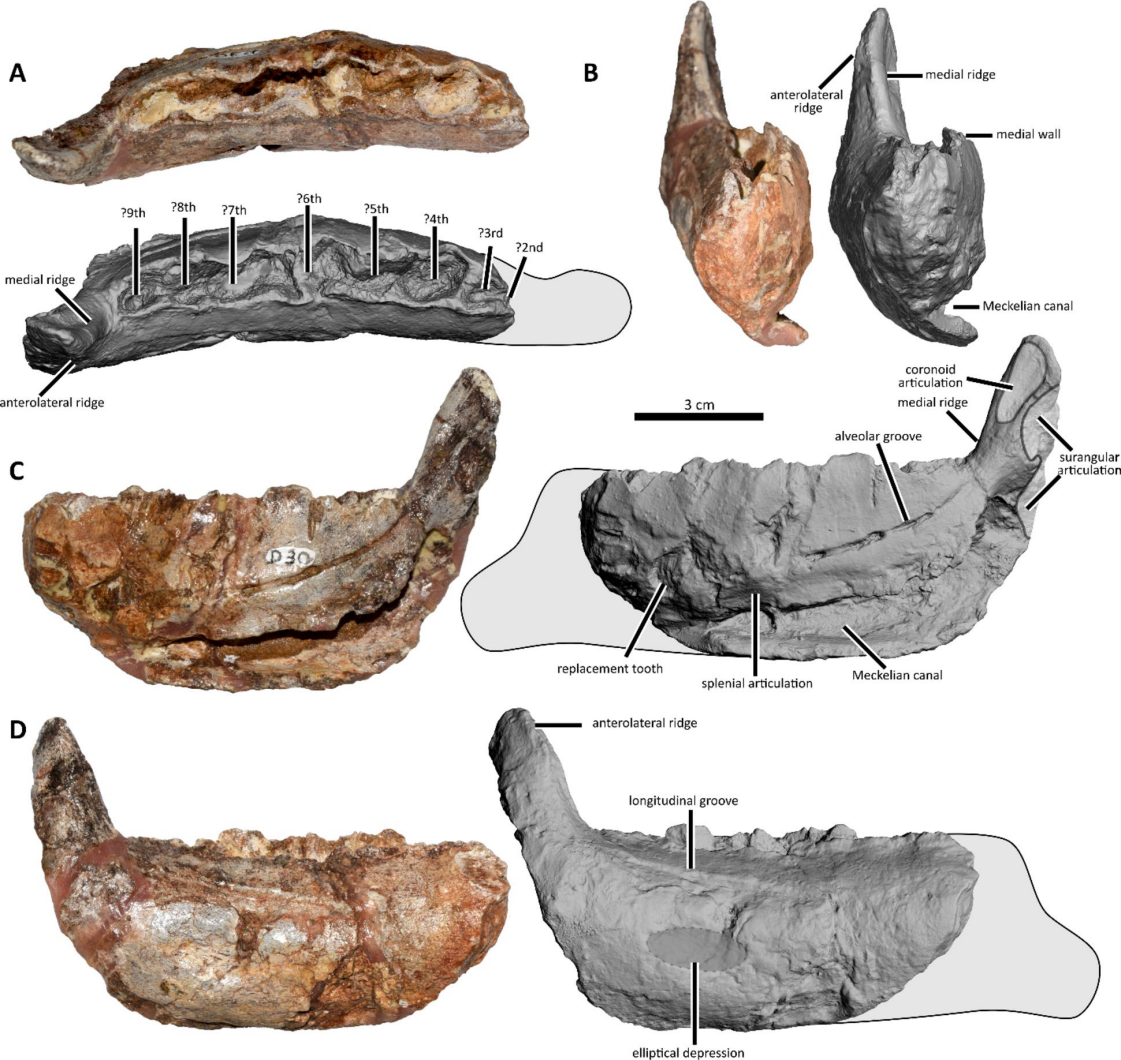
*Obelignathus septimanicus* gen. et comb. nov. holotype, MDE D30 from the ‘Grès à Reptiles’ Formation of Montouliers, southern France. Photographs and photogrammetric models of the right dentary in dorsal (**A**), anterior (**B**), medial (**C**), and lateral (**D**) views.

**Fig. 6:**
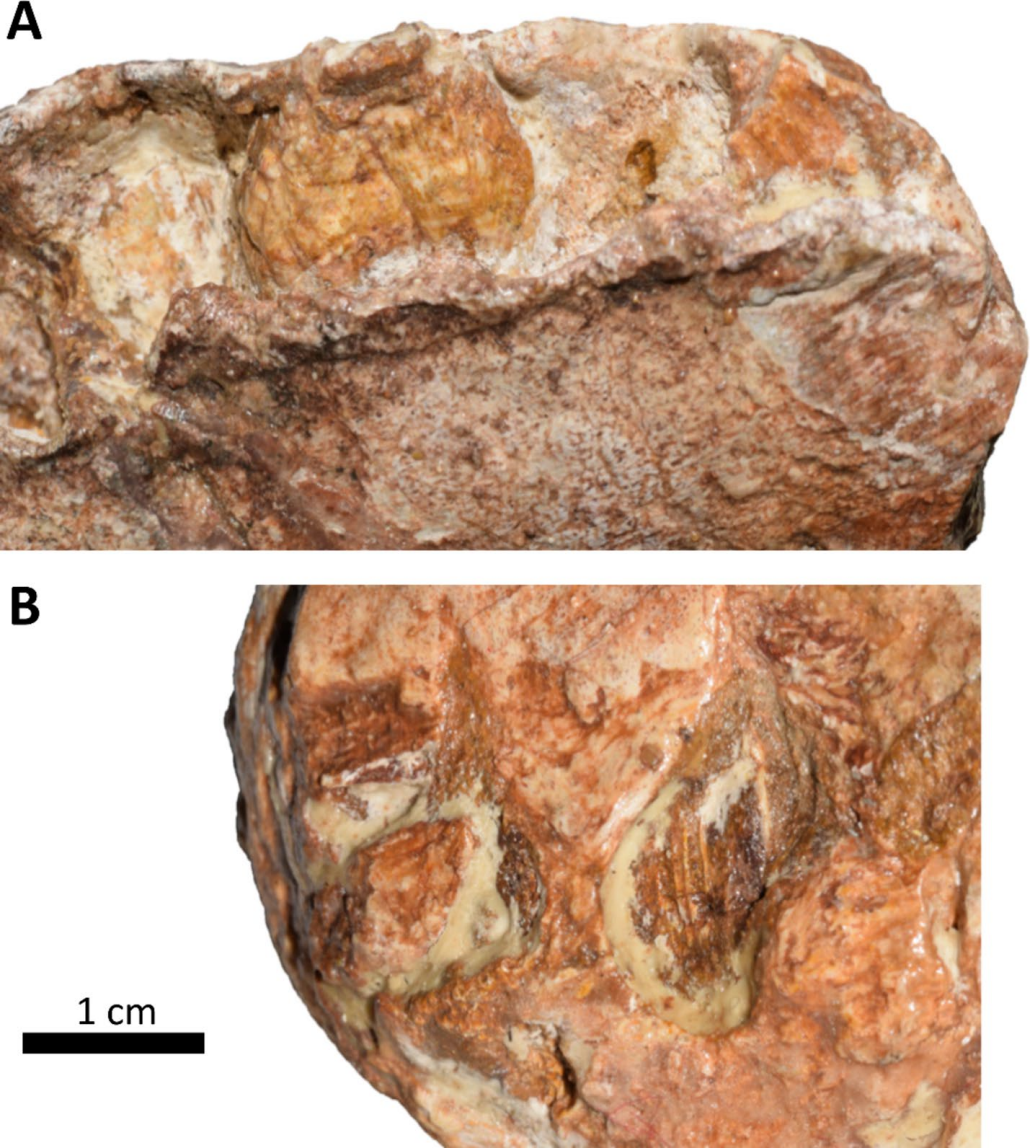
*Obelignathus septimanicus* gen. et comb. nov. holotype, MDE D30, preserved dentition. (**A**) Replacement teeth preserved in the ?3rd and ?5th alveoli in buccal view; (**B**) Replacement tooth preserved below the alveolar groove in lingual view.

**Fig. 7:**
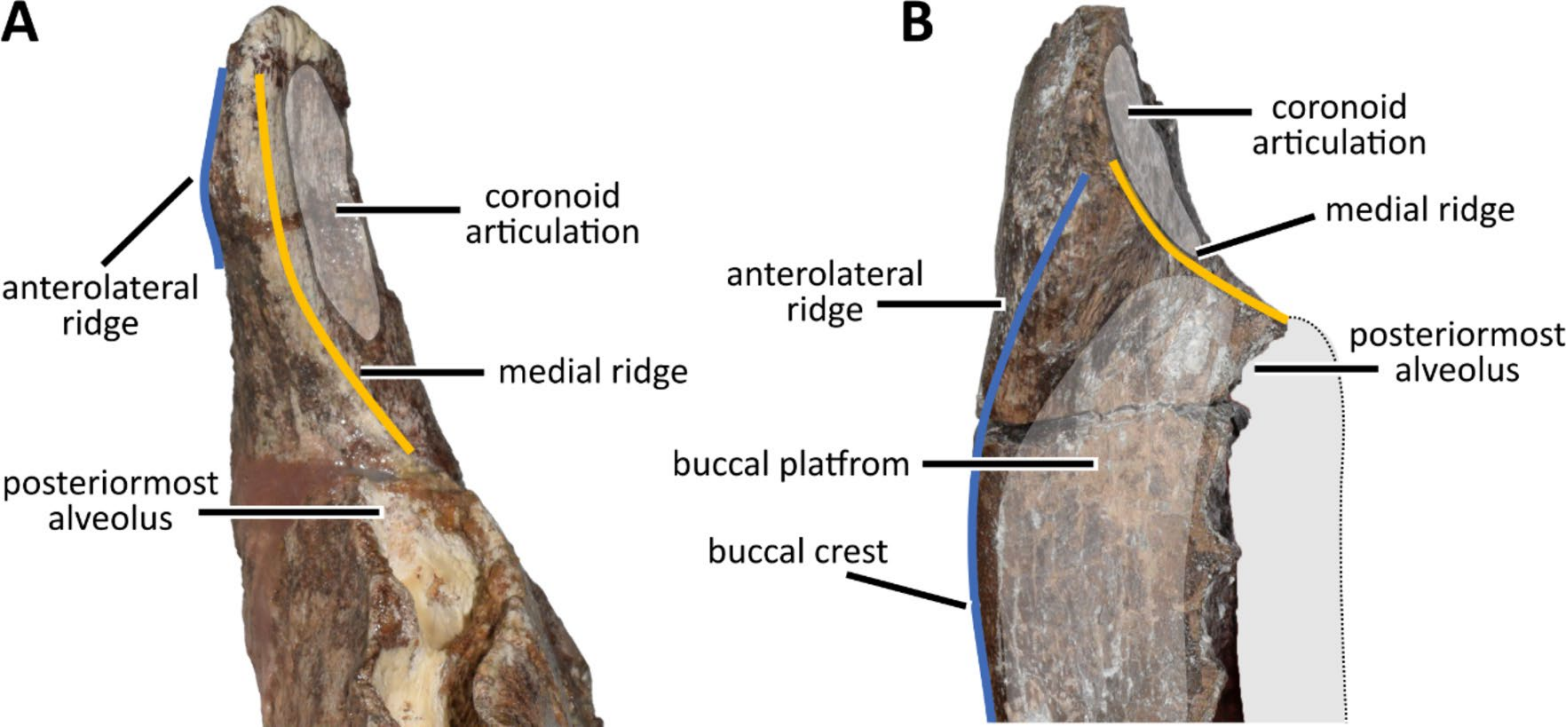
Coronoid process in rhabdodontomorphs. Lateroposteriorly rotated coronoid process of *Obelignathus septimanicus* gen. et comb. nov. MDE D30 (**A**) and typical rhabdodontoid condition seen in *Mochlodon vorosi* MTM V 2010.104.1 (**B**); both in anterodorsal view. In contrast to *O. septimanicus*, in rhabdodontoids the anterolateral ridge of the coronoid process is well-developed and forms a buccal crest that anteriorly and laterally delimitates the buccal platform. The medial ridge forms the anterior margin of the coronoid articulation and connects the coronoid process with the posterior part of the tooth row. Not to scale.

MDE D30 (earlier the Laboratoire de Paléontologie des Vertébrés, Universite Paris-VI, n° MTL 02^19^), a right dentary missing the anteriormost portion (Figs. 2 A,5,6,7 A).

#### Locality

Montouliers, Saint-Chinian commune, Hérault, southern France.

#### Horizon

‘Grès à Reptiles’ Formation (upper Campanian-lower Maastrichtian, Upper Cretaceous^53,54^).

#### Revised diagnosis

A medium-sized rhabdodontomorph dinosaur with the following unique combination of characters (*previously identified autapomorphy; **newly identified autapomorphy):

1. Dentary bone with a robust appearance, being anteroposteriorly shorter (with the main body of the dentary being about 3 times longer than high), labiolingually wider, and dorsoventrally higher than in other rhabdodontomorphs (where the dentary is about 4 times longer than high);
2. Absence of the buccal platform lateral to the alveolar row^19^;
3. Absence of the horizontal buccal crest in the posterior part of the dentary due to the poorly developed anterolateral ridge of the coronoid process;
4. Coronoid process of the dentary placed directly posterior to the alveolar row^19^, resulting in contact with a surangular being in line with the alveolar row and not displaced laterally as in other rhabdodontomorphs;
5. Coronoid process rotated lateroposteriorly, such as the lateral and coronoid surfaces of the process face, respectively, more posteriorly and more anteriorly than in other rhabdodontomorphs;
6. Meckelian canal mediolaterally and dorsoventrally deep, reaching 50% of the height of the medial wall of dentary below the alveolar groove (in other rhabdodontomorphs the dorsoventral height of the Meckelian canal is up to 33% of the medial height of the dentary wall).

### Description and comparisons

MDE D30 is a right dentary bone missing the anteriormost portion of the tooth row and the symphysis. The preserved part is 105.5 mm long anteroposteriorly, with dorsoventral height (measured anterior to the coronoid process) equal to 44 mm. In overall morphology, the dentary is robust, being relatively deep and short. When compared to other known rhabdodontomorph dentaries, the estimated length of the MDE D30 would be 130–140 mm (see Supplementary Information 1).

In dorsal view, the lateral wall of the dentary is slightly concave and the medial wall is strongly convex. This feature was suggested by Buffetaut & Le Loeuff^19^ as a possible diagnostic feature of *O. septimanicus* and is also observed in one dentary from the La Boucharde site^22^ (note that no collection number was given in the original paper; hence, hereafter we refer to this dentary as “La Boucharde dentary 2”) and some dentaries from the Quarante site^23^. In rhabdodontids, such as *R. priscus*, *Mochlodon* spp., and *Zalmoxes* spp., the lateral side of the bone is nearly straight in dorsal view, and the curvature of the medial wall is visible mostly in the anteriormost part of the tooth row.

**Fig. 8:**
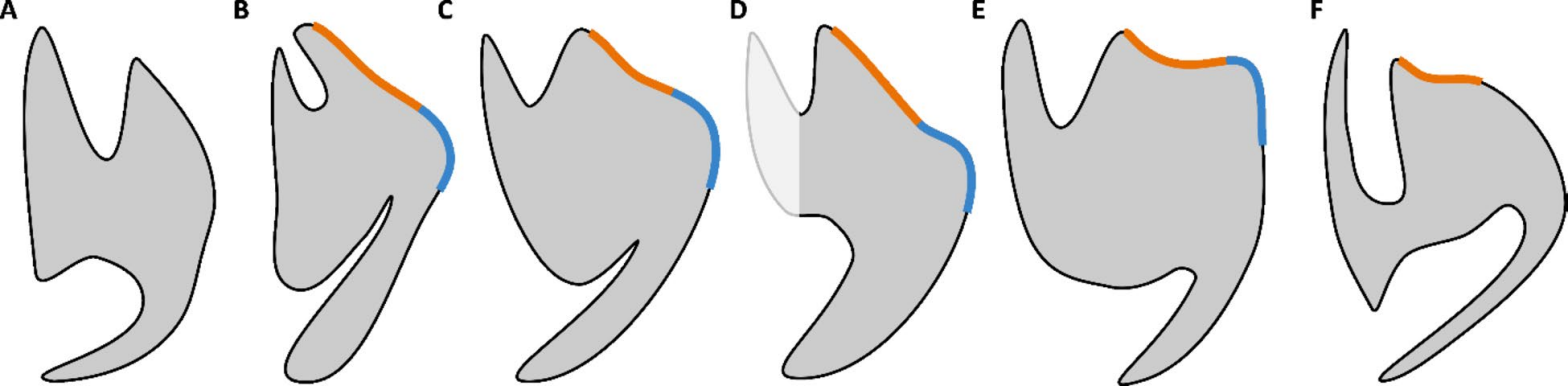
The transverse cross-section of the posterior part of the dentary bones (at the level anterior to the coronoid process) within rhabdodontomorphs shows the absence of the buccal platform (orange) and buccal crest (blue) in *Obelignathus septimanicus* gen. et comb. nov. MDE D30 (**A**). **B**) *Rhabdodon priscus* MLPM 30 (modified after Matheron, 1869), **C**) *Mochlodon suessi* PIUW 2349/2; **D**) *Mochlodon vorosi* MTM V 2010.105.1; **E**) *Zalmoxes shqiperorum* UBB NVZ1-1; **F**) *Tenontosaurus tilletti* OMNH 58340. Not to scale.

The lateral surface is smoothly convex dorsoventrally, without forming a well-defined flat shelf of the buccal platform lateral to the tooth row, which is otherwise present in all rhabdodontoids (*Rhabdodon priscus*,* Mochlodon* spp., *Zalmoxes* spp.; Fig. 8) and even in earlier-diverging members of Rhabdodontomorpha (*Iani smithi* NCSM 29373^55^ and the Vegagete rhabdodontomorph MDS-VG,16/17/152^5^). The other rhabdodontomorph specimen lacking the buccal platform is the “La Boucharde dentary 2”^22^ and such a condition was also reported for a dentary from the Quarante site^23^.

In dorsal view, the dentary of *O. septimanicus* has a constant lateromedial width. In contrast, rhabdodontids show variation in the maximum width of the dentary due to the development of a buccal platform. For example, the lectotype of *Rhabdodon priscus* (MPLM 30) and the lectotype of *Mochlodon suessi* (PIUW 2349/2) reach the greatest width at their midlength, while in *Mochlodon vorosi* and *Zalmoxes* spp. the dentary is widest in its posterior part.

There is no buccal emargination in *O. septimanicus*. In all rhabdodontids, including *R. priscus* (MPLM 30), *M. suessi* (PIUW 2349/2), *M. vorosi* (e.g., MTM V. 2010.105.1), and *Zalmoxes* spp., a buccal crest is formed by the horizontal expansion of the anterolateral ridge of the coronoid process (Fig. 8). Such a ridge is only poorly developed in *O. septimanicus*, and is directed lateroventrally, rather than anterolaterally (Fig. 7).

The completely preserved coronoid process of MDE D30 forms an angle of about 103° with the dorsal edge of the labial alveolar wall in lateral view. In the lectotype of *R. priscus*, the process slopes backwards much more strongly (with an angle of 146°). However, this feature seems to vary intraspecifically within the sample of rhabdodontids^19^. Within the known material of *Mochlodon* spp. and *Zalmoxes* spp., there are specimens with both posteriorly sloping coronoid processes (e.g., *Mochlodon vorosi* MTM PAL 2012.15.1, MTM PAL 2012.16.1, and *Zalmoxes* spp. NHMUK R3392, NHMUK R4912, LPB (FGGUB) R.2349) as well as with a nearly perpendicular one (e.g., *Mochlodon vorosi* MTM V 2010.105.1, *Zalmoxes* spp. NHMUK R3407, and ‘K2 rhabdodontid’ LPB (FGGUB) R.2777), without clear separation between the morphotypes.

The coronoid process of MDE D30 develops directly posteriorly to the alveolar row^19^. It is a condition contrasting that present in all later-diverging rhabdodontoids (*Rhabdodon priscus*,* Mochlodon* spp., *Zalmoxes* spp., and ‘K2 rhabdodontid’), where the coronoid process develops anteriorly and laterally to the posteriormost alveoli, such as that these are hidden by the coronoid process in lateral view. In all those taxa, the buccal wall of the last alveolus is positioned medially to the lingual surface of the coronoid process. In *R. priscus* MPLM 30 the posteriormost alveoli are placed at the base of the medial surface of the coronoid process. It further differs from *Mochlodon* spp. and *Zalmoxes* spp. in which the posteriorly extended buccal platform clearly separates the alveolar row and the coronoid process (this condition is especially pronounced in *Z. shqiperorum* NHMUK R4900). In contrast, in *O. septimanicus* the posteriormost alveolus is placed anteriorly to the coronoid process (Figs. 5 A, 7 A). A similar condition is also observed in the “La Boucharde dentary 2”^22^ and was also reported for specimen MC-CY_QR 1 from Quarante^23^.

There are two ridges present on the coronoid process in rhabdodontomorph dentaries (Fig. 7). In MDE D30, the medial ridge develops anteriorly towards the medial wall of the alveolar row and is confluent with the latter (Fig. 7A). In rhabdodontoids, this ridge also connects with the alveolar row, but due to its position, it develops medially (Fig. 7B). Posterior to this ridge on the coronoid process there is a flat surface covered with striations. It was suggested that it is a surface for muscle insertions, likely for the *M. pseudotemporalis*^56,57^; however, as is seen on the more complete rhabdodontoid specimens (e.g., *Zalmoxes shqiperorum* UBB NVZ1-1), the major part of this striated surface was covered by a tightly appressed coronoid bone^58^. In MDE D30, the striated medial surface covers only the dorsal half of the coronoid process, while in *Mochlodon vorosi* and *Zalmoxes* spp. it is almost reaching the level of the alveolar row.

The second ridge on the coronoid process in MDE D30 is poorly developed and expands anteroventrally for a short distance along the lateral surface of the process. In *R. priscus* (MPLM 30), *Mochlodon* spp. and *Zalmoxes* spp. such an anterolateral ridge develops anteriorly into the longitudinal buccal crest, laterally bordering the buccal platform (Fig. 7B). Posterior to this ridge, there is a relatively flat area with striations in MDE D30, that corresponds to the insertion surface for the *M. adductor mandibulae externus profundus*^56,57^.

As a result, the coronoid process of *O. septimanicus* is lateromedially narrow and rotated lateroposteriorly in relation to the longitudinal axis of dentary when compared with other rhabdodontomorphs (Fig. 7A). The medial surface of the coronoid process for contact with coronoid bone is nearly parallel to the tooth row, while the anterior surface is facing more laterally than in rhabdodontids. The contact with the surangular is positioned medially to the lateral wall of the alveolar row in *O. septimanicus*. This contrasts with the condition seen in all rhabdodontids (even in the lectotype of *R. priscus* MPLM 30), where the contact with the surangular is placed more laterally. Apart from MDE D30, such a posterolaterally rotated coronoid process is present only in the “La Boucharde dentary 2”^22^.

No clear track of the nutrient foramina is visible in MDE D30, although one foramen piercing the dentary at the level of the ?7th alveoli is poorly preserved^19^, being relatively smaller than those in other rhabdodontomorphs. The foramen lies within an anteroposterior groove developing anteroventrally at the level of posteriormost five alveoli (Fig. 5D), but which is most likely caused by some taphonomical damage^19^. However, notably, a similarly positioned groove connecting the nutrient foramina is visible in some dentaries of *Zalmoxes* spp.(e.g., LPB (FGGUB) R.1640, LPB (FGGUB) R.2411; NHMUK R3406).

Ventral to this longitudinal groove there is a shallow, but clearly delimited elliptical depression (Fig. 5D). Although it also may be caused by some taphonomic distortion of the specimen, it is again worth noting that a similar depression was reported in one specimen of the ‘K2 rhabdodontid’ (LPB (FGGUB) R.2778^51^). Seemingly a similar fossa, but placed more anteriorly, is also present in the “La Boucharde dentary 2”^22^, although this needs to be confirmed by personal examination.

On the medial side, the alveolar groove is developed in the form of a distinct furrow. In MDE D30 it expands posterodorsally, resulting in the posterior part of the groove being terminated anterior to the coronoid process and dorsally reaching the level of the posteriormost tooth (Fig. 5C). This feature seems to be variable in rhabdodontoids, e.g., in *Rhabdodon* sp. CM 443 the alveolar groove develops ventrally, never reaching the dorsal portion of the dentary and does not terminate anterior to the coronoid process^14^, while in *Zalmoxes robustus* NHMUK R3407 the condition is similar to that observed in *O. septimanicus* MDE D30.

The Meckelian canal seems to be very well developed, dorsoventrally high and mediolaterally deep. In MDE D30, the Meckelian canal reaches up to 50% of the dorsoventral height of the dentary anteriorly to the coronoid process. The ratio seems to be much greater than in the majority of other rhabdodontomorph dentaries (22.5–38.5% in *Zalmoxes* spp., 28.6–33.3% in *Mochlodon* spp., 27.8–38.0% in *Rhabdodon* sp., and 31.8% in *Tenontosaurus tilletti*), with only one other rhabdodontomorph dentary from France (the “La Boucharde dentary 2”^22^) being of a similar dorsoventral height (47.4% of the dentary height) as that present in *O. septimanicus*.

Just above the Meckelian canal, the medial wall of the dentary bears a longitudinal groove. It most likely represents a contact surface for the unpreserved splenial bone. Its anteriormost extent is unknown because of the incompleteness of the specimen.

Eight posterior alveoli are visible, with the anteriormost one being only partially preserved. The third to seventh preserved alveoli are similar in size, with the first two and the eighth one being significantly smaller. In rhabdodontids, the first two (e.g., *Zalmoxes* spp. NHMUK R3392, NHMUK R4912, SZTFH Ob.3065, UBB NVZ1-1) or three (e.g., *Mochlodon suessi* PIUW 2349/2; *Mochlodon vorosi* MTM V 2010.105.1, MTM PAL 2012.16.1; *Zalmoxes* spp. NHMUK R3407, LPB R.0088) alveoli are usually significantly smaller than the following ones. This suggests that originally there were most probably eight or nine dentary alveoli in MDE D30, and only a small anteriormost portion of the dentary is broken away.

Although incompletely preserved, the medial wall of MDE D30 seems to reach higher dorsally than the lateral one^19^. Although this condition can be compared only with a few specimens that preserved the medial parapet of the dentary, the medial wall is seemingly nearly at the same level as the lateral wall in *Zalmoxes robustus* LPB (FGGUB) R.1372 and *Mochlodon vorosi* MTM V 2010.104.1.

In dorsal view, the alveolar row is relatively curved, with a concave labial margin. Buffetaut & Le Loeuff^19^ considered it to be a condition different from that seen in rhabdodontids. However, this feature is difficult to quantify, as the level of alveolar curvature seems to differ within the sample of a particular taxon, with, e.g., the majority of specimens of *Mochlodon vorosi* having only the anteriormost part of the alveolar row laterally arched, while it is being arched medially in some specimens of *Zalmoxes* spp. (e.g., *Z. robustus* LPB (FGGUB) R.1371) but straight in others (e.g., *Z. robustus* NHMUK R4912, *Z. shqiperorum* UBB NVZ1-1). Nevertheless, the curvature seen in specimen MDE D30 seems to be stronger than in the majority of other rhabdodontomorphs. A similar degree of curvature is present in the “La Boucharde dentary 2”^22^, and it is even more pronounced in specimen MC-CY_QR 1 from the Quarante site^23^.

As observed by Buffetaut & Le Loeuff^19^, the interdental plates between alveoli are incompletely preserved, suggesting that they were only partially ossified, similar to the condition reported in rhabdodontoids. Replacement teeth are present in several alveoli (2nd, 3rd, 5th, 6th, 8th and 9th from those preserved; Fig. 6A). One replacement tooth is also visible ventral to the alveolar groove below the ?3rd alveoli (Fig. 6B). The teeth are roughly similar to those of rhabdodontoids, with a prominent primary ridge and a number of secondary ridges as in *R. priscus* and *Mochlodon* spp. but fewer than in *Zalmoxes* spp. Unfortunately, other features of the dentary teeth in MDE D30 (e.g., number of ridges) are indiscernible, because of the incomplete exposure of the teeth.

 Rhabdodontomorpha Dieudonné et al., 2016 [Madzia et al., 2021].

 Rhabdodontoidea Poole, 2022 [Fonseca et al., 2024].

 Rhabdodontidae Weishampel et al., 2003 [Madzia et al., 2021].

*Rhabdodon* Matheron, 1869.

*Rhabdodon priscus* Matheron, 1869 (amended Brinkmann, 1986).

#### Lectotype

**Fig. 9:**
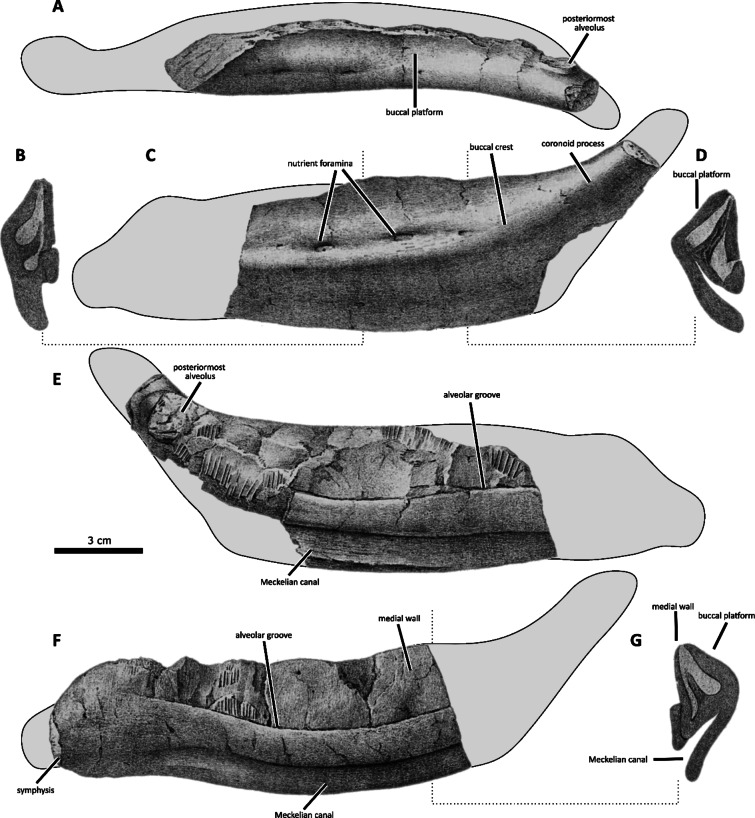
*Rhabdodon priscus* Matheron, 1869, lectotype and paralectotype dentaries from the Lower Rognacian lacustrine marls of ‘Tunnel de la Nerthe’, southern France. Lectotype left dentary (MPLM 30) in dorsal (**A**), lateral (**C**) and medial (**E**) views, with cross-sections in its anterior (**B**) and posterior (**D**) parts. Paralectotype right dentary (MPLM 31) in medial view (**F**), and cross-section of the posterior portion (**G**). Modified after Matheron^18^.

MPLM 30, a partial left dentary missing the anterior portion (Figs. 2E and 9).

#### Referred material

MPLM 31, an anterior part of the right dentary (paralectotype); MPLM 32, a dentary fragment (paralectotype); MPLM 34, a posterior dorsal vertebra (paralectotype); MPLM 36, two fused sacral vertebrae (paralectotype); MPLM 51, left radius (paralectotype); MPLM 59, proximal end of the right femur (paralectotype); MPLM 60, distal part of the right tibia (paralectotype); MPLM 61, distal end of a right femur (paralectotype); two posterior caudal vertebrae (no collection number given; paralectotypes)^39,41,59^.

#### Locality

“Tunnel de la Nerthe”, on the Avignon-Marseille railroad line between Marignane and L’Estaque (northwest of Marseille), Bouches-du-Rhône, Provence-Alpes-Côte d’Azur, southern France^18,39^.

#### Horizon

Early Rognacian lacustrian marl (lower Maastrichtian, Upper Cretaceous^19,41,59,60^).

#### Revised diagnosis

The lectotype of *Rhabdodon priscus* (MPLM 30) differs from other rhabdodontomorphs by the following unique combination of characters: (1) presence of a buccal platform (shared with *Iani smithi*, *Tenontosaurus* spp., *Mochlodon* spp., and *Zalmoxes* spp.); (2) transversely sharp buccal crest of the dentary (shared with *Mochlodon* spp. and *Zalmoxes* spp.); (3) posteriormost alveolus placed medially to the coronoid process, but not separated by a buccal platform posteriorly (shared with *I. smithi* and *Tenontosaurus* spp.); (4) absence of a lateral depression on the posterior portion of the dentary, below the coronoid process (shared with *I. smithi*, *Tenontosaurus* spp., *Zalmoxes* spp., and *O. septimanicus*).

#### Remarks

The lectotype and paralectotype dentaries deteriorated since their discovery, and were not found in the MPLM collection during a visit to Marseille in 2023; hence, the diagnosis provided herein is based on illustrations by Matheron^18^ and a photograph from Brinkman^39^. The latter shows that the posterior part of the specimen, including the coronoid process, was broken off already in the 1980s; the condition of the material further worsened thereafter^41^. Matheron^61^ and Lapparent^35^ referred additional material to this species. More recently, Garcia et al.^59^ and Pincemaille-Quillevéré^41^ described new specimens from de Vitrolles as belonging to this taxon, and provided a detailed diagnosis including features of the postcranial elements, while Allain and Pereda Suberbiola^22^ and Chanthasit^23^ referred even further specimens to this species. The once outstanding cranial features (e.g., maxillary teeth with parallel ridges without prominent primary ridges; dentary teeth with prominent distal primary ridges; enamel thicker on the buccal side of the maxillary and lingual side of the dentary teeth) listed in the earlier emended diagnoses^41,59^ are in fact synapomorphies of rhabdodontomorph dinosaurs (present also in *Mochlodon* spp., *Zalmoxes* spp., *Obelignathus septimanicus*, and *Iani smithi*).

 A large number of historical specimens from Eastern Europe were referred at one point to *Rhabdodon priscus*, including at least part of the material currently referred to *Zalmoxes robustus*,* Z. shqiperorum* and *Mochlodon suessi*^13,17,26,32,39,44,62^. Over the years additional western European specimens from several sites from France^22,35^,^41,59–61,63,64^ and also from Spain^65–69^ were referred to this species, or at least to the genus *Rhabdodon*. However, this latter material requires a thorough revision; as such, these taxonomic referrals should be treated with caution.

## Discussion and conclusions

### The phylogeny and nomenclature of rhabdodontomorph clades

#### The phylogeny of Rhabdodontomorpha

The intrarelationships within the *Rhabdodon*-lineage iguandodontians have been assessed multiple times, yielding varying outcomes. Traditionally, *Rhabdodon priscus* and its closest relatives, including *Mochlodon* spp. and *Zalmoxes* spp., have been united within Rhabdodontidae, a clade endemic to the Late Cretaceous European archipelago^13,14,70^. However, given the young age of these ‘core’ rhabdodontids combined with their proposed phylogenetic placement as some of the earliest-diverging iguanodontians, it has been expected that they formed part of a more inclusive branch with at least Early Cretaceous roots. Several taxa have been suggested to be placed as earlier representatives of the *Rhabdodon* lineage. The best candidates include the Early to ‘middle’ Cretaceous taxa *Muttaburrasaurus langdoni*^5,6,8,49^, the ‘Vegagete ornithopod’^5,6,9,12^, *Fostoria dhimbangunmal*^6,55^, *Tenontosaurus* spp.^12,49,55,71^, and *Iani smithi*^12,55^. The most recent addition to the list, but whose phylogenetic placement has not been tested beyond the original descriptive study, is *Emiliasaura alessandrii* from the Valanginian (Lower Cretaceous) of Patagonia, Argentina^49^.

New Late Cretaceous rhabdodontomorphs have been described in recent years from Europe as well. These include the possible non-rhabdodontid rhabdodontoid *Transylvanosaurus platycephalus*^16^ and the probable rhabdodontids *Matheronodon provincialis*^20^ and *Pareisactus evrostos*^21^. However, although they clearly show characters present in rhabdodontoid-line rhabdodontomorphs, the fragmentary nature of their material makes them difficult to be reliably assessed from a phylogenetic perspective.

We provided a phylogenetic assessment of all of the taxa mentioned above using the character matrix of Fonseca et al.^12^, rescoring several of them and also including the recently-described *Ampelognathus coheni* and *Emiliasaura alessandrii*. We have also applied different settings. As in Fonseca et al.^12^, we conducted parsimony analyses using both equal weights and several runs with the implied weighting function. However, the weighted parsimony analyses of Fonseca et al.^12^ employed a much stronger downweighting of homoplastic characters, utilizing *K*-values of 3, 7, and 11. While the strong concavity *K* = 3 remains the default value in TNT 1.6, it is likely way too low for a character matrix of this size^72^.

Our analyses did not yield uniform results but generally supported the monophyly of Rhabdodontomorpha. The findings suggest that rhabdodontomorphs diverged early in their evolutionary history into two clades: one distributed in North America, comprising *Convolosaurus marri*, *Iani smithi*, and *Tenontosaurus* spp.; and another potentially endemic to the European archipelago. While the topology of the North American clade (Tenontosauridae) remained stable across analyses, the intrarelationships within the European branch (Rhabdodontoidea) were poorly resolved, likely due to the limited knowledge of its members, many of which are represented by very fragmentary remains or require thorough revision.

As in Fonseca et al.^12^, our analyses found the Australian taxa *Muttaburrasaurus langdoni* and *Fostoria dhimbangunmal* to be nested outside Rhabdodontomorpha. Meanwhile, the recently-established *Emiliasaura alessandrii*, which was originally inferred to be the earliest-diverging rhabdodontomorph^49^ was consistently reconstructed as an early-diverging styracosternan by all our analyses using implied weighting. The analysis using equal weights was less conclusive but also preferred dryomorphan affinities for the taxon. It is worth noting, however, that the *E. alessandrii* OTU was scored based on the description and figures from Coria et al.^49^ and some of its characters are obscured. As such, the precise placement of the taxon requires further investigation. Still, we consider it unlikely to be a rhabdodontomorph.

 The recently-described *Ampelognathus coheni* from the Cenomanian of North America was originally reconstructed within Ornithopoda as the sister taxon to *Thescelosaurus* and Iguanodontia^48^. However, Fonseca et al.^12^ suggested that it may be a rhabdodontomorph instead. Our weighted parsimony analyses support the latter hypothesis, inferring *Ampelognathus* within Rhabdodontomorpha though its precise placement is uncertain. *Ampelognathus* shares with *Obelignathus* the relatively short dentary, arched medial wall of the tooth row, and absence of the buccal crest. Additionally, as in tenontosaurids and *Rhabdodon priscus*, the posteriormost dentary alveolus in *A. coheni* is placed laterally to the coronoid process but not separated from it by a buccal platform posteriorly.

####  The phylogenetic placement of *Obelignathus septimanicus*

Our analyses provided two alternative hypotheses for the placement of *O. septimanicus*: as an early-diverging rhabdodontomorph (under *K* = 15 and 21) or as an early member of the elasmarian lineage (under *K* = 12). The analysis using equal weights was inconclusive but closer inspection of the MPTs showed that it also placed the taxon among early members of the elasmarian lineage.

**Fig. 10:**
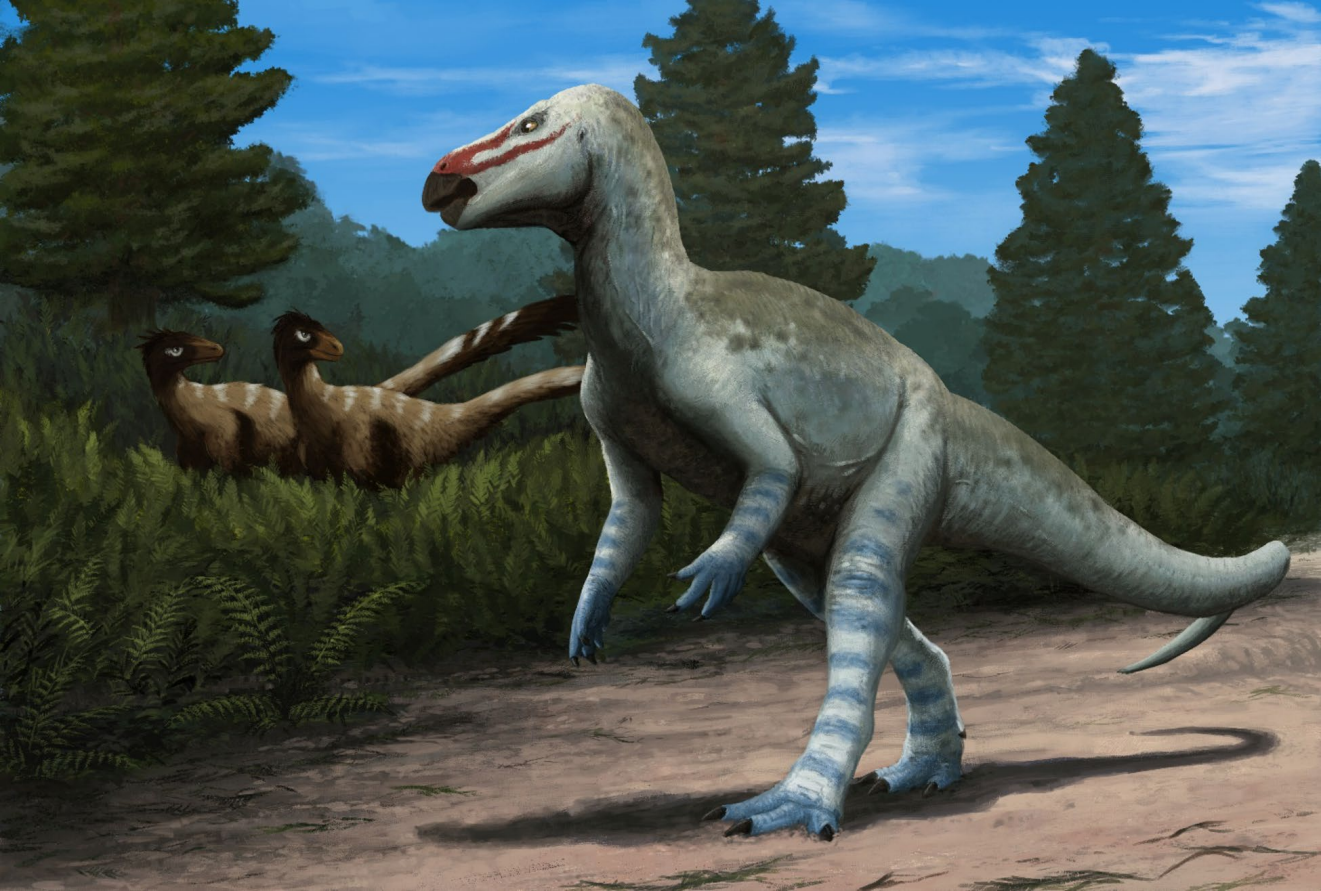
Life restoration of *Obelignathus septimanicus* gen. et comb. nov. in the Late Cretaceous environment recorded in the ‘Grès à Reptiles’ Formation, with a pair of dromaeosaurid dinosaurs in the background. Artwork by Edyta Felcyn-Kowalska (CC BY 4.0).

Although problematic and clearly distinct in many morphological features from rhabdodontoids, we hypothesize that the uncertain position of *O. septimanicus* is most likely due to the highly incomplete nature of its type material. While further phylogenetic studies and potentially the discovery of new specimens referable to the taxon are needed, we are inclined to consider *O. septimanicus* a probable rhabdodontoid, with its dentary morphology likely shaped by a feeding ecology different from that of other clade members (Fig. 10).

#### The nomenclature of Rhabdodontomorpha

Following Article 11.10 of the *International Code of Phylogenetic Nomenclature*^73^, “when a clade name is converted from a preexisting name that is typified under a rank-based code or is a new or converted name derived from the stem of a typified name, the definition of the clade name must use the type species of that preexisting typified name or of the genus name from which it is derived (or the type specimen of that species) as an internal specifier”. Therefore, owing to the fact that the clade names Rhabdodontomorpha, Rhabdodontoidea, and Rhabdodontidae are derived from the stem of *Rhabdodon* Matheron, 1869, *R. priscus* must be an internal specifier in their phylogenetic definitions. The recent studies that provided formal phylogenetic definitions of Rhabdodontomorpha and Rhabdodontidae[24], as well as Rhabdodontoidea^12^, met this requirement. However, as the type material of *Rhabdodon priscus* has not been studied in detail on its own in recent years, it remains unclear which taxa these clade names, particularly Rhabdodontidae, encompass.

We have investigated the history of the lectotype and paralectotype specimens of *R. priscus*, compared it with other European rhabdodontoids, and rescored its OTU based on the type material only. Although highly fragmentary and unavailable for personal examination when we attempted to study it, the material is clearly nested among late-diverging rhabdodontoids and its position within the clade is comparatively stable, removing all doubts regarding its usefulness as a ‘phylogenetic anchor’ for the names aside.

####  The history of *Obelignathus septimanicus*

 Originally, specimen MDE D30 was referred to the genus *Rhabdodon* based on the overall similarities in their teeth morphology, yet it was distinct from all other dentaries referred at that time to the species *Rhabdodon priscus*^19^. It is worth emphasizing that back in 1991, *R. priscus* was the only rhabdodontid species recognized as valid, and within the material then referred to *R. priscus* were dentaries from France, Austria, and Romania, that currently belong to several distinct taxa (*R. priscus*, *Mochlodon suessi*, *Zalmoxes robustus* and *Z. shqiperorum;* Fig. 1).

 Subsequently, *Rhabdodon septimanicus* was considered a junior synonym of *Rhabdodon priscus* by Allain & Pereda Suberbiola^22^ who noticed the presence of two rhabdodontid dentary morphotypes (one of them, the “La Boucharde dentary 2”, with several diagnostic features of *R. septimanicus*) within the rhabdodontid sample collected from the La Boucharde site, but simultaneously have not observed any disparity within the abundant rhabdodontid postcranial material from the same locality. They suggested that the distinction between *R. septimanicus* and *R. priscus* is a matter of individual differences or sexual dimorphism^22^.

 However, the taxonomic distinction between the two species was once again recognized by later authors^13,42,58^. Chanthasit^23^ not only accepted the validity of *R. septimanicus* but also referred a few new cranial elements from the Quarante site (Hérault) to this species. Ősi et al.^14^ also accepted the validity of *R. septimanicus* and, based on their histological analysis, pointed out the possibility that there were even as many as three or four distinct rhabdodontid taxa present in the Upper Cretaceous of France^15,43,63,74^.

 Our studies confirm the unusual morphology of MDE D30 that differentiates this specimen from all other dentaries of rhabdodontomorph dinosaurs.

 The “La Boucharde dentary 2”, a nearly complete dentary that seems to be more robust than the other dentary specimen collected from the same site, was referred to *R. priscus*^22^. Nevertheless, lacking the buccal platform and buccal crest, with the coronoid process being placed posteriorly to the alveolar row, and not being laterally separated from the latter, as well as displaying a posterolaterally rotated coronoid process, it seems plausible that this dentary specimen represents the same species as MDE D30. However, as the specimen was not available for personal examination, the referral of this dentary to *O. septimanicus* should await its detailed description.

 Chantasit^23^ referred several specimens from the Quarante site (southern France) as potentially belonging to the same species as MDE D30; from these, the only illustrated dentary is specimen MC-CY_QR 1. Although the curvature of this dentary in dorsal view seems to be even more pronounced than that of MDE D30, the bone itself is one of the most elongated ones within rhabdodontomorphs, with an even more clearly defined buccal platform, a coronoid process seemingly displaced laterally from the alveolar row, a low mandibular canal, and a coronoid process that is not rotated posterolaterally, in contrast to the condition seen in MDE D30. As this material was not available for personal examination either, we refrain from referring this specimen to any particular taxon until a detailed description is published.

####  The anatomy and feeding ecology of *Obelignathus septimanicus*

The estimated elongation of the MDE D30 dentary (3.045) is lower than that found in the majority of rhabdodontomorphs. Among the material from France, a similar elongation is visible in one large dentary from the La Boucharde site (“La Boucharde dentary 2”^22^, with an elongation equal to 2.59). Only one rhabdodontoid specimen, LPB (FGGUB) R.1523, a small dentary from Romania tentatively referred to *Zalmoxes robustus*, has a similar elongation ratio (3.02). However, this specimen differs from other *Zalmoxes* dentaries in, e.g., the morphology of the posterior part of the alveolar row, suggesting the high necessity of the taxonomic revaluation of all the rhabdodontid material from Romania.

 The buccal emargination is a common feature present in all neornithischian dinosaurs, hence the condition of *O. septimanicus* is a significant deviation within this group, most likely representing a reversal of this plesiomorphic condition.

 The posteriormost alveoli being placed anterior to the coronoid process is a feature that differentiates *O. septimanicus* from all currently known rhabdodontoid dentaries. Among Rhabdodontomorpha, a similar condition is present in *Tenontosaurus* spp., but not in *Iani smithi* and *Convolosaurus marri;* however, in all these taxa, the coronoid process is again laterally displaced from the tooth row^55,75,76^, in contrast to *O. septimanicus*. Among Ornithopoda, a coronoid process that is not laterally displaced from the tooth row was reported for iguanodontian *Iyuku raathi* from the Lower Cretaceous of South Africa^52^. Although it was suggested that in some taxa this feature might be an ontogenetically variable one, it would be consistently observed in both dentaries potentially belonging to *O. septimanicus* (MDE D30 and the “La Boucharde dentary 2”), while not being a variable feature within rhabdodontoids in general, as a coronoid process covering the posteriormost part of the alveolar row in lateral view is already present in even the smallest specimens of other taxa (*Mochlodon* spp. and *Zalmoxes* spp.).

Due to the posterolaterally rotated coronoid process of *O. septimanicus*, in contrast to the condition seen in the majority of ornithischian dinosaurs, the insertions for the *M. adductor mandibulae externus profundus* on the lateral surface of the coronoid process were facing more posteriorly relative to the axis of the tooth row. Meanwhile, in other European rhabdodontomorphs, this insertion area was oriented more laterally. Similarly, the medial surface for insertion of the *M. pseudotemporalis* is facing more anteriorly in MDE D30 than in rhabdodontoids. Hence, it suggests a relatively transversely wide posterior portion of the mandible in *O. septimanicus*. Together with poor development of the buccal crest, which most likely was an attachment site for *M. adductor mandibulae externus superficialis*^57^, this morphology may indicate quite different jaw mechanics of *O. septimanicus* compared to other rhabdodontomorphs^19^, including the material referred to *R. priscus* known from the same strata. A similar case of possible divergent feeding adaptations in sympatric taxa was suggested for rhabdodontids from Transylvania^15,16^. The proposed differences in jaw mechanics between *O. septimanicus* and the sympatric rhabdodontoid (e.g., material from the ‘Grès à Reptiles’ Formation preliminary referred to *R. priscus*) should be reflected in the wear patterns of teeth; hence, a closer investigation of the isolated teeth from the French sites in this regard would be highly desirable.

## Electronic supplementary material

Below is the link to the electronic supplementary material.

Supplementary Material 1

Supplementary Material 2

Supplementary Material 3

Supplementary Material 4

Supplementary Material 5

Supplementary Material 6

Supplementary Material 7

## Data Availability

All data generated or analysed during this study are included in this published article and its supplementary information files. The 3D model of the *Obelignathus septimanicus *gen. et comb. nov. holotype is available in MorphoSource (https://www.morphosource.org/concern/media/000697351).
