# Supplementary material for: Exploring the diversity and disparity of rhabdodontomorph ornithopods from the Late Cretaceous European archipelago
Source: Sci Rep. 2025 Apr 30;15:15209. doi: 10.1038/s41598-025-98083-z (PMC12044058; doi:10.1038/s41598-025-98083-z)
Supplement: Supplementary file 1 — Supplementary Material 1 [file 41598_2025_98083_MOESM1_ESM.docx]

**Supplementary Information I for:**

**Exploring the diversity and disparity of rhabdodontomorph ornithopods from the Late Cretaceous European archipelago**

Łukasz Czepiński and Daniel Madzia

**Characters list for principal coordinate and principal component analyses**

The total length of the partially preserved specimens was estimated based on the most similar specimens in the sample. For example, the original length of the lectotype dentary of *Rhabdodon priscus* (MPLM 30) was estimated based on the original specimens (MPLM 30 and MPLM 31) described by Matheron (1869) and the most recent photography of the type dentary (Brinkman 1988). In turn, the total length of the holotype of *Obelignathus septimanicus* gen. et comb. nov. (MDE D30) was estimated following comparisons with the “La Boucharde dentary 2” (Allain & Pereda Suberbiola, 2003:fig2:2).

The longer axis of the dentary is determined by the line connecting the six posterior alveoli of similar size, as usually the first two or three smaller alveoli are laterally displaced. For specimens that were not personally examined, the measurements were taken from photographs using the ImageJ software.


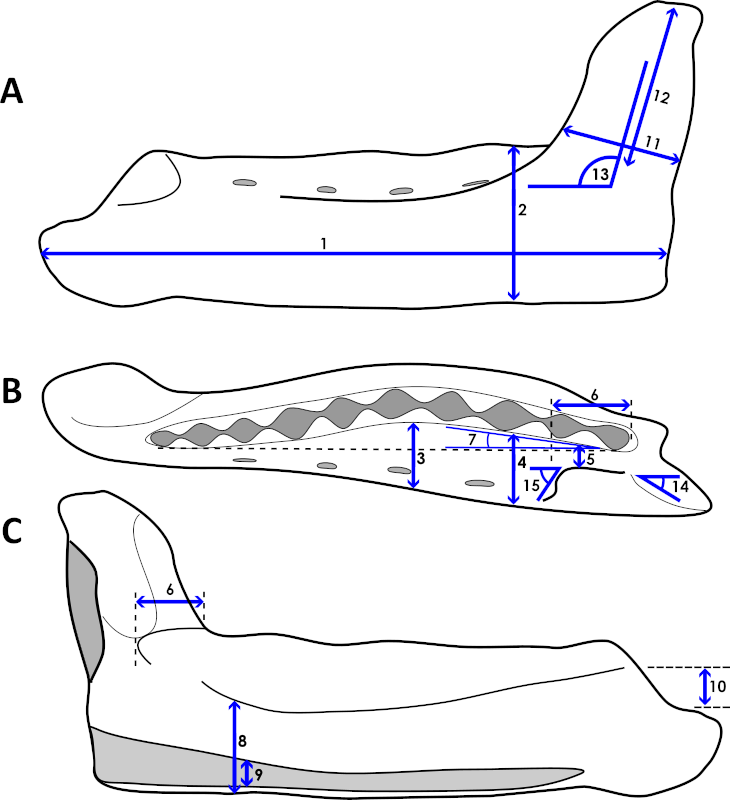


**Figure S1.1.** Drawing of the idealized rhabdodontoid dentary based on specimens referred to *Rhabdodon*, *Zalmoxes*, and *Mochlodon* in A) lateral, B), dorsal, and C) medial views.

**Continuous characters**

1. Total (or estimated total) anteroposterior length of the dentary (in mm)
2. Dorsoventral height at the level anterior to the coronoid process (in mm)
3. Buccal platform – midpoint mediolateral width (in mm)
4. Buccal platform – posterior mediolateral width at the level of the coronoid process (in mm)
5. Lateromedial distance between the alveolar row and the coronoid process (in mm)
6. Anteroposterior distance between the last tooth and the anterior margin of the coronoid process (in mm)
7. Angle between the medial margin of the medialmost located alveoli and the longer axis of the dentary (in degrees)
8. Dorsoventral height of the dentary below the alveolar groove anterior to the coronoid process (in mm)
9. Mandibular canal – dorsoventral height anterior to the coronoid process (in mm)
10. Dorsoventral distance between the tip of the dentary symphysis and the anterior portion of the alveolar groove in medial view (in mm)
11. Anteroposterior length of the coronoid process measured at the dorsal alveolar margin level, perpendicular to the longer axis of the coronoid process (in mm)
12. Dorsoventral height of the coronoid process measured above measured along the longer axis od the process, above the alveolar row (in mm)
13. Angle between the alveolar row and the midline longer axis of the coronoid process in lateral view (in degrees)
14. Angle between the medial surface of the coronoid process (coronoid articulation) and the longer axis of dentary in dorsal view (in degrees)
15. Angle between the anterior surface of the coronoid process and the longer axis of dentary in dorsal view (in degrees)

**Discrete characters**

1. Buccal platform: 0) absent; 1) present.
2. Buccal crest (= anterolateral ridge of coronoid process): 0) absent; 1) present.
3. Coronoid process, position regarding the last alveolus: 0) posteriorly; 1) laterally; 2) laterally & separated.
4. Lateral depression below the tooth row: 0) absent; 1) present.
5. Posterior depression below the coronoid process: 0) absent; 1) present.
6. Nutrient foramina, size: 0) small; 1) elongated.
7. Groove for the splenial, anterior range: posterior to the (tooth position): 0) 2nd; 1) 3rd; 2) 4th; 3) 5th or more posterior.
8. Coronoid, morphology: 0) small; 1) large; 2) large and L-shaped.

**References**

Allain, R. & Pereda Suberbiola, X. (2003). Dinosaurs of France. *Comptes Rendus Palevol* 2: 27–44.

Brinkmann, W. (1988). Zur Fundgeschichte und Systematik der Ornithopoden (Ornitischia, Reptilia) aus der Ober-Kreide von Europa. *Documenta Naturae,* 45: 1–157.

Matheron, P. (1869). Notice sur les reptiles fossiles des dépôts fluviolacustres crétacés du bassin à lignite de Fuveau. *Mémoires de l’Académie impériale des Sciences, Belles- Lettres et Arts de Marseille*, 1–39.
