# Supplementary material for: Exploring the diversity and disparity of rhabdodontomorph ornithopods from the Late Cretaceous European archipelago
Source: Sci Rep. 2025 Apr 30;15:15209. doi: 10.1038/s41598-025-98083-z (PMC12044058; doi:10.1038/s41598-025-98083-z)
Supplement: Supplementary file 5 — Supplementary Material 5 [file 41598_2025_98083_MOESM5_ESM.docx]

**Supplementary Information V for:**

**Exploring the diversity and disparity of rhabdodontomorph ornithopods from the Late Cretaceous European archipelago**

Łukasz Czepiński and Daniel Madzia

**Changes to the character scores for phylogenetic analyses**

*Obelignathus septimanicus* gen. et comb. nov.

Scores for non-mandible characters (1–301; 481–944) were set as “?”.

304: 1→? (anteriormost tip not preserved)

321: 1→0

322: 0→?

323: 1→?

324: 0→?

325: 0→?

326: 1→0 (tooth row bowed medially, last tooth row ends in the same axis as the coronoid process)

327: 2→?

328: 0→?

330: 1→?

332: 0→?

333: 0→? (the longitudinal groove is visible but most likely resulted from distortion of the specimen)

339: ?→0

348: ?→0

376: 0→?

377: 0→?

378: 2→?

410: ?→1

424: 0→?

428: 2→?

429: 1→?

430: 0→?

434: 2→?

435: 0→?

436: 1→?

438: 1→?

439: 1→?

440: 1→?

441: 1→?

443: 0→?

446: 2→3

449:0→?

450: 1→?

455: ?→1

456: 0→?

459:1→?

460: 0→?

464: 1→?

466: 1→?

467: 1→?

468: 1→?

*Rhabdodon priscus* lectotype and paralectotype dentaries

Scores for non-mandible characters (1–301; 481–944) were set as “?”.

303: ?→0

322: 0→?

323: 1→?

325: 0→?

330: 0→?

341: 0→1

348: 0→1

354: 0→?

363: 1→?

364: 0→?

365: 1→?

366: 1→?

367: 0→?

368: 1→?

369: 0→?

370: 0→?

371: 0→?

372: 0→?

373: 0→?

374: 0→?

375: 0→?

383: 0→?

407: 1→?

408: 1→?

409: 1→?

422: 1→?

423: 0→?

424: 0→?

428: 2→?

429: 1→?

430: 0→?

431: 1→?

433: 1→?

434: 2→?

435: 0→?

436: 1→?

437: 0→?

438: 1→?

439: 1→?

440: 1→?

441: 1→?

442: 0→?

443: 0→?

444: 1→?

445: 0→?

454: 0→?

455: 1→?

456: 0→?

459: 1→?

460: 0→?

466: 1→?

467: 1→?

468: 1→?

*Mochlodon vorosi*

303: ?→0

341: ?→1

347: 1→[01] (intraspecifically variable)

348: 0-→1

455: ?→1

456: ?→0

465: 1→0

*Mochlodon suessi*

303: ?→0

340: ?→1

341: ?→1

348: 0→1

414: ?→2

455: ?→1

456: ?→0

461: 2→1

*Zalmoxes robustus*

327: 2→[12] (some specimens referred to *Z. robustus* have the tip at the mid height).

338: 1→[01]

347: 1→[01] (intraspecifically variable)

348: 0→1

414: ?→2

456: ?→0

*Zalmoxes shqiperorum*

318: 0→? (ventral process not preserved)

328: ?→0

340: ?→1

348: 0→1

414: ?→2

451: 1→0

452: 0→?

456: ?→0

*Convolosaurus marri*

341: 0→1

348: 0→1 (“The posterior end of the tooth row in SMU 70444 and SMU 72834 extends one tooth medial to the coronoid process.” Andrzejewski et al.)

*Iani smithi*

346: 0→1

*Tenontosaurus dossi*

346: 0→1

348: 0→1

*Tenontosaurus tilletii*

346: 0→1

348: 0→1

*Leptoceratops gracilis*

348: 0→1

*Protoceratops andrewsi*

348: 0→1
