# Supplementary material for: Exploring the diversity and disparity of rhabdodontomorph ornithopods from the Late Cretaceous European archipelago
Source: Sci Rep. 2025 Apr 30;15:15209. doi: 10.1038/s41598-025-98083-z (PMC12044058; doi:10.1038/s41598-025-98083-z)
Supplement: Supplementary file 6 — Supplementary Material 6 [file 41598_2025_98083_MOESM6_ESM.docx]

xread

944 176

Euparkeria_capensis 000000010?00000????00000??001?00011000000010000?00000000010000010000000010001000??00002111011000000??????0100111000??1?00000000000100001?00001000000010000000000000011000000010001000000000000?0?000000000000000000?????0102100000000000??????01100001000000000000000100000011000?000000?1?1001?000000000?0000000??????????????00000??0002200000100?000000?????00???0001010000100000?0000000000?000004000000000[2 3]?1?00100?00?000010000000[1 2]000?001?1????0????01?20?00??0000????000???1??00?????????000??0110?01001?0?000100000000????001?0??0010???0?000??0000000000?0000???????0??0????0?????0????00?0?1?01000100010000001??0200002000000000?00?000000000100102110?0000?00?0?????????000011100?1000?00??????????????00010000?0000?0??0?100011?00??1100000002000000000??????0000000000000001001?0021000??0000000000?00000000100000????????0000000?000000000??000?10000000???0100?000?00?0?00001?00???00000?0?000??0??00000000000?0?0?100000000000000000000000?000101000?000000??0?

Gracilisuchus_stipanicicorum 000001000000010????00000??001?000200000???00000?00000000010010000000000010?01000??00012111001001000??????0000110000??0?00100000000000?0101??0000000001010000100000001100000000000102000?1000?0??0000110010101000000?????0100000?0000?000??????0010??000001010??00????20?0????10?0?0????0???100???????10???0000100??????????????000????000220000000??00??00?????01???00010020?010?00000000000??0??000040000001[0 1]03????0?00?0010000?00?000?2000?001??????0????01?10?00??0000????000???1??0??????????001??010100?0?10000001100?0000??2?010?0?00?000??0?000??000?0000??0000??00?000011?????????0?0????00?00??????1?00????????????????????????????????0000000000001?110???00?000?????????????????????????????????????????00000?00?010000??01100011000100010000001010000000??????00000000000000?100??00210011?0000000001?00000000000000????????0000000?000000010??000010000?0????0100?000?0???000001000???00000?0?00???02000000000000?0??010?0?0000000000110???000?000101000?000?00????

Postosuchus_kirkpatricki 000000100000010????00000??001?00020000001010000?0000000000?010001110000100000000??000001100010000010110010102110000??1?00101000100010?01?0010000000001000000000020000100010001010102000000000110?000000000100000000?????0100000100000000??????2?10??00001100000?01?00201000011000?0?0100?200001??0000100000000000??????????????00000??0002021000100?001010?????01???00010000001000000102010000011000040000000101000?0100?0010000100000001000?001??????0????01010?00??0001????000???10000?????????10????????001??00000010?0??00010110100001000000?000?0??0??000010000000100?000001??000?00?0??????00?00??001011210100?0?0000000100?000000000??1001000000000010210112000?100?131000010?0000000001000000000100??????0000010000?010000??00100011010??00[0 1]0010001010000100??????101000000?1?0011000?0?200011?0120000001000000000000000????????0000000?0000000?0??001000000000?0100001110?0010000001000???0?000?0?0?0???????000000000?0??01??1000000000001000?1?00?000111000?000?11?00?

Teleocrater_rhadinus 0??????????0??????????????????????????????00??0?????????0????0?????00?0010001000??00??2?????????????????????010??10????????????????????????????????????????????????????????????1??021010000001?0?00000?????????????????????????????????????????????????????????????????????????????????????????????????????????????????????????????????????????????????????????????????????????????????????????????????????????????????????10000100000?0[1 2]????001??????0????0??????????????????????????????????????0??0010000?001?1100012?1?0000???100???000??0?0???????????0??0?0?0????10???????????0?000?0?????????????10101?200100??0010001010??000000000?????0000000110000111000000??00??????????10?????????????????????????????0?010010?0????????????????????1011000002010000???????????0?????????0?11000?0021000??001?00000?000000000100101100000?00000000?00000?000??000010000000?000000?000?0010?000????????????0?0?00???????????????????????00?0?????0????0??????????0?000??????????????

Lagerpeton_chanarensis 0??????????????????????????????????????????????????????????????????????????????0???????????????????????????????????????????????????????????????????????????????????????????????????????????????????????????????????????????????????????????????????????????????????????????????????????????????????????????????10??????????????00000??0011000101000?00???0?????00???????????????????????????????????0?????????????????00?201002000100000??????????????????????00?00??000110??00???1????????????????????????????????????????????????000?0???0000?????????0000?0?0?00000??00??01010????????????????00?00??11111111010000002000101010110000000?????1000001??0110211???????????????????????????????????????????????????100100???0100????0?100012?00??0011000012000000000??????0000000010000001000?0?00100??00001000?10011000101010?0????????0??0000?0100000?0??000?20010001????[0 1]??1010?01?0?00110000???00100?0?100??03000000010000?0??01000000001001001000?10????0?000??????????????

Ixalerpeton_polesinensis 0???????????????????????????????????????????????????????0????0?????0???0?000??00?????????????0????0????????00000000??1?0??????????????????????????????????????????????????????????????????????????????0?????000??0?????????????????????????????010??0100?01100??0???????????????????1?0???????????????????00???10????????????????00????011000?01?00?0000????????????????????????????????????????????0?????????????????00??01002000100000?000?101???????????01?00?00??000110??00???11?????????????0??????????1101?0???0?1????000??0?00??0??00?00?????????00000000?00000???0??000??????????????????00?00??10102200??00?00020000000??0????????????00?0??1100??1???????????????????????????????????????????????????????00010????010?0???0?100011?00??0010000012000000?00??????000000???0010001000?0?200011?000?1000?10011000101010?0????????000000??0100000?0??0010200??0?10?01001?010?00???00????????????????????????????????????????????????????????????????0??0?000?0????????????

Dimorphodon_macronyx 0100?1110011100?????0010??100?000200000?11000010000?000000?000001110000010?00100??0011?110011010001010011?1?01[0 1]0?00????1??????????000110?1??0000?11001000?1?0000100010000100000012011110000?01?00??????????????????????????????????????????????????????????????????????????????????????0??0?00????????00?00000110??????????????00000???21101010000??00??10?????0????1??1?0?0?0000000??000?100001?0??04210100001?????0100?1210020001?00000000?100??????0????01?03?00??000110??00???11??0????????????????????0???????0100????0???1?00010?000?0000?000??0??1100?0?0??00??0?00??0102????000?0????????010100?1?0010??1?00??001000?11?1?102?00?0???000110100100010010?001?00?00?1030?00??00010000011101?100020011000310?110020?00?011000??0?101[0 1]11000??1??100001?00000000???????0000000?1?010000000?0?01000????0?10?100000?0??1?000000????????0??0?00?000???0?0??000020100000???00????00?0????111000?0???1?11????1?001030000100000000???010?1??00010000000?000?00?00?000?0????????????

Lagosuchus_talampayensis 0?????????????????????????????????????????????0?????????00???00?????0???0?00??????????????????????????????????????????????????????????????????????????????????????????????????????????????????????????????????????????????????????????????????????????000?????0??0?0?200?000?0?00??0100?????????????????????????????????????????????????????????????0???????????????????????????????????????????????0?????????????????00?0010000?00?00?0[1 2]00??001?1????0????0?????????0???????????????????????????100?0010?1000?1000000211000000??10000?00?00100?0???????00000000000000??00?0000101[0 1]0000000100???0?0?00??1?0010??1?00??001000?11?1?102?00?0???1?000?00010[0 1]0?0??0?[0 1]00?00000?0????????????????????????????????????????00011?00?010000??2?100?11000??0101000002001000000??????0010000000000001100?0001000??0000100000000100000010001000000?00000000?1100000?0??000010000[0 1]010?0?000?001?001000001000110110000?0?000110101000001010000?001??0?00010001000?0??1?????0?000?0????????????

Daemonosaurus_chauliodus 0??0??100000010????0000101111?0101?000????100010000?000000?000????000???10001000???00??010001000000???????200010?10??0?1000?0000??100011?0??0000000101100?000??10?00100???00???0??01?0100?0????0?0??2?0?????0?0??0?????????????????????????????????????000????1?0???01????????????????1???0??0????00??0???0?00000??????????????0?00????10000000000??0????0?????0????000??0?00???000??00?000?????????030000000001?1?21100?0110000100000000000?001??????0????01?30?00??0001????00????1??1????????????0??010000?????1??100210???????????????????????????????????????????????????????????????????????????????????????????????????????????????????????????????????????????????????????????????????????????????????????????????????????????????????????????????????????????????????????????????????????????????????????????????????????????????????????????????????????????????????????????????????????????????????????????????????????????????????????????????00?0??????0????????????

Sanjuansaurus_gordilloi 0?????????????????????????????????????????10????????????0????00?0??01000?0001000??00????????????????????????????????????????????????????????????????????????????????????????????????????????????????????????????????????????????????????????????????????????????????????????????????????????????????????????????????????????????????????????????????0???????????????????????????????????????????????0?????????????????00?0?1000010000000?00??001?1????0????01??????????????????????1???0?????????10???010??0?1??0[0 1]?010221011010??11002???000110?????????0?0000?1?1000???0??00?010000?0?????1?????00?00???02100000100??00200001102?102000010?????????????????????1?2?000????????????????????????????????????0??????0??????????????????????????????0?????00??00????000??????101000?0011??????????????????????00010021010000001001??00?00???000000?1100?0000??00000001000100001001001?0???10001101120?11100?0????????????00???????0???1?????????????????????????0?000?0????????????

Staurikosaurus_pricei 0?0???????????????????????????????????????????????????????????????????????????????????????????????????????????????????????????????????????????????????????????????????????????????????????????????????????????????????????????????????????????????????????????????????????????????????????????????????????????00???????????????00000??0000?00100000?00?0?0?????00???000?001000??0?00??0000????0??00?0?????????????????00?001000010000000??????????????????????10?00???000????00???????????????????0?????????010101000022?1110????1?000?00000010??????0??001000010100000?0??000?00?100010000??????00?00?????????0???????????????????????????????????????????????????????????????????????????????????????????????????00000?00?010000??1?100011001000101020012001000100??????101??000011?00?11?0??0????1101?1?10010021010000001001110000010100000?1?100000?0??00000000000100001000001?0100000?1????????????????????????????????????????????????????????????????0??000??????????????

Gnathovorax_cabreirai 0000?0010000010????000?101011?010210000???10000?000?00?0000000121000020000001000??000?2010011010000?????????0000110????10??0000?0?10000001??0000000001?0000?0000000010000001000?000100101000?0001000??0000100000000?????000?100?0??01000??????10001?21000101000??0?00001010011000?00?110??????????????????0000000??????????????0000???0000000000000?00?1?0?????0????000101000011000000000000000100??030000000001?1?20100?0010000100000003000?001?1????0????01020?00??0000????00????10000?????????10000010000010101000022111[0 1]0000021001?0?1000?00?0?0?0??000?0?01?100000100?000001010[0 1]010010?0???100?000?00210000000001002000211011000000?00001210011001010000111112000000??01?010?001100001002111?11000001000011??100000?00?010000??11100011000000001020002010000100??????00100000001000011000002100110221?00010020010000210000110000010100000001100100?0??00000000000200001000001?010000001??1110111100?0?010?0010?000001010000?0010?10100100000010?000000?00?000?0????????????

Herrerasaurus_ischigualastensis 000000010000010????0000101011?0102100000??10000?00010000010000000000100100001000??00000110112000000??????0200000110??0?1000000000010011000??000000000110000?000000001000000100000102001000?0?0001000010000100000000?????0000000100000000??????1010??210?000000?00??0010?00?0110?000011?0??01000?000001?00?0000000??????????????00000???00000000000??00??10?????00???000100100011000000000000?00?000004000000000?????0100?0010000100000003000?001?1????0????01?10?00???000????0?????1??00?????????20000010000?10?0100002211110100?11002?000001100???000??00000?01010000010?00001020100010001?0????00?00???02101000100??00101020002?[0 1]0?0?00????120??11001?11?101001020000?00001101100011000010021100110000010000110?10?000000?000000??121000010010001[0 1]1020012001000100??????10100000011?00011000000100110221000010021010000001001110000010100000011100100?0??00000000100200001001001?01?000001?01110211100?0?000?00201100001010000?00100101101000000000000000?00?000?0????????????

Buriolestes_schultzi 000000100???110????000?101110?10012?000???1000110000000000?010000000000010001000??0001?110101000000??????0100100110??0?10100000000100010001?0000000001100000000000001100000100000[0 1]0100100000?0000000000000100000000?????0000000100011000??????00100001000000000000100000001011001000?100??00001?00000000000000000??????????????00000??0100010100000?000000?????0????00000000001000000002000100010010040000001010?1?00100?0010000100000003000?001?1????0????01?00?00??0001????00????1??0??????????1000101110010010110002211110101021010?001001000?0???0??001000010100000?00??0101?01000?000???????00?00??0??0????010??????0001?001?0????0??0??1?00011001111010?010?0?00?1010????????????????????????????????????????00000000?0[0 1]0000??1?100111001100011110012011000100??????10100000021000001000?00000110212?10010020010000001011110010010100000001100100?0??00000000001200000001001?000010001??11?????1?????000?00??1?000010100?0?00100101001100000000001000?00?000?0????????????

Eoraptor_lunensis 000000100001010????0000100011?110120000?0010000?0000000000?010000000000010001000??0001?111101000000??????0000100110??0?101?0000?0010000000??0000000001100000000100001100000000000100??00000?010??0000?000000?000000?????0000100000010000??????????????????00???00?????????????0?000??1?0???10010?00000000?0000000??????????????00000???00000010000??000000?????00???0001000001100000000200000001000004000101000100000100?01100011000000030000001?1????0????0??[0 1]??00??0000????00?0?????00?????????100?001??00000?01100022?1010101011000?00100000?00?0?0??01000001??0000?100?10101?01000100?110????00?00??001001000000?100200011[0 1]01?00200??0???1110011001011011101000000010100[0 1]00??000110001110201001000000100001?0?100000?00?0?0000??22?000?1?011000111200?2011010100??????101000000[0 2]000001[1 2]0000000000??22201001?02?0?0?0000?0??11?00?010100???001100100?0??00000000100101?00001001?0??000?01001110211100?0?000000201100001010000?00100101101100000100001000?00?000?0????????????

Bagualosaurus_agudoensis 0010????0???010????00??10111??100110010???10000?????????0??0100??100?00010?0?0?0???00????011100?0????????????????????????00?0000??1?000000???00?000?01000000?00????0??0?00????????????????????????????????????????????????????????????????????????????????????????????????????????????????0??????????????00?0??00??????????????00000??0110000100000??0???100?000100?000?000000?0?0??0???000?????????040100011013????0100?01100112000000?30000101??0???0????0??00?00??00??0??????0?1???0???????????????????????????????????????????101??00?00?000???0????001?0001010?00?1???0??????????1?????0????0??????????????????????????????????????????????????????????????????????????????????????????????????????????????????0000?00?0???????00100?11001100011110011011000?00???????01?0??000???????????????????????0001??200???00??????1100000101??????11100100?0???000??????010?000001?01??000000?????????????????00????????00?01010000?0010?0?110110010?1???01????0??000??????????????

Plateosaurus_trossingensis 001000010011010????0001111110?1[0 1]0[1 2]1001001010000?0100000000?010000010000010101000??00002110111010000??????0201000[0 1]10??0?1010100000010000000110000[0 1]0000[0 1][0 1]00000000010001[0 1]0000000000[0 1]10[1 2]0010100001000000010010101000000?????0000000000011000??????201000210000010000002000000010010010001000?201001000000100000000000??????????????00100??0110101000100?10011100?00000000[0 1][0 1]0000000100000000[1 2]0001010100100[5 6]010001001100220100?11100212000000030000100?20???0????01000?00??000000??00?0?0100000001010002000001010011010110002211111101011010000100100?200000??00100001010000010001010120[0 1]0001000?00???100?001000102[1 2]2001000[0 1]00200011101?00[1 2]000?000012[0 1]001100111100010000000101110030?00?00110011110210001000000000003131100010000?000010??0?100011011[1 3]00101020001011010100??????1010000000100001200010210011?21200001002001000001100111000001010000001010010000??0000[0 1]0[0 1]0[0 1]0020[0 1]001001001?000000001101120211100?0?101010201100000000000?00100001101000100100000000?00?000?0????????????

Eodromaeus_murphi 000???0????1010????0???1010?1???0110000???00000??0???0?00??0?00?0?00100000?00000???01????????0???0????????????????????????????????????0000???00????????0??????0??0?0????0?01?000?00110000??0?0?000???????????????0????????????????????????????????????????????????????0????01?000?00?110??0??????????0???00?0?000??????????????00000???000000?0000??0?0????????00???????000????00?0000010000?????0??04000000000100000100?0010000100000000001?001?1????0????01?20?00??0000????00????1??0??????????20???00?0?011010100102211?1?10?011010?00000000??00??0??0100?0????????0100?10000???0000000100???000?00??00100000??000000200011102?00?0?00?0??1[1 2]100?1?011110102??1?2?00?1???011?11000?1000011???1001000000010001111100000?00?00000???1?100111001100101020012011000100??????101?00000210???????0??2?001101?1?100100210?000000?0?11100000101??00000110?1?0?0??0000?0???0?1????1???001??1?0?0001?011102?1??0?0?00????????0000101?0????01?????1???000?01??0????0?00?000?0????????????

Tawa_hallae 0000??100011100????0000100100?100110000?0??01010000000?000?00000001000?100001000??001??110012000000??????????????1?????1000000001?10011000??000000000000000000010000100?00010000010110000000?0?000?????????0??????0?????00????0100001000??????????0???0?0?000?0??000??0???00??000000111???????????????????0000000??????????????00000??010000000000??00?0?0??????0???000?001000?0000000000000????0???03???????????????100?0010000100000001000?001?1????0????01?00?00???000????00????1???0?????????????????1??111????010221021?1????1??0????????0?????????0??????????????10??0????????00000????????00?00??01210000??0001002000111???0??0?00??00121101?0011010100?0000?00?00000001?10000100001002111?100000011000210?100010?0??0?0000??0?1000010010001010200?2011000100??????101100?00?1??01?20???????????????100100211101100010101100100101??0?00001000?010???000?0??00020???10??001?1100?0001101110211100?0?0[0 1]0100??110000?010000?001??100?010??0?0?????1?00?0??000?0????????????

Coelophysis_bauri 000000100001000????0000100100?0001000000110000110000000000?010000000000110001000??0011211[0 1]10[1 2]0[0 1][0 1][0 1]00??????0201010110??1?100000000001000000[0 1]??000000000000000000010000110000000000010110000000?000?0?0010000100000000?????0000001100001000??????1110??112?1100000???????0?0?1011000?001110?200000??0000101000000000??????????????00000??0100000100000??00000?????00???000?0[0 1]0000000000000100[0 1]0?????01?040001000[0 1]0[2 3]?1?20100?0010000100000003000?001?1????0????01000?00??0000????00????1??[0 1]00000000002000001011011110100112211211100001010?000001001000000??11100[0 1]110?01000100010[0 1]0121100000001?0???000?00111010[1 2][0 1][1 2]0000001002000011111002000000??12110100011010[0 1]00??00[0 1]?00?10011[2 3]0?0100001000011021100110000001000110?110000?00?000000??00100111001100001021011011010100??????00100000000[0 1]1011200100000011?212010000021110000??20111100000101??0000111000?010??0000101111120?00100?0?1?1??0?01111011102?1110?0?110??0001001001012000?0010000010110010010?0?1000?00?000?0????????????

Dilophosaurus_wetherilli 0000??100001010????000?0??100?1000000000110000110?00000000?000000000100100011100??001??100102010000??????02?10?0?10??0?1001000010?10011000??000000000010000000010000000000010000??01?000000000?000100?00?0101000?00?????0?0???0100000000???????1100101100100000?002001000010100001001100?20??????000?1???00000000??????????????00000??0100000100000?0000?10??0???0??0?????000?010000000100100101001?0400110000011[0 2]110100?0010000100000002000?001?1????0????01010?00??0000????00????10?0??????????20001011[0 1]001111111121211121000000101000?100101?000000??1110?00101000001000100012?100000000?0???000?00??00102[1 2]100100??002000011011002000000??1210001001111010211111000?1001???????001101001102111?110010000000100?110000000?000000??00100111011100001021002011010100??????00100000020?000?21???????011?212?0001002111010100201111000001210000001110000010??1000001011120001100101201010?0001101110210110?0?110??0201100001012100?00100100101100100000001000?00?000?0????????????

Lewisuchus_admixtus 0??00???0?????0????00??1?011???001?0000000?0??0??????0??0??0?0000?0000001000?0?0???0??2??????0??0???????????????????????01[0 1]0000200000?0000?1000000000110000000100000110000000000000210[0 1]00000?00000000?0????0?????00???????00?00000000100??????1010??01000100001???000000?00011000010101?????000?00???001??0000?00??????????????00000??0??0000100100?0001??????????????????10?0000?000002000000??10??1[4 5]00?1001103????0100?0010000100000003000?001?1????0????01000??0??0001????00????10000?????????1000001?110?001010000[0 2]211000000?00010000000100?0?0000??0000??0001?000?100?100021?00??0?0?1?0????00?00???01001201?000100100011[0 1]01110200??0??????00?000101???01001?00000??00?????????0??????????????????????????????00000?00?000000??001000110011000010?000?001000100???????01000?0020000?0100??12?000??0???10000011010100001001110000010100000011100000?0??00?00001010[1 2]00000001001?0??010?010011101[0 1]0000?0?00????????????????????????????????????????????0??000001000?000??????0

Asilisaurus_kongwe 0?????1??0?0100????00?0101011?00011001000??00?0?000000?00????000?000?0010000?0?0??0???2?101120?000???????01?0100110??1??????????????0?1000010100????????0???0??????0??0?00???000?210000000?0?0?000001?0????0??????0?????000???0000001?00??????????????????????????????????????????????????????0?????0?01??0000?10??????????????0?0?0??0210000000?00?0000?0?????0????0?????0001100000000100?0000?10??11???????????????100??001021[0 1]000000000????????????????????30?00??000000??00?0?1?0?????????????00?00100?0100100100022?100000???1010?000?00000????????0??00000010000010??100?11000??10001?????????00??001012200100??0020001110010???00000??0?0000100101000021010100000000?????????00?????????????????????????????10000001?010000??10100011001100002000011011000100??????001000?0020?00??100??1????0???12?0000001101010000101111000001010000001110000110??01001000000100000001001?001000001001110110000?0?000??000000000001000100010000000000???00?0??100??0??000?0????????????

Diodorus_scytobrachion 0????????????????????????????????????????????????????????????????????????????????????????????????????????????????????????????????????????????????????????????????????????????????????????????????????????????????????????????????????????????????????????????????????????????????????????????????????????????????????????????????10??????000?00000???001????????????????????????????????????????????1?????????????????00??00102120000000???????????????????????0?00??0?1100??00?0?1???????????????????????????????????????????????????????????????????????????????????????????????????????????????????????????????????????????????????????????????00000000010111????????????????????????????????????????????????????????????????????????????????????????????????????????????????????????????????????????????0000011001100101010110010010101?0000?1000?1?0??010020100????????????????????????????????????????????????????????????????????????????????????????????????????????????

Eucoelophysis_baldwini 0????????????????????????????????????????????????????????????????????????????????????????????????????????????????????????????????????????????????????????????????????????????????????????????????????????????????????????????????????????????????????????????????????????????????????????????????????????????????????????????????1???????000?000?0??1??1????????????????????????????????????????????1??????????????????????01021??0???11?????????????????????????????0???00??0??????????????????????????????????????????????????????????????????????????????????????????????????????????????????????????????????01???????0002010??1????????????????????????????????????????????????????????????????????????????????0?010??0?01??00??1?1??011??11?0002??00??011????00??????1010000?02??00???????????????0????000001100010000101012101001111?00000010000010??0?0?00?0??01?000?000??????????????????????????????????????0???1?????????1????????????????????????????????????????????

Gamatavus_antiquus 0???????????????????????????????????????????????????????????????????????????????????????????????????????????????????????????????????????????????????????????????????????????????????????????????????????????????????????????????????????????????????????????????????????????????????????????????????????????????????????????????????????????????????????????????????????????????????????????????????????????????????????????????????????????????????????????????????????????????????????????????????????????????????????????????????????????????????????????????????????????000010?0??????1???????????????????????????????????????????????????????????????????????????????????????????????????????????????????????????????????0?????10100?01001100002000001011000??????????????????????????????????????????????001100110000?0101100000101000000?01000???????????????????????????????????????????????????????????????????????????????????????????????????????????????????????????

Kwanasaurus_williamparkeri 0???????????????????????????????????????????????????????0????0????????000000?0?0??????2????????????????????????????????????????????????????????????????????????????????????????????????????????????????????????????????????????????????????????????????????????????????????????????????????????????????????????10??????????????0110???0010000000000?10?1?0?????01???0???????????????????0???????????1????????????????100?10010212000001111010100000???0????00?20?00??001100??00?0?10?????????????????????????????????????????????????????????????????????????????????????????????????????????????????????01?0?0?01???????0002???????????????????0000000000000101????????????????????????????????????????????????????0010000?010000??12100011011100012020002011000???????????????????????????????????????????000001?00110010001012100001110000000[0 1]1000??10??0100010000111000?001011?0????????????????????????????????????????????????????????????????????????????????????????????

Lutungutali_sitwensis 0???????????????????????????????????????????????????????0????0?????????0??????00????????????????????????????????????????????????????????????????????????????????????????????????????????????????????????????????????????????????????????????????????????????????????????????????????????????????????????????????????????????????????????????????????????????????????????????????????????????????????1???????????????????????????????????????????????????????????????????????????????????????????????????????????????????????????????????????????????????????????????????????00?11??????????????????????????????????????????????????????????????????????????????????????????????????????????????????????????????????0?0000???011000??1?100011?0110001200000101100???????????????????2000?11100??1????0??0??????00011001100?0001011???0??0????0?0?0100001?????1?????????????????????????????0?001110?10010?0?10????2010??????????10???10?00???????????????????????????????????????

Sacisaurus_agudoensis 0??????????????????????????????????????????0??0?????????0????01?0??0100000001000??00??2??????0??????????????????????????010000000??????????????????????????????????0???????????????????????????????????????0????????????????????????????????????????????????????????????0?????????????????????????????????00???10??????????????00100??00[0 1]000000000??1001???0????????????????????????????0????????0??1????????????????100?100102120000011010?01000?00??0????000?0?000?001101000000?00?0??????????????0??????????????????????????????????????????????????????????????????????10?011100?0?0?0???????????????0210210??00?1002001111???0???????0????????????????????????????????????????????????????????????????????????0????????0???????????????0?10000020000??011???100??????1010000002???0??100??1????11?0?1?100000110011000010101100000111[0 1]0000000100001?0??010000001011?0001001011?1?????0???????????????????????????????????????????0???????0???0??????0???????????????????????

Silesaurus_opolensis 000000???0??100????00??101011?000110000000?0000?000000000????0????0?10010000?0?0?????02??????0??00???????0?0010?100??????0??0????0000?1000??0000?00?01??00000?00??00?101?????0??0??000000000?0?00000000????00?????0?????????????0??1??00??????1010??01001000001?010000000000110000000110??01000?0000?100??000?010??????????????00000??0010000010000?00?1?0?????00???001?0?0000?0000000010000??????0?150?0????????????100?100102120000011000101010?0???0????00?20?000?001000??00?0?100??0??????????000001001011110110002210110000021000?000000000000??0??120000?0010000010000000110000000??100????00?00??00210210011000002000211011[0 1]0201000???0000000001000010101000000?0000?????????0??????????????????????????????00000011?010000??1?1000110011000020000?1011000100??????101000000?000001100?0021000??00110000001100110000101011000100010000000010000010??0100000000111000100?0[0 1][1 2]?111000001000110110100?0?00????????0000101???0?0011000110100???000?0?1000?00?000?0????????????

Technosaurus_smalli 0?????????????0????00??1?101??00?1?000?????000???????????????????????????????????????????????????????????????????????????????????????????????????????????????????????????????????????????????????????????????????????????????????????????????????????????????????????????????????????????????????????????????????????????????????0????0??000?000000?000??0?????01???0???????????????????????????????15???011??13????0?00?100102120000011???????????????????????0??0??001?00??00?0?1???0?????????????????????????????????????????????????????????????????????????????????????????????????????????????????????????????????????????????????????????????????????????????????????????????????????????????????????????????????????????????????????????????????????????????????????????????????????????????????????????????????????????????????????????????????????????????????????????????????????????????????????????????????????????????????????????????????????????????????????????

Pisanosaurus_mertii 0??????????????????????????????????????????????????????????????????????????????11?????????????????????????????????????????????????????????????????????????????????????????????????????????????????????????????????????????????????????????????????????????????????????????????????????????????????????????????0????????????????0010???0?100000?0000?000?010010101000???0000000000001000200000?1?00001?????????????????00?10011210?000?112?0101?00000??0????00?10?010?0110010??000?100??0???????????????????????0????00?1???????????000?000?000??????????1??????0??????????????????????????????????????????????????00?????????1??????????????????????????????????????????????????????0????????????????????????????????????????????????????????????0?????00??0??000?00???????01?00?00???000?1??????????????????0?????????????????????????????????????????00??010000?0001100000011001?0?100000100111001?100?0?01????20100???1???????00100??11???0???000000?0???0???????????????????

Abrictosaurus_consors 0000?0??0??0?00????????1?0011?000120?1??0??00?11????????00?10001010??0101??0?0?1100?0??????1?00???10000?????????????0???????????????????0???????????????0???????2???1???0??0?00??0???????0????????????????????????????????????????????????????????????????????????????????????????????????????????????????000??01010?000???0???0110000??000?100010???001?1001010?00?0????0?0???000???00?0????????0?0030001001013?????101?121102120010001210111100?1?000????00?10?110??01010???0?0?00??01??????????0????????????0?????????0???0??????????????????????????111??0?0??00?????????????????????????????10?0???0??????????????????????????????????????0????00?01101?????????0??0??030?10010110?0001021?00?1000?0???????11?0?02??00?00101???1?0011110011?1??1?210?2000000??????????????????????????????????????????100???2?????????????121?10102???0????02101?0?0?????0????????????????112?1????1001??????0???1??????0???????0???10110???001??0??1??1001001010?1000000?000?0????????????

Fruitadens_haagarorum 0?????????????0????00????0?1????0??001?1????0?11????????0??11?0????0??1?1000???10000??0????????????????????????????????????????????????????1??????????????????????????????????????????????????????????????????????????????????????????????????????????????????????????????????????????????????0?????0???????????1??????????0???0110000001000?010?00?1001?10010101?0?????????????????????0???????????03000001001????00100?120102120000011000?01000?1?000????10?210110?000000???0?0?00??00??????????0????????????????00011?1?0??0???00???00?1??0??????????221???????00???????00001???????0000??????????0?????????????????????????????????????????01011001?01011101???????????????????????????????????????????????????????????????????????????????????????????????????????????????????????????????????????????100100200100?0101000121111101???0110102101?0?0??00000?0100120100011?11221010?111??11130?1?111?0111?????????????01??0??0?1?????????0??0???????????????????????????????

Echinodon_becklesii 0?????????????0????????1??0????00??0?1?1??????11???????????????????????0?00??0?10???0??????????????????????????????????????????????????????????????????????????????????????????????????????????????????????????????????????????????????????????????????????????????????????????????????????????????????????????????????????????0?10000??10??0010000?10?1???010????0?????????????????????????????????03000101?113?????10001201021200?0011?00101000?21100????10?[2 3]10100?010000?????0?00??00????????????????????????????????????????????????????????????????????????????????????????????????????????????????????????????????????????????????????????????????????????????????????????????????????????????????????????????????????????????????????????????????????????????????????????????????????????????????????????????????????????????????????????????????????????????????????????????????????????????????????????????????????????????????????????????????????????????????????????

Heterodontosaurus_tucki 000000100000100????00101000130010120?1110?0000110001000001010001011010101000000110000020110110000010000111100001110?00?10001001100002110000?200000000010000000002000110101100000000010010010?0000000220000001010000?????0000000100000000??????2100000100111000000000000?0010111000001000??00000000?0011??00000001010100000?0???01100000000021000000?1011?100101000000011002001100000010200010001000003200001[0 1]012?1??1101012111202001000001111010??2110001??11?211010?001011000000?110001?????????10?0?11001000000000001001000001000002?00100000?000010??22100000?0000?01000000010010000000010???11000011012012010?000000101020102?10100?00?001201011001111010?01112000010?1000011100110000110210001100000010003020101021?000000000??1?00010100110[0 1]100021012000000111000002101000010100000120110000000??011?10010?2001?00?10??0012111110110001?0002101?0?0??00000?0000120?10011?102?1??1?1111?1?13001?10????111?1121?00111101100??001??0?01011001001010?1000?00?000?0????????????

Lycorhinus_angustidens 0?????????????0????????1??0????001??01?????00?110???????00?1?0??????00101????0?110?0??????????????????????????????????????????????????????????????????????????????????????????????????????????????????????????????????????????????????????????????????????????????????????????????????????????????????????????0??0?????????????011000000100000?010??00????0?????000?0????????1?0?????0??0????????0??032001011002?1?0110101211021200100012?0111000?1??00????10?211110?00101??0?0?0?00??01????????????????????????????????????????????????????????????????????????????????????????????????????????????????????????????????????????????????????????????????????????????????????????????????????????????????????????????????????????????????????????????????????????????????????????????????????????????????????????????????????????????????????????????????????????????????????????????????????????????????????????????????????????????????????????????????????????????????????????

Manidens_condorensis 00?000111??0?????????001????1????????????110??11?00110?0010100010000?000100000010000?021111110011010000111101001110?00?100110011000?20100001210000??0?1?000000?0200001010110000001001011001011001?0021000100101???0?????0000?00100000000??????2??0?001001100001010000010001011100000010????0000000??010???00000????????????????0100???0??000?001000?00011101001000001??010100110000000020000000100000????????????????100012110[1 2]1200[0 1]001101?111100?1?000????1002111110001010000000?0000?[0 1]0011010[0 1]0??????????0000?00?000?0?1?0??0????000?00??0000?????????2??00???????0??????0????????????????????????????????????????0????????????????????0?????????????????????????????????????????????????????????????????????????0?021?00?00?000??1?000101?111010?002101??1?000?110?????1?1000?1?1??000120000????????0??????????????????????????????????????????????????????????????????????????????????????????????????????????????????????????????????????????????????0?0????0??????????????

Pegomastax_africana 0????????????????????????????????????????????????????????????????????????????????????????????0??????????????????????????00???????0??????????????0?????????00??????????????????????????????????????????????????????????????????????????0???????????????????????????????????????????????????????????????????????0010?010000??1?0?0110000000012100000??00?1?1?00010????1???????????????????0???????????0????????????????1011121101120000001????????????????????0?2110110001011000000?10????????????????????????????????????????????????????????????????????????????????????????????????????????????????????????????????????????????????????????????????????????????????????????????????????????????????????????????????????????????????????????????????????????????????????????????????????????????????????????????????????????????????????????????????????????????????????????????????????????????????????????????????????????????????????????????????????????????????????????????

Tianyulong_confuciusi 0000??100000?00??????001000?20000120?1??0?0000110001?0?000?100?1011?00101?00???100?00??00101??000?????0?????????????0???????0????????1?00???000???0?????0???????????1?????????????0?????????????????2?????????????????????????????????????????????????????????????????????????????????????????????????????????101010?000???0???0110?0???10??100000???????1000010100?0???0020?000000000??0010????????02210?001012?1???100?220102120000011010101000?2?100????10??10010??0000????0?0?10??00???????????????????0???????????????????????0???????0?0?????0?????1?????????????10??00?0??0??00?0?00?0???111010??????1?00???000??????????????2??0?0???1201?1?00?00??10?0???2?00??0?1??0?1???0111000?10?1???110000001000?0?01??????????????0????????????????????????????????1?????????????1101???????????1?0??0??001?100???2???0????????????????01????????????1???1??00?0??????1????0????112????0?111???????????1?????????1????0111100?00??0?1??0???01?0010010?0??1???00?000?0????????????

Eocursor_parvus 00????????????????????????????????????????????????????????????????????????????????????????????????????????????????0??????????????0????????????????????????????????????????????????????????????????????0??0??1000100??????????????????????????????????1?????????????????????0????0000101???????????????????????1?1??????????????01101??00?00100?000??1001?0?0201010??001??0200??1000000010001000?00?00?????????????????00?1?01021200000?1?????10???????????????????1??0??000???0?0?0??0????????????0????????????000?0001???00??0???00??00?100?0??????????111??????0?????????0????????????????????????????0021?1000000?10020000101??0????????????0?00000101111120??????????????????????1????????????1?????0?1????????0??20000?0000?0??1??00001011100001020000000000?110?00001010000??1??0000200??1?00?0??0010100200200101000010001210100021000001002001?0?0??01000?000?120010011?11221??0?00???????????????????????????000?1???????101?0???1???0??????????????0??000?0????????????

Laquintasaura_venezuelae 0??????????0??0????010?1?0011?0001?001?1?00000??????????00?1??010?0?0???????00?0??????????0???0?0???????????????????????0000?00?1?0?0???????0?0000??0??0000?00?????0???????????????0?0?0?0?????00?0?0?0?????????????????????????0?????0????????????????????????????????????0????????????????????????????????????????????????????????????????????????1???????????????????????????????????????????????060?000????????????0???1102120000111????0100000??010100?0???????????000???0?0?00???0??????????????00001010??0??0??11?1????????000??00??0100?????????????????????0??10??0?0??0???0000??0??????????????1102?100?00?10?100011????0????????????????????????0???????????????????????????????????????????????????????0??2??00?000000??????0?01?01100??1?200?0000000?110?0???1?1?000??1??0??0200??1???12100??0?0?200200?1?10[0 1][0 1]1000121?100021?????0001001???0??000010101001????0????1221??????11??1???0????1?0?????????????????????????1???????????????????????????000??????????????

Lesothosaurus_diagnosticus 0000001000?0100????0100100011?0001100101?00000100001000000?1[0 1]00101010000100000010?100000100110000010000100000000110?00?10001000000000000001?000010000010000000001000100001000000000000010000?000000000000000[0 1]000100?????0000000000000000??????1001000100110000000000000010?010001001?0?0?1010000000001010000000010001000000100?00101000000010010100?101100?0001000?00011001000110000000200010101000006000001000220020101?200102120000011[0 1]1010100001?000????00000?010?001000???0?0?000000???????????0?00100100?0000?000110100??0???0000?00100[0 1]00??????????11?0100?00?00??00?00?0??000??100?0?0????100000?001012100000010020000110??021000000??2110010001001001200101?0101100???0?????0?0000000110001001?00100?????0000020000?000000??1?000001011000111020010000000111000001101[0 1]000111010001200101100020[0 1]001[0 1]1001002001001000100012101000210000?00020010000??0000000000120??000?011221??00000100111001010100?11010??11?0000101100??101?01?011??0010000?0??000?00?000?0????????????

Scutellosaurus_lawleri 00?0?01?000?200????01??10001???0011001010??00????00000?000?1000101100000?0?012?100?00??00?01100?10???????01?0100010??0?10001000000000000010?000000000000000000000000100101000000?00110[0 1]00000?01000?0010??0?01?00??0???????0???0?00000000????????????010???00????0???000000????????????????????????????????100???1?????0????????00100000010000000000?1011?0???0101????????0?000?10000?0020???????????060?0???001??????100?100102120001011?1010100001?100????10?00?010?011000??00?0?000000?????????10000?000???00000000011??00??0101001?00000?10?0?00??0??110?0???0?????1100?0001110?00010000?0???????00??100012011?00?0001000011012021000000????00010001010111200000?010100??????????00????????0???1??0????????????00?120000?010100??1?000001011000110020010010000111??0?01101100010?0?000120010100?00??001010020020000110[0 1]0110[0 1]12101000210000000020110000??00000001000[1 2]0010001?11201010?0001111120010101?0111010?11000000000100??001?0?1010??00?0010?00?00??000112?00??00?11??00

Emausaurus_ernstii 0??0001000?0200??????0??000?1?0?0?10??01??00?00??????00?01010001011000001000?101100000201001?000?01001010010?0?0110?00?110000000000020001????000100?0110000?00001?0010010100100???0??????0???0????????00?0?0???0?00?????0000?001?001??00??????1?????????????????????0??????0???00?????????????????????????1000????0???0????????0?00100?1111?0000001010?1?0?0101?10??000??0?0001100?0?00200210???????05000001001020221100?1001021200010113??101000?1?100????0??00?010?010000???0???0???10???????????????????????????????????????10????????????????????????????????????????0?0????????0?1?0???????????????????????????????????????????????????????????????????????????????????????????0????????????????????????????????????????????????????????????????????????????????????????????????????????????????????????????????????????????????????????????????????????????????????????????????????????????????????????????????????????????????????????????0??????000?0?0112?0????0???????

Yuxisaurus_kopchicki 0???0?????????????????????????????????????????10????????0101?00100000000100?110110??01000000?1?0?01[1 2]1?001?????????0?0???0??????????????01???????????????????0??????0???????????????0?011?000???010?1??0?00?01010000?????00000001000???00???????1000?01?011000020?0000?0?00100?211?01?00?????01?????????????0?0???????????????????????????????????????????????????00?1?????1000100000000200??110?0???0?????????????????0??1?010?1200001??21??010???0???1010??0??????????????????????0???????????????1?0010010?000001000100001??????00?000?20??0?1????????????????????????????????????????????????????????????1?0100???????0000011????????????????1010000111001100???????????????????????????????????????????????????????????????????????????????????????????????????????????????????????????????????????????????????????????????????????????????????????010000?010101?????????????????????????????????????????????????????????????????????????????????????0??0011120120??10??????

Scelidosaurus_harrisonii 000000100?00100????0100101011?00011001010000000?00000000010100020100100010001001100000000000100010121?0011102011100?00?1101100000000200010010000[1 2]00000100001000110001001010011000000101000000100000[0 1]000000001000000?????000000010001?000??????11001001001100002000000000011001211001101100000000011101010010000?1?0???0????????01101??00010000?01011101100?1101010?01??10010011110000002001011010000050001010010[1 2][0 2][0 2]00100010010212000001131110100011?000????10000?010?001000??00?0?000?00?????????0100001000000000010201010010001020001101100[0 1]001000101??1100000000000101001001011000001000010????10000100010020000000000110001[0 1]01002000000??0110?010000011111100000001?100002?01??00010000011200001001?00100003121001020000?001100??12000001011000110110012000000111000001100110011101001020000001000??001100020020000?10001100121010002100010010201101?1??000010011002011000111122101010001011120010101000110100110000000001000?00000100101110000000001011010[0 1]11201212000120100

Jakapil_kaniukura 0?0????????0????????1??1?0?1??0?011001????000010????????1????1???????????0?0??01?0?0????????????????????????????????????????????????????????????????????????????????????????????????????????????????00????????????????????????????????????????????????????????????????????????2?1?1???????????????????????????0010?00000001110?0110100011000001001??1????101?01010??1??101?000100?1000010010110100?000?????????????????00?001011200010?0???10101?10???0????10020?010?00?000??00?0?00??????????????????????????????????????????0???00???000???0???00???????1???????????????????????????10??????????????????1111001?00?001?00001101????????????????010??0?????????0?0?????????????????00????????????????????????????0???????????????????????????????????????????????????????????????????0??0?????00??00??0?01????????????????????12??1???1????????0????0????????0?????????????????122?110??????????????????????????????????????????????????????1???10?????????0?0112?1????????????

Isaberrysaura_mollensis 0??0?01?0?????????????0?????1?0??1100?????1?000??00100?0010?00010110000010??10?110000??000?100?1101[1 2]1?001???2???10???0?100??000001000100?1??000000010??0000?0?1???0000000100000?0?????????????????????0?000??????????????????0???????1????????1???????????????????????????????????????????????????????????0?????1?0??????????????????????0110???????????????????????????????????????????????????????0?0101010113??????10???010212000001?311?0100110???0????1?0????1???????????????????1??????????????????????????????????????????1???????????????????????????????????????????????????????????????????????????????????????????????????????????????????????????????????????????????????????????????????????????????????????????????????????????????????????????????????????????????????????????????????????????????????????????????????????????????????????????????????????????????????????????????????????????????????????????????????????????????????????0??????????????????????

Huayangosaurus_taibaii 001000010000100????0?00001011?010120010???10000?0001000000?100010000000010?00001100001?00101100010121?001?11201011??0??110?000000000200010??000020000100000000001000100001001000000110100100?0000010010000001000100?????0100000000000100??????1100??0?00111000?0???00100?0?0?121??????????01000?00?001????0000001000?0000?0100?0110000010010100010???0???0?01110100?001100100000000000000021????00??06000101001120???111?100102120000011311101?0011?1?0????1?000?010?000000??1??0?0????0?????????1?1?00000?0000000100020100?0001010112?01?0100010?010100110????00?00011?01??1?10000000?0000??????1?0?0??001000011?00?0001100111???1?000000?????0?01010?01100110??1?????????????????????????????????????????????????0?120010?011100??2200010100100011012000100000111110000011011001?11??0????????????????????1120120?1??10????001220100?01??0??0?0001001????001????00?????????????????????????????????????????????????1???0?????0??1???10?1???2???1?1?1???00?000111?00?000?11?00?

Stegosaurus_stenops 001000110011010????0000100011?00011001?10?0000100000000000??00010000000010?10101100011200100100010121?001?2?2011100?0??100?100000000200010??0000100100000000001110000000010010001000101001?0?0000010000000001000100?????0100000000000100??????110?1?01001110000000000100?01001210?01???1000100000010011?00000000100001000?0100?01101000110101000001?101?10?0?11000??001000000000000000020001110?000000???????????????011?10011212000011131110100010???101000?000?010?0??000???0?0?0?00?[0 1]0001100102110000000010010010001[0 1]01001001010112?01001010[0 1]000100??[1 2]1[0 1]001?1[0 1]?0001?101?1111010000010100?0???00??00??00100000100000002100[1 2]1102?12000010???001001010001100110011200101111141000110101?11011000010?1110?1?1?11100?01120010?001100??22000101001000110120002010000111100001110110011011000020010001010??0001001200201100111010001220100101000000?0001001?100000020110001001000011122?00010[0 1]01111100?1?100?0?11????????10000[0 1]1???0?1100000001??2???10?0???000?000111000?000?11?000

Kunbarrasaurus_ieversi 0000010?1?00???????????1????1?0?????????0?0000???00000?01????1???????????0?01101100000010001100000101?0011102011??0?01?110?0100001002??1100?0000?000010?010000010001????0100?1000001???001???01??1??211???0?1000100?????01001?0??01??0?0??????0100??0100111000?000??000?0??00020????1??112000000?000010??01011???????????????????1????1??11?00?0?0???????0?0?010?0??????????????????????????????????00?????????????????????0102120000111????01000?0???0?????00?????????????????????0???0???????????????????????????????????????????0????1??0???????1????????????0?0??????0?10???????????2?????????1?10????????????????1???????????0??????????0???0101??011?0????112?0??????????????????????????????????????????????0?120?10??11100??0200000100???????120?02010001?11000?001001110????100001001??0?100????0??11?????????1???????????????????0???????????????????????????????????1?????????????????????????????????????1??????1????????????????????????????000?1[0 1]112111021104201?1

Stegouros_elengassen 00???????????00????10??1??01??0???1001?0???0??1???????????????????????????00?1011?0?0??01000???00?1?1?001????????????1??????????????????????????????????????????????????????????????????????????????????????1?00100?????0??????????????????????10???010?111000??0??000?0?1?0?0200??1?0????????????????????10??1010?00000001110?01111001111110000101?00?0?0?000101???????????????????????????????????00?????????????????????0101120001011????010???0???0???????10?0???????00?????0??????0??????????0?0?0???00000000000010000010000?00101010000001?0??0???220001000200010100110101110?0010200?0????00?00????????????????????????????0100000000100000??100010000200112?01?11?10?0???1?000100001020000001?10?1?1?1????001120?10?011100??21000001001000010120002010000????????????????????10?001101?001100??0?01011201200100100110001220101111000000?0000001?10000002?111101???1????1?220????00010?1110?1??00????1???0????10000011000?110??1??1011200?10000?100???1[0 1]112????211?420101

Mymoorapelta_maysi 0?????????????????????????????????????????????????????????????????????????????????????????????????????????????????????????????????????????????????????????????????????????????????????????????????????????????????????????????????????????????????????????????????????????????????????????????????????????????????????????????????????????????????????????????????????????????????????????????????????????????????????????????????????????????????????????????????????????????????????????????????????????????????????????????????001??0000??0???0???????2?????0??00???????0110111000010??0?100???0?00??????????????????????????????????????????????????????????122011??????????????10???????????????????????????????0210001111100??22000111011001??0120002010000?????????????????????????????????????????????????????????????????????????????????????????????????????????????????????????????????????????????????????????????????????????????????????????????1112?0????112?1?0?

Gargoyleosaurus_parkpinorum 001011100000?00????010010001??00?12001010000?00?00?000001????1???????????0?0??0110??100???????00?01?1?00????201???0????110?0000????0????10??0000?000??0?1?01000???00100?110??10?10?0?0?0?1?0???001????1????01100?00?????01?0????0??0??00??????010???0?00111000?0????0?0??1?0??010?0????1120?000?000??11?0011111????????????????01101??01011000?0000?110??0?0?010?0?01????0?00000000000001?00110?000?06000101001?2000?100?1101021200000113??10100011?0?0????00003?0???00?000???0?0??0??0????????????001000010?00000??002000001?0?0???????????????????????131?????02??01??00?101010?????10??0?????01???0????????????????????????????????0?00???0?0001?00?011001?00?????????????????????0?????????????????????????????0?12?010?001100??2200?101001000??112010201?0000110?0?0?100111???1?????????????????????????1200?001?0101????0120010??01000??010001001?100000?20?10??????0???????????????????????????????????00???1?1????????????1?????00???2???1???????111111112?120??112?1???

Gastonia_burgei 001011100000?00????0100100101?01102101010000000?0??000001????1???????????001??0110?0100101011000001?1?00????200???0????110?000?0???02??010??0000?000??0?1?010001?000100?1100?1001000?0?0010??0100100011????01100?00?????01?00???0??0?000??????010???0?001010000000000100?0001?210?0?1??11210000?0000011?001111??????????????????????????????????????????????????????????????????????????????????????00????????????????00?10010112000001021??0100?11?0?0????000?0??1????????????????0???????????????101??0?????????????????????????0010?01??10001????????141???10020001??0??001010000???0??0?????????????00002[0 1]001?00?011?1100110011?000000?000??001000101[1 2]00120?122?01?11??????????????????????????????????????????0?120010?011100??220?0?01001000??112010?01000?0110?000?100111???1010002100?0001110??0001011201200??0101????01220100??1000??0?0001001?100000??0?101?2??????00112?0?11?00???????????????????????????????????????????????????2???????????111?1111211?1?1212?1000

Polacanthus_foxii 0?????????????????????????????????????????????????????????????????????????????????????????????????????????????????????????????????????????????????????????????????????????????????????????????????????????????????????????????????????????????????????????????????????????????????????????????????????????????????????????????????????????????????????????????????????????????????????????????????????????????????????????????????????????????????????????????????????????????????????????????????????????????????????????????????00??10?00??0???0??????3410?1?00?0001?1???011010?000010??0??????11000?????????????????????????????????????????????????????????????????????????????????????????????????????????????01120000?111100??22000111001001??012010?11000101100??0??01?11?????10011001?000?110?????1011201?00100100010001220100?0?000100?0101001?10000002001000201?01001112??0?0?00??01???001?10???????????????0000????????1??????????????1??????0?????1112???0?02121??00

Nodosaurus_textilis 0?????????????????????????????????????????????????????????????????????????????????????????????????????????????????????????????????????????????????????????????????????????????????????????????????????????????????????????????????????????????????????????????????????????????????????????????????????????????????????????????????????????????????????????????????????????????????????????????????????????????????????????????????????????????????????????????????????????????????????????????????????????????????????????????????00?0?01??0????????????3410???00?0001??0??1????????0?100?0??????10000??????0?000???????????????????????????????????0???????????122?????????????????1??????????0??????????????????001020000?101100??22000111001001??0120102110001??????????????????????????????????????????011201?00???100????01???1????????0???0001001?1000001???000020??0????112????1??0??00?12001?10???????????????0000000??0??1???1??????200?100????0????1?112?????02?3110?0

Pinacosaurus_grangeri 000011010000?00????0001000001?01111001?10000000?001000101????1???????????0?010011000100100011??000121?0011112001?00?00?1000?0000?0?02??010??0000?0??????0?110?0???01????110?0?101?0???0?01??00111100111???0?1100100??0???????????0????00??????0100??001??10000??0??0000?00?000010??????1121100000000010?01111110100100000011?0?011011012110000001010?00000?0101010??1??000?00000000000000000000??00000????????????????10?10010212000001131110101010???0????00?10001??010000?000?0?00???0??????????0????????0000000000010?00010?10?00?0101??0?0???00100??34??????0?000?0?001?01?10100?01?0?0?100??01010??010000001100?011?10011?[0 1]11110000000111000110001012001210121100?1101040?00?10101010001200010011101101?13120001120010?001100??2200011[0 1]001001??012010?100000???????????1?11?????10010000?00????0??0???01120120010?10121000?????????1??00?0?0001001?1000000110000020??0?00?11220??0?000010?1000???01???01???0????100001????0?1100010001??2???10[0 1]010?01111111120121101111111?

Ankylosaurus_magniventris 0?0011110000?00????000?00010???0??20?1?11000000??0?000101????1???????????0?0??0110??100???????10001?1?00????2?0??00??????00??000???0???0????0000????????0????????001????110???101?0???0?01??00111100111???0??100?00??0????????????????00??????0100??002??10000??0??0000?00?00?01???????1121100?00010010?0111111????????????????011011012110000001010?00000?01010???01??1???00000000000000010110?000000????????????????10?10010212000001131?101000?0???0????00?0?00?1001?000?000?0?00???0001110010?0????????0?00000000010?100000???0012?01000000120???0???????????????1??00?001010?00??1???00?????01010??0000000011000011?10011011?1????00?0????00010001011001210??????????????????????????????????????????????????????????????????????????????????????????????????????????????????????0?10000?000???0??00?1011201?001??100010001220101?0???0000?0001001?100000021001??????????????????0?00?????????????????????????????????????????????000???????????????111111112?1210????1??1?

Agilisaurus_louderbacki 001000100000200????0000100011?00012001?10?00100?0?01000000?10001000000001?00000110?000011?01000100110100101?0000000?00?10000000000002?01?0??000010010100000100001000110001001000000000110000?010?0?00000000?000?000?????00000001101???00??????11????0?0??11002000??00???11?0??00?00????0????0000?0??0101??000010100??0000??10??01101????000010?010??1????1011010000?0???000000000000000200000???????050101010002?1?10101?110102120000011210101000?1?000????10?02?010??00000??00???00??00?????????1?0?000?010???????0??1??1?0??01?1?0???0??00?000???100??120???0???00010100?00?0?10??00?000000????100000?101011010?0011002??001110?00?000000??2?1?000001?1011120??00?00?00?1????????????0???????????????????????????00020000?000000??1?0000010110001100200?1100000111100?0?1010000?1101000?2???00?0002110010100?0?21???0???0????121?100011000??000200100?0??00?0?00????1???0????1?221????0001??1110?????1?00????00????000?00111???[0 1]010?0?0101100100?0?0?1000?00?000?0????????????

Hexinlusaurus_multidens 00???0100??0??0?????????????1????????????????????00100?000?11002010000001??00??110?000?0110100000010000100?0?001?00?01?10100000100000?1000??00001001010100000000?10011000100?00?110100000000?0????????0000001000000?????0000000010010?00??????11????0?0??11?00?0????0?0??1????????????????????????????????0000?????????????????01?01000?0000???01?1010?1?1000010100????????0?0??????????0??0????????0?????????????????00?110102120000011310121000?0??00????10?00?01??0?10?0??00????0???0?????????10000000?10?0?000?0??11?1?10?01010000?00000?000100100??111???000?0001?100100?01100000000?0?0????100000?101?210101001000100000111?00100000?????0000000?01?1?1100?00?000?0?0020?0011000000??1021?00000110000??02?20000020000?000000??1?00000101110[0 1]??0020000100000111201?01100000010100000020010011002?1001110020120?1??10?110?01210?0002?001?1010200100?0??0010001000??0000?01?1?22???000?010011100????1100??001011?0000?0011000?1010000?10110010000?0?1000?00?000?0????????????

Sanxiasaurus_modaoxiensis 0??????????????????????????????????????????????????????????????????????????????????????????????????????????????????????????????????????????????????????????????????????????????????????????????????????????????????????????????????????????????????????????????????????????????????????????????????????????????????????????????????????????????????????????????????????????????????????????????????????????????????????????????????????????????????????????????????????????????????????????????????????????????????00010??????0???000??00100?0?1??????????1?????0??????????0????1?????10000?????????????????????????????????????????????????????0000001011111000000?000?0??????????????????????????????????????????0????????????????????0?0??1100???????????????????????????1??0??????00?12???0011102010??1??0?0021010010011000121?100021001010102001001????????????0010010001111221011??0???????????????????????????000??01?00???????1000???0???01?????????????0??0????????????

Yandusaurus_hongheensis 0?????????????????????????????????????????????0?????????00?1?00?0?00000010?0100110000????????0??0?????????????????????????????????100?0000??010000010000000?000??000?1?????????????0?0??0000???00??0??????????????????????????????????????????????????????????????????????????????????????????????????????000???????????????????????????????????????1???????????????????????????????????????????????0?????????????????01?1?011212?00001?220?01010?0??00????10??????0???????????????0???1??????????0?????????000000?00011?00100010?0000?00??000???0???0??????????????????0??0????1???0?10????0???????????0?10???10?0???00??????????00100000???????0?0001011111100???????11???????????00?????1???????????????0??????0????????????????????????????????????????????????????????????????????????????????????????????????????????????122?1???21???0???????????0??0???0010????????0010?????????0?????????????????????????????????????????0??????????0???0??????000?????????????????????

Kulindadromeus_zabaikalicus 00?0??1?????????????????????200??????????????????001?0?001011001000010101???0000??000??001010001001000?010100001100?00?10000000?00000?0000??0?000001?100000000?0?1001100010010011?00?01000?0?0????????0?00?0?00?000??????000000?0001?100??????????????????100??0??????????????????????????????????????????00001????????????????011????0??00??0?010????????0[0 1]?0101?0?1???0000?0????00??010?00??????100?????0????3??????01?110112120?0001132?1?1000?????????????00?0???00?0000?00?0??????1????????????????????????00?0???0??????0???00???00??0000?????????????????????????0???????????00?0?00?????????00??1?111100??00?00000?00010??0??????????????00000?11111120?1?1?00?????????????????????????????????????????????00020?00?001000??0?001001011101??0021001100000?11?01?01101100???10?001120010011102110010100211?10??010?????0?????0???1001????02?0100????0?0??????0020???01??11221????00??????????????????????????????????00?????1??1??1????0000???????00?0??000?0????????????

Minimocursor_phunoiensis 0????????????????????????????????????????????????????????????????????????????????????????????0??0???????????????????????????0?????000?0001??1000?00?01100001?0????00?1?????????????????????????????????????????????????????????????????????????????????????????????????????????????????????????????????????????????????????????011010?02?01000?0001010?1?1000010100?1???0?00?10010000002000?????????0?????????????????0101101121200001?1??????????????????????20?????0?0000?010?0010???1????????????????????00??0?00?0?1????00???100?0?00??0100?000000??11?000??????????00??01011?00??????00?????10000??????1001?????????11???11?????????????????????????????????????????00040?00110000?00?1021100??????0000?03021000020?10??10000??1?0000010110001100200121??000111200?011011000111010011200100200021???11100100210?0?100110011210101121??101?00210100?0??0?0000110??2011?01111?200111?00010?11100????1???11????????0??0101?000??01??1??1???0010000000?0???00?000?0????????????

Nanosaurus_agilis 0?1??????0?????????0???1??0?1?00011???0?????00???0???0???????0?????????00??????100??0??????1?0????????????????0??????????00????????????0????0???????????0???????2??0?????????????????0?0???????00???????????????????????0??????????????????????????????????????????????00?????????????????????????????????00???010??1??01??????0110?000100000000001?1??1?100?010[0 1]?0?????0?0???????0?????0???????????06000?01001?0000??0??110102120100011?101010[0 1][0 1]00???0??0?10010?010?00?000?000???000010??????????0?0???0????0?000000010???0000101??00?00??0?????00111?02110000???0001??0010010?1?000010?0000????100000?00101101???000001000001?1200100000?002?000?000001101110?1?0?0?0????0?0?0?1000?000001???000000?000?????3?21001020000?00000???1?0000010[0 1]1111??00210111001001112010011011000111000000200100100021100111002102101?010?200?11221111[0 1][1 2]10010?[0 1]?0200100?0??0000001100??0??00??0112?1??0?000101113[0 1]01010110011001011000000001100??10100000101100100100001000?00?000?0????????????

Changmiania_liaoningensis 000000100000000????110010000200?0?000?????111?10000100?000?110010000000010?0000110000??11100100000100000001101?0000?10?10000000010000?010???000000000000000100102000110101000000100000000000?01???????0000000000100?????0000000000010100??????????????????????????????????????????????????????????????????000000101110001?01???0110????11000000110???????1000?1010??1???001?0??00?0000020001???10???0????101??03?????00??1?010212?0000?122?10?000?1?100????10??????0???????????????0??0??????????0?0000000101??00000???0?0?00??101??????0??0??0??00100??????01?0??????010?1?01001000001000???????101000?10102200010000002000001?2?101000000??1001000000010??110?1?1?00?10??????????0?00001010?0???0001100100??????0?0020?10?000000??0?0000110011?1??0021002000100??????????????????????????????0???0???0?1?100?????????????????1221?011?1???????0???????0????00????????????????1?2????0?011???1???????0??????????????00?000???????01??1??1???001?01010??000?00?000?0????????????

Yueosaurus_tiantaiensis 0?????????????????????????????????????????????????????????????????????????????????????????????????????????????????????????????????????????????????????????????????????????????????????????????????????????????????????????????????????????????????????????????????????????????????????????????????????????????????????????????????????????????????????????????????????????????????????????????????????????????????????????????????????????????????????????????????????????????????????????????????0??????????0?000000010?0?00?0????00??0??00?0???00?????????????????????00?0010010110010??0?0????00??????01012010000?00011010011??0?????????????1????0?1??01??0???1?????0?????????????????????????????????????????????????????????????????????????????????????????1?????????????010?0???????????20?0?????1??002??210????0??????122111?1011?1??1?0200?00???????????????????????0??221????00010?1110??????????1????????????????????????????????0??000?????0??????000?0????????????

Albertadromeus_syntarsus 0?????????????????????????????????????????????????????????????????????????????????????????????????????????????????????????????????????????????????????????????????????????????????????????????????????????????????????????????????????????????????????????????????????????????????????????????????????????????????????????????????????????????????????????????????????????????????????????????????????????????????????????????????????????????????????????????????????????????????????????????????????????????????????????????????000??00??0?00??????????????????????????????????????????????????1?0?????????????????????????????????????????????????????????????????????????????????????????????????????????????????????????????????????????????????????????????????????????????????????????????????????????????????????????????????????????????????????????????????02010101101????010100???????????????????????????????????????????????????????0??????????????????????????????

Koreanosaurus_boseongensis 0??????????????????????????????????????????????????????????????????????????????????????????????????????????????????????????????????????????????????????????????????????????????????????????????????????????????????????????????????????????????????????????????????????????????????????????????????????????????????????????????????????????????????????????????????????????????????????????????????????????????????????????????????????????????????????????????????????????????????????????????????????????????00000??21?0?0??0?0??00??00200?00??0?10??????0??????0??1?????????????????0?00??????0??????0000210?0001?00??00010011?101?0001000????00000?0111112011?1???0????????????????????????????????????????????????????????00??????????????????????10???????0??????????????0??????00112001?????????????100?1121???0???????11221?111111?1??1102001???0??000010100??2010?01101?2???1?10?????????????????????????????0????????????1???????????????????????????000?0????????????

Orodromeus_makelai 00?0?01?0??0?00????00???00?1???0?1?001?100?10?10???1?00001011002100?00001100000110000??11??1200?001000111??00100?00?00?1011?00000000??00010020000001010000000000100010000100?000110000010010?0?01001210??0?00000?00?????0?00??01?0001100???????10111000?111000?0010001?11110???01???0?????0?????????????0?00000?1??????0???1???0110100?10001100010101??1?1000010100?1???000001?0000000010000????0???050?010100?010???001?110102120000011120101000010?00????10010?01???1??01??00?0?000010?????????100000100?011?100?100210100?0[0 1]?010000101200000?000?????31?000100?0021?1000001011000001000010????100000?0010220100011000100010012000100000000?0?10000011110112011110001?0010?01001?000?????10201000?????01000030??000020?00?00000???0011?1010?11?1??0021001100100111201001101000011?00000120010020002110?11100210210100100111011220?111011?121110200?00?0??100010101002011101110122101010001011130110??110011000[0 1]01?00????011000?1?1??0??1011000000000?1000?00?000?0????????????

Oryctodromeus_cubicularis 0????????0?0100????11?0100011?00011001010???00??????????0????0?2????0?0001?0????10?0?????????0??10100011?????1????0??0??010?0000????0?000101100000????0????10??0?0?0?10????????????000010000?0?1000111?????0?????????????????????????10????????1????000?1?1000??01?001011110??001000?????????????????1????00???01010100010010[0 1]001101000100000000[0 1]01?1??1?10000101?0?1??????????????????????0????????05????????????????01??101021201000?112010?00?????00?????0?10??1??00??01?110?0?00?0???????????????????????10100?10021??00?001010002101200000?20?1?0??32?001100?00210?0010010110100010000?0????111100?000021010?0110002000100?12[0 1]01000011??1?01011000011??11011?20000?000??0?0????00??????0?00000?????010????????0102000??0[0 1]00????0?11100[1 2]011?01??0021002000100?112010021010000?1?0000012000002000211??1?1002112101001011110112201111011?111110200100?0??001010?000020011011011221?101000101111001?10??????0?0?01??0000?011000?0011?100101100100100001000?00?000?0????????????

Zephyrosaurus_schaffi 0????0100??0100????110?10001??00?11001010??100100?????0?0??1?0???10??00011000?0110?00????????00?00100111?0?00110??0?00?1011?000?10000?010100200000??0??0?0010??0?000?00????????????0?000000??00000?11?0?000010???00??????????0??????1?0????????1011000001110000?01?00101111001001000?10?????00???????1????0000?????????????????????????????????0????1???????0????????????0??0??000?000??000?????????05000101?103????1?01011010212000001122010101010??00????100?????0?0???0?0010???000000???????????????????0???????0??200?????0?????0?1?????????0?0?????????????????2????????1??1??????????????????????????????????1????????????????1??0??????????????????????????0???0????????????????????????????????????????????????????????????????????????????????????????????????????????????????????????????????0???????1??1?????????????????0????1????1?????????1???0????1????????????0????????1??1?10??????????0?????01?0????????0??????????????????????????????00?????????????????????

'RTMP_2008.45.2' 0??????????????????????????????????????????????????????????????????????????????????????????????????????????????????????????????????????????????????????????????????????????????????????????????????????????????????????????????????????????????????????????????????????????????????????????????????????????????????????????????????????????????????????????????????????????????????????????????????????????????????????????????????????????????????????????????????????????????????????????????????????????????????????????????????000????????0?????????3100??????0?21?10?0?010110100010?????????10100?????????????????????????????????????????????????????????????????????????????????????????????????????????????01020?00?000000??00110011001101??0021001100100?11201?0??0????????????0??????????????0????001??21????1???????1221101?0?1?1??1?0200100?0??????????????????????????????????????????????????????????????????????????????????????????????????????000??????????????

Haya_griva 0?1000100000110????10?0100011?000110010?0001101000011000010110010000100011000001100000?1110100000011001010000100000?00?10111000000000?1001??010001010110000?000010001100010000011100000[0 1]00?0?0?01001210000001000000?????0000000000000100??????1101?0010?111000000??001011110???0000?0010??000011000101000?000010101010001?0101001101000110100000101010111100?110?00?1???00000010000000010001?00?0000050101010113????01010110102120000011[1 2]2010100021?100????1?010?010?001000??0??0?0?0?00?????????100000000?0?0?000000010?0?0000101?00??002?0000??01100??220??0?01???2?010011010110100000??010????101000?001022010?001000200000111?102000010001001000001011??111?10100?00?0?0???0???000100011???????000?0?0?0?0????00[0 1]020?00?010000??0?001011001101??0021002000100111201?0110110001110?0001200?0020002110???10021121010010111[0 1]0112211111011?111010200100?0??110000100?02?0???1?0112210?0?00?10?1110?1?10?????100100???0000001100??001??1??1011000000000?1000?00?000?0????????????

Changchunsaurus_parvus 00?0?0100?00100????11??100?11??001100101001110100??1?00000?1100?010??200110000?110?000?01?01200?00110?1?1??????????????1110100000?000?1000??11101?0?011000??00001000110001000000000000000000?010??011100???0???0??0?????0?0???0000010100??????????????0?????????0???0?0?11?001000000???0??01010?00110?010?0000001111100110010100110100011010000010?010111100011010001??000100010000000010001000?001105???1010113????0?01111010212000001122110100001?100????10010?110?001?00?000?0?00?00000??1?011100001?0010?0?000?1?010?0000?0101??????021??0??00110????10?????1?0??????????????????????????????1????????1022000?0?00?0100??0????002?0001000??0101000011????????????????????????????????????????????????????????????????????????0??1??????10?1?0??????????1???????????????????????????????????????????????????10210100101110011221?111?1??12?110??0????????0???????1020?1?0111??????10000????1????????1??0?1????????0000?0110?????1??1??1???00100100???000?00?000?0????????????

Jeholosaurus_shangyuanensis 0?00?0100000100????1100100012000011001010101100?0001100000?11001010000001??0000110?000?01101200[0 1]001?00100??00100000?00?10100000000000?0000??1110000101100001000010001100010000011000000000?0?0100001110000001000000?????0000000100010100??????1100??0?????1000200???0?0?11?001000000?1????01000100???1????000010101110001?0100?0[0 1]10100?1101000001010?001?1000?10[0 1]00?1???00100010000000010001?00?0000060101010113????1?0?0110102120000011220101000?1?100????11????010??1?000?????0?01??0??????????100001000?010?00000001000?00?0?0?0002?00210000?????????221000000200?1?100?10000?00000100?00?????10000??0?11120?0?00?000???0???01?0?2?0??1???2?0????001111111201??????0????????????????????????????????????????????01020000?000000??10000011001101??0021001100100111?0???11011000111?0000020000121002?110101002102101001011100112221111111?121110200100?0??001000?001?20[0 1]01?1111122101000001001130010101?00????1[0 1]???000000011000?101??1?0101?0010000000?000?00?000?0????????????

Nevadadromeus_schmitti 0????????????????????????????????????????????????????????????????????????????????????????????????????????????????????????????????????????????????????????????????????????????????????????????????????????????????????????????????????????????????????????????????????????????????????????????????????????????????????????????????????????????????????????????????????????????????????????????????????????????????????????????????????????????????????????????????????????????????????????????????????????????????????????????????????????????????????????????????????????????????????????????????????????????????????????????????????????????????????????????????????????????????????????????????????????????????????????????????????????????????????????????????????????????????????????????????????????????0?112101001011110112211121211?111?10200?00??????????????????????????????????????????????????????????????????????????????????????0???0??????????????????????????????

Parksosaurus_warreni 0000?01?0?????0?????????????1?0??????????????????0000???0101100?000?00001100110110000??0110120000?1??????0??0?????0????1000?0000?0000?0001??0?00[0 1]10101?00001?0001000110?0010?0010100101?0000?0?010011?0???????????0???????0???????????00????????????????1110001??00?01?1???0??00??0??0?0????011??????10???000?1?1??????????????011????0?10100000100?1?01?10000101?0??????0?00?1000000001000100?????00?????????????????01?11011212000011132?10?000100101??0010120?010?0?00010?1[0 1]0100001?1???????????????????000000001001??1?1?????20012000100010??00111??211???000??0010000010100110000?0000?10000110100?011112000000?00??00000101?00100000000??0001001?01??11???111?00?1000?????????????????020????????????????????01020?00?000000??00111011001101??002100?1????0?11?????1?0?200?????100012000002110201??1010020021?10?1000010112221111111?1001?020010??0??00?00?11110201010011112211?00000100111001?10110??1001001??0000[0 1]011000?1011011010110000000000100??00?000?0????????????

Thescelosaurus_neglectus 00?000100100200????1101100011?0001100101000110100000100001011001000100001100000110000??0111020000011001010100110000?01?10110000100000?0000?0010000010110000100002000110001000001010010010000011010?1120000001000100?????00000001?0010100???????1101020111110011001000101101001001000000????001111111?1000?000010101110001?010100110100?210100000101010010100001000001??0000001?010000001000000010000060101010003????11010110102120000111320001000110101010010000?010?0100010011010000010?????????100?010?????0?000?00?11?1?10001?2001??01000000?011111013[1 2]?00?????0011?100110111?000000000011???0?10100?000021000?001000200001111110200000?0110010?001?011111?00100?010?100020?0011000100101???1000000?00001?03120000020?00?0?00????1?0000?20011?1??002101010000011120100110110001??00?00120010020002110?1100021021?1?0?0?????112211111111?1????02011?0?1?000?0??1?01??0??1??101?22101000001001100010?0110011011001??00000011000?101??1101011100000000?1000?00?000?0????????????

Thescelosaurus_garbanii 0??????????????????????????????????????????????????????????????????????????????????????????????????????????????????????????????????????????????????????????????????????????????????????????????????????????????????????????????????????????????????????????????????????????????????????????????????????????????????????????????????????????????????????????????????????????????????????????????????????????????????????????????????????????????????????????????????????????????????????????????????????????????????0??1????????????0???????0?0??????????????????????????????????????????????????????????????????????????????????????????????????????????????????????????????????????????????????????????????????????????????????????????????????????????????????????????????????????????????????????????????????????????????????????????????????????????1?00????0??????0??????01?2????000001001100?1??00?0??1011001??00000011000?1?1??11?1011100000000?10???????????????????????

Thescelosaurus_assiniboiensis 0????????????????????????????????????????????????????????????????????????????????????????????0????1?001??0??001?100??0??000??????0??????0???01?0????????0????????????????????????????0?????0?????0????0?0?0?10???00?????0?00??00000???00???????11??020111?????1?00000101111001001001100?????0??????????????0?0?????????????????0????????1??0?0?0?0???0??????????????????????????????????????????????0????????????????????????????????????????????????????????????????????????????????????????????????????????????????????????????200120000?000??211??????1????0???0???????????????????????????????1????????????????????????????????????????????????????????????????????????????????????????????????????????????????0002??00?000000???0?10011001111??002101????0?0?11201001?0?100???????0???????????????????0002102101001011100112211111111?1111102?1?00?100001000?00002001?0010012?10100000100110001?1011001101?001??00000011000?001001101011100000?00010??????000?0????????????

CMN_8537 0????????????????????????????????????????????????????????????????????????????????????????????0???0????1??0??000?100?????010????1?0?????????????????????????????????0??????????????????????????????????0?0?00???????????????????????????????????????00?11??10???????????????????????????????????????????????0??1?1??????????????011010002101000001000?00?0100001010001??0001001101000000?0000000?00000?????010103???????1??10102120000111???10?000?10101?1001000???10?0???01001101000??10???????????????????????00??0??11??????0??2001??000?00?0?0111????21?00?????0??1??001?0111100100??0?01100????010??00102200??00?0002??0?1????002?00?0?????0?010010011111?00????????????????????????????????????????????????????0020?00?000000??1?0?001200110????0????01??0?0111211?0?????0001??00?0??2????02?002110?11000?1?21?????????????2211111111?1????0201???????0??0????????0??1???0112?1??0?00[0 1]10011000???01?0??1001?????00000?????0??01??11?1???1???0??????????00?000?0????????????

Goyocephale_lattimorei 0???101???0??00?????0?0100??1??0001001????000011?01000?10????00????00???????00?11????????????00??0111?00??10?000??1?10?1????00???10?????00?0001??0????00???10??0??00?0????????????????0????????????????????211?010100000111100????????11111111???????????110?1?????????????1????0?????????????????????????10001?1??????????????01101??00002000?11010?001?000000000001???001000?0000000011020?00??0??031101011001?1?21?0[0 1]??101021200001112??1010?0110?01?10?0100???10?00??01100000??1?000???????????0?0??????????????????????????????????????????????????110???001?1????????0??????????0?000???????1111?????????????????????????????????????01??11010010011111101??0???????????????????????????????????????????????????21?111000021000?111001101301???????????????????????????????????????????????????????????????????????????????????????????????????????????????????????????????211?????0???????????????????001011?0?00???????0?1??00?101???0???00?0?0?000?0???????????????????

Homalocephale_calathocercos 0???10?????????????????????????????????????????????0?0?11??1?1????????????????01?0?00??0????10?000111??01??02000??1????110?0010?01002?00001?0?10201001000001001100001010010001000100100100?0?000000011?????211?010100000111100000101011110111111???0110?111002?000?1100????1101?011????0??01111100110?001?1000??????????????????????????????????????????????????????????????????????????????????????0?????????????????01???01121?0????1??11?01???11?0??????????????0??????????????????????????????????????????????????????????????0010?11100000?200??0??211000000?1111?00?0?0010110010100?0???????1111?????????????????????????????????????01??????????????????????????????????????????????????????????????????????0102101110000211102110010101301??00210?1100100?11110?1??00201??????1102211?0???1????0????0011120??0??0?12001?????????????????0100100?1010000?0?0??????????????211????100?10?120?1?10??????????????0???????????????????????0???????????00??0?000??????????????

Pachycephalosaurus_wyomingensis 0??000100000?00????00?0100011?000120010?1100001[0 1]000000011??1?111???0??0??000??0110000100?00?110000111?001?002000??111?1110?1000001002??000??0010201001000001001011001[0 1]10010001001100100000?0?0100000111???2211?01010000010110000011??11110000111?1?01?0?111001?0???1100??1??0?1????1???0??01100000100?001?1000??????????????????????????????????????????????????????1????0100??0000?000?101?????????00????????????????00?110102120100011311?0100011?000????11??????????????????????1??????????????00000?0??0?100001000[1 2]0?0010?0????01??11??100????0??????????????????????????????????????????????????????????1????0???0??????0??????????????????????????????????????????1????????????????????????????????????????????0????110000??????1???10?11301?????10????????????????????????????0?????????010000??0?11000??1??????????????122??1??01??1????0100????1?????????1????????????1?2??????10???????????????????????????????????????????????????0???0??????000?00?000?0????????????

Prenocephale_prenes 0?0110100000200????00?0100001?0100200100110000110010000101011111101?000??0000001100000?01001100000111?001?002000??111?0110?0010201002?1000??0010201001000001001101001010010001001100101100?0?0000000111???2211?010100000111100000101011110000111?1?01110111002200021110??101001?0111???0?101111000100?001?1000??????????????????????????????????????????????????????????????????????????????????????031001101011?1?01?01?1101121?0??01?1301?01????1?00101?1???????????????????????????10???????????????????????????????????????????????1?????????0??????2?????00??11??????0??????100??????????????1111??????????????????????????????????????????????????????????????????????????????????????????????????????????????102?01110?0021110?11??10101301?????10?1???1?????1???1????????????101022???0000100??0?1??001?1?0?????0?????0???????1?????????0100?0??????0?0???0??????????????????????????????????????????????????????????????????????????????????????00?????????????????????

Stegoceras_validum 0??110100000200????0000101001?010020010011001010011000001??1?1????????0??00?2101100001210000100000111?001?102000??101?0110?1000201002?1000??0010201000000001001001001000010001001000101000?0010000?0111???2211?01010000010110000011??111111100110?001100111001?000?1100??11100110111???0?2011110001011000?10000?1??????????????01101??000011000110?010010100000000001??011100110000000011000000??00003000001100210201100?110102120000011300101010110000????10012?010?000001100000?00??0[0 1]001111000?????????????????????????????????001??11??0000??00???????????0?????????00??00100100?11?000???????1111??0021110001000000100000010200?0?00??????1100002?01?011?01101?00011?1????????????????????????????????????????0?0200?1100012101021110011?1301??0021001100110??????????????1?????1010221000000100??0?1?10?0??20????????????1????????1????????100????1?????0?0?1???????0????1?2????0?10????????????????????????????????0?1?0??????????????00??00?????000?0??000?0????????????

Wannanosaurus_yansiensis 0???1????????????????????????????????????????????????????????????????????????????????????????0??0?1?1??0?????0????1?????10?0000?000???000010001000?0????0?010??0??00100???????????????????????????????????22?????01?000?10110001?1010?11100000?????02?0???????????????????????????????????????????????????10001?10??10?00??0???01101??00100000?000?0?001?100000010001???00110010000100010021000?00??0?????????????????00?110112120000010??????????????????????20?00??001?00?000?0?????????????????????????????0????00010??01?????????????????????????????????????????????????????????????0?????????????????????????????????????????????????????11000010011111201??????????????????????????????????????????????????????2???110??????????????????????????????????????????????????????????????????????????????10011???????????????12??11?101??1????0101?0??0?????0??????????1????01?2??????1????????????????????????????????????????????????????0???0?0?????00?0???????????????????

Chaoyangsaurus_youngi 0??11???1?????10?010000??1?0????00100100??00?00????????????1??1???????0??000???111??00???????0??0??????????????????????????????????10?1000??0000??0?111001???0102000????01?10000?01?1????0?????1???021????????????????????????????????????????????????????????????????0???????????????????????1?010???0???000?00100000000?0111??11110?00002000?10010100??10000100000001??1?00?00000000120010111100?0020000110013????1?01?11010212000001101112111111?000????1?020?0?0?001?00?0?0?0?????00??????????0???000010?1?000?00010?0001?0???????????????????????????????????????????????????????????????????????????????????00?00010000111??0?????????????????00001????????????????????????????????????????????????????????????????????????????????????????????????????????????????????????????????????????????????????????????????????????????????????????????????????????????????????????????????????????????????????????????????????????????????????????????????00?0???????????????????

Hualianceratops_wucaiwanensis 0???????????????????????????????????????????????????????0???00???11????????????110000?2?????10??0???????????????????????00?000000?11011000??00101000111101010010?000?0??010?0000020000010001??1?????210????1????????????0?10??00?1???1010000????????????????????????????????1?????????????????????????????00?0?0100000000?01111011110?00?02000000010?00??10000101?0?00110???0??0????0???1?1??1???0000?????????????????0??1?010??200?001??1??0?1??????????????????????0???00?0???0???????????????????????????????????????????????????????????????????????????????0????????????????????????????????????????????????????????????????????????????????????????????????????????????????????????????????????????????????????????????????????????????????????????????????????????????????????????????????????????????????????????????????????????????????????????????????????????????????????????????????????????????????????0????????0??????????????0???01000??00??0???????????????????

Xuanhuaceratops_niei 0??????????????????0???1?1?????????00101???????????????????????????????????????11???0???????????0???????????????1?0?????000??0???0?????000????1??0????????01?010?000????01?10000?11?1????0??????????210???????????????????????????????????????????????????????????????????????????????????????????????????00????10????????????????11???0??2?0001?0???0??????????????????????0???0?0??01?111?1?11????01??0????????????????1?01???20???0?????02??????0??0??????????????00??010????????0??0???????????000????????????????1?????????????1???????????????????3???????0???????????????1?10????????????????????0?2??1??0?00??001000011?1?0??0?0????????1?1??0??1??????????????????????????????????????????????????????????????????????????????????????????????????????????????????????0??????0?1??????????????????????????????1???????1221????1?????????????????????????????????????????20?????????1??110????????????????????000?01?????????0?0?????0???????????0??????????????????????

Yinlong_downsi 01011210100010101000010101001001010001101100000?0011000001010011111000001000000110000020110110000010010100000000110?01?1000100000011011010?0001000001[0 1]10000100100000000101010000000011001000011000?0210110111010001100000010010001010?110000??1101?0?11??00100?00??01000110110102110?????10101100111010110000000100010000001110011110000002000000010?011010000100000001?011001100000001211101101??0?03111111001110201001111010212000001111012100010?000????10010?01??0?1000?000?0?00??1??????????100001?00?00??0000000?0?000000100?00??00??1??0??0?1?0??2??00000??010??100??0101101?1010000?0???110?00??002[0 1]11000000?100100001[0 1]1??00?????????2?0111000001?1?12001020000?0000?010???0000001000200??0001100000?0????0010210010001000??10011[0 1]01011101??00210001001?0?111?2002?00?002??10000122001000100200121?10021021?1?0????????12211011110011??10200101?0??00?0000000010??0?0?11122[0 1]??0?0001??1??????10?????1000?01??000000000?0??01?01??10110000000000?000?00?000?0????????????

Albalophosaurus_yamaguchiorum 0?????????????????????????????????????????????????000??000???0??????????1??????101000???1?1??0????100101????00??000???????????????????1?????0?00?00?0??00?0?0?????00?00??????????????0???00????0?????????????????0????????????????????????????????????1??000002????0???????0????211???????????????????????00?????????????????????1?????????????0?0??1????100?010?00?1????????0??????????0???????????0?????????????????01?1?0102120000011?11121010?10010????11?????10?000?00?000?0?01?0?1?????????????????????????????????????????????????????????????????????????????????????????????????????????????????????????????????????????????????????????????????????????????????????????????????????????????????????????????????????????????????????????????????????????????????????????????????????????????????????????????????????????????????????????????????????????????????????????????????????????????????????????????????????????????????????????????????0??0???????????????????

Micropachycephalosaurus_h 0????????????????????????????????????????????????????????????????????????????????????????????????????????????1????1?????????????????????????????????????????????????????????????????100?0000??1?????21????????????1?????????????????????????????????????????????????1?????????????????????????????????????????????????????????????????????????????1??????100?01?????????????????????????????????????0?????????????????0?11?0102120000011??????????????????????????1????0?00?000?0???0??????????????????????????????????????????????00??????0000??????????1?00000000?1?????????????????1?????0?????????????????????????????????????????????????????????????????????????????????????????????????????????????????????????20000?0????0???????????????1??0?210??100??????????????????????????????????????????????002112101??1???????122111?1110012?0?0??0?00????00000011000100000110?????????????????????????????????????????????????????????????????????????????????????????????????

Stenopelix_valdensis 0?????????????????????????????????????????????????????????????????????????????????????????????????????????????????????????????????????????????????????????????????????????????????????????????????????????????????????????????????????????????????????????????????????????????????????????????????????????????????????????????????????????????????????????????????????????????????????????????????????????????????????????????????????????????????????????????????????????????????????????????????????????????????????????????????000?1?0??0000??001?0??21?000?00?1111??000?0?00101000?000??0?????0000?????????????0????????????????????????????????????????????????00??0??????????????????????????????????????????01021?010001100??1?001111011101??0021000100000?11102?0?????00[1 2]1??00001220001001110??0?11000211??????????????1?2?101?11001??1?????????100??0????0????????????1?22???0?0001????????????????1????????0???100??00?[0 1]01??00?11??00000[0 1]000???????0?000??????????????

Psittacosaurus_mongoliensis 01010201110001100010000101013101211001101100000?110010001??1?111???00?00?0001101110000?0100?10000010010100100000000?00?10001100100110?1000??000100001110110100100000000111001000000000000001?0100000110100001010101100010000000110010100??????0101?001?0110100?0???0100????011200000???0?10?0100010?1?0???00000010100000010110?111110??00020000111??101?0100011010000?11011000101000000200100101000000????????????????01?1101121200?00111?112??00120000????01?20?010?000001100000?0100?1002212020100000000?0???????0??1??0?00?0100?01??00?0000???00100??2??000?0??000101000?010?1010001000010???1100001?0010100000001100100001112?01101?00?00100100?001011111??0111?00?0101030?00010000001?102011?000100010000110?001020000?001000??0?[0 1][0 1]1011001101???02100[0 1]100100?11101?02100100210?01001220000000000??1?111001102??1?????????0122??12111??1??1?02001????01???0?0?000?????0????1?2????0?000?1?????0????1??0??0000????0000000000??001??1??11??000000000??000?00?000?0????????????

Psittacosaurus_lujiatunensis 0?010201110001100010000101003101211001001100001011[0 1]000001??1?111???00?00?0001101110?00?0100[0 1]1000001001010[0 1]100000010?01?10001100?00110?1001??000100001110110100100100000111001000000000011001?01000001101000010101011000100000101100??000??????0101100110110100201000110?0000112000000110?00101100111110???00000010100000010110?1111100?0002000011110101?0100011010011??1011000101000000200100101000000???????????????001?110112120000011011121100120000????01?30?010?000001100000??1???1?????????10???000??0???00??0???0???00?0??0?01??00?00001?200?????[2 3]??000?00?00010111??011000?0101000?20???1?0??0??01111200??0011002010011?2?011?10?00??1001?0?001111?????01?0?01001????????????0?001010?0???000?000?0000????001020?00?001000??[0 1]?001011001101??0021001100100??????????0???0?????0001221000000000??0?1110??10?????????????0122??0?111??1?????2001??????0000????????????????1?2?1??0?00??????????????????????0????0000000?000?001??00?11??0?0000000??000?00?000?0????????????

Beg_tse 0??112000?0000??????0???01??310?1?100?????000010???000?0010100001110010?10?0??01100001?0101010000?????????????????0?????????10????1001100???0010?00010001001?0100000100?01?00100??0?0???00?1?0?000001??????????????????????????1?????????????????????????1?0??????????????????????????????????????????????000?0?1?1??00???01???11111???01020000110???1???101?110100?1???0020100100??011?1010?????????40?01001013???????1????11?12???0??????121????2?0?0????11????0?0?????21?0??0???1??0??????????????????????????????????????????????????????????????????????????????????????????????????????????????????????????????????????????????????????????????????????????????????????????????????????????????????????????????????????????????????????????????????????????????????????????????????????????????????????????????????????????????????????????????????????????????????????????????????????????????????????????????????????????????????????????????????00?0???????????????????

Aquilops_americanus 0??1????0?00201111000??1010030000100011???0001100000000000?110010110010?10?0000110000120100110000010010111100110110?00?110?0000000000??000??000020000?00100110?0???010????????????????????????????????0??1?1????1????????0????1?1?0???0?????????????????????????????????????1?????????????????1???????????00???01?1110010001?0???1111?0000??0?0???1??111?????????????????????????????????????????1??031001111013????0001?1?011?12000011101112?0?1121010????11?2??0110011?210020011?1??11?????????????????????????????????????????????????????????????????????????????????????????????????????????????????????????????????????????????????????????????????????????????????????????????????????????????????????????????????????????????????????????????????????????????????????????????????????????????????????????????????????????????????????????????????????????????????????????????????????????????????????????????????????????????????????????????????0??????????????????????

Archaeoceratops_oshimai 0?01121000001011110000010100100002100110110001100000000001010011111?110010?00001100001?01001100010100101101?0010110?01?1101100000010211000??0010200000001001?00010001010110001101000100100000110100022011111?????01110?1?0?2?1??11010100??????11?1??0????10100?1???01?001000011??1?1??????0??110?1??1?????000000111010110?0111?011110?0010200000101011???101011000011???002010?1001101120020010000?1031001111013????11011111112120000111111121111?20000????11?10?011101102100200???1??01????????????0??????????????0???????1?????0?01??10??1?0???0??????211000000?01110?0????????0??11???0???????????0???????????????????????????????????????????????????????????????????????????????????????????????????????????????020000?011000??00111011011101??0021011100100?111?1?02?0?10021??0??????????0[1 2]?000??1?11?????1?1????????????1?2?112121??1??????????????????0??????????????????2???????00110112001010110011???0????0000?010000?0?1001?011??00000[0 1]000??000?00?000??????????????

Auroraceratops_rugosus 010112110000[0 1]011110010010100200100100110110001100011000001011011111001001010100110000120101110000010010110100010110??1?1101000000010210000??00101000000010011000001010[0 1]01100011000001101000001000000210111111011101111010002111111010100??????1111?0111?110100200???12001000111????1???0?00111101?0011011?000010111110100001111011110?0000000000101111111100011010010010000111010011011?0020010110010311001?1013????10011110112120000111111121111?20010????11010?0110011021002000?010?1??????????101101000110100000000101001000011001??11001011?200100??3110011000011101111?011100001110101?0???1[0 1]?0?01100101010000001001000011111001000?0?00100001000?010001200122001000000?0?????000000?010211000001100100?021100010200010000000??0?111011011101??0021011000110?11101002?0010021???0001220011000100??1011100110210100101??1011221111111001111?0101101?0??0000001001??0??0?0111?22?010100011?1120?1?101?00?1001011??0000[0 1]01?000?1?1??10?11??1000100000?000?00?000?0????????????

Liaoceratops_yanzigouensis 0??1021000001011100?0001010?200001100110??0?01100001100000??00?1101?0100100?01?110000120101110001010010111100010110??1?11010000?0000210000??00002000010010??1?001?0010101100010?1000100100?0?00?00?021011111101110111101010201?111010?01?000??1101??1?1??10100210??01200110011101101??1??00111100101110?10000000101000010?0111?011111?00000000001110111?110001101001001000201000000001120120110101010310011110111[0 1]2201011110112120000011111021111?20010????11010?0111011021102?0???10?01?????????????????????????????????????????????????????????????????????????????????????????????????????????????????????????????????????????????????????????????????????????????????????????????????????????????????????????????????????????0???????????????????????????????????????????????????????????????????????????????????????????????????????????????????????????????????????????????????????????????????????????????????????????????????????????????????????00?00??????????????????

Mosaiceratops_azumai 0?0?12110000211011?000?101001?0012000110??0001101????0??0101000?1010010010?0000110?001???????0??10??????????????????????00?010000010011001??0010?00010001001?0000000?01?11?011?0?00???????????????????0????0????????????0???????1?01??00?????????????????????0??????1????0?0112???????????????????????????00??00101110110?01110011110??0?020000111???11??1000110100?001?001?1001?011011?1020?1???10??0??????????????????????????????????1?????????2?010???????2????0?0????100200???????????????????????????????????0??1????0???????010??1??1????????????????????????????1??0????????0??0?????????????0?????????????????????????????????????????0??10?0??1???????????????0??????????????????????????????????????????0?020?00?000000??0?111011011?01????21011100100??????????????0?????0001120011000100??101?100210??????????????1?2?11??????1?????10?????100??0??0?????????????01?2????0?0?0???????????????????????????00010???????01??1??????0???0??0????0??0???????????????????

Yamaceratops_dorngobiensis 0?01111100000110111000010101210111000110110001??0011000000?100011010000010?0000111100??11111100110100?0110?00010110?01?110100000000021000012000120000000100110000010101011000110?00011110000??10000021011111101??01110010102?1111101?100??????010??0210?110100??0??0120??00011101?01??10?001111011000?0?1?000000111110010?01110111111?000020000110?0?11?110001101001001000011001001001120020010??1000?????11101?00111?01?11011212000001111?0211?1?20010????11020?0110?1102100?000??1??11??????????????????????????????0????????????0??????????0??00100??311000?0????????11??011100004110????0???110000?????????????????????????????????????????0?????0?011??10??????0?000??????????????????????????????????????????00020?00?001000??0?111011011101??0021011100100??????????????????????????????000000??00?1100?????????1???????122?1??11???1????0[1 2]?0???0???????????????????????1?2?1???000???????????????????????????????????????????????????00000000???000?00?000??????????????

Leptoceratops_gracilis 01?11110000010111100000101001?00000001101100111001000000010100011110010010100001100?01201000101010100???111?0000110??1?110?000000011211000??0000200000001001100000100000110001101000111?0000??1000002201112110101011110001021111111??100??????110010110011010020011012001000011011010000?001111011?1111?1?00001011111011000111?111110000122000011010?11?1100111000011??1101010000010111201200101110000???????????????001111111212000011111112?111120010????1??20?011201?0210010011???0?111221202010110110011???000?00??0???00??110001??0??01101?200100??2??001?00???110?11??0?100000111011??0???010?00110?2?021?010?0000?00001101?011000?0?001001110001011?00200122?01?1110020000110000001010201000011100100003110001020??0?000000??0?111011001?01????2101?100100011001?02100100210?00001220011000100??1?1110??112????????????????1?1???1????????10??0??1001?01001001?????0????1?221????00011?1120?1?10??????0???????000000000????01??1??11??10000?0?0??000?00?000?0????????????

Protoceratops_andrewsi 01?11201000010111110001101001?010200011?1100011001000100010100001110010010100001100?01210000101110100101?0100000110?00?11010000000112110001?000010100100100110000010000111000110000011110000011000?0110111311011[0 1]11111110[0 1]02210111010000??????11???02100?101002000??120???1011101??10?0??0011110111?111?1?00001?11101011000111?111110??01020000111??11111100111010011??11011000000101112012001010101020001101113????0?111111112120000111211121111?21010????1?010?011101102110100110???01012212020101?01110?100?000?00?1000?1000110001??00?01010?20?100??3111011000011?01111?0[0 1]200000[3 4]11011020???01???01000101[0 1]0001000000100001110100100001?00000101000101[0 1]001200112?0101000020?00110000001010200000001100100003121001020000?0[0 1]0000??0?111011011101??0021011100110?11001002100100210?0[0 1]0012200[0 1]1000100??1?1110011121???1????????1221111111001211?01?11???1????10???0?0020??0???11?221??0?0001101120?1?10??0??10?1?????000000100?0?001??1??11??100010000??000?00?000?0????????????

Ceratops_montanus 0????????????????????????????????????????????????????????????????????????????????????????????1?0?1101?01????2???????????001?????????????????????????????????????????????????????????????????????????????????????????????????????????????????????????????????????????1?000???????????????????????????????????????????????????????????????????????????????????????????????????????????????????????????????????????????????????????????????????????????????????????????????????????????????????????????????????????????????????????????????????????????????????????????????????????????????????????????????????????????????????????????????????????????????????????????????????????????????????????????????????????????????????????????????????????????????????????????????????????????????????????????????????????????????????????????????????????????????????????????????????????????????????????????????????????????????????????????????????????????????????????????????????????

Chasmosaurus_belli 01001201011110111101000110011?01101001????0001100010010001010012001001001000110110000???0?0?111001101?011???2???110?0??110100000001021100?1?00002010010010??100100101000110001100100?1??00000011100011001041100111111111001221?111001000??????2???00??2?1?????21??201?0?00001?2?110110??????????????1??0??00001010111021010110?01101??1?102000001011?11??110211011111???00210000011000120000110100??00???????????????11??1111021????????3?????????????????????00001??1?102???????????????????????0011?0000010000001020101000000??00012?0110101?020?1?0??3[1 2 3]1001100001010?001?0111000000101???0???010000??001002100100?000[0 1]00021101?00100000000?1010100011110010101220010??1104??00?1000001110???0000000100101003121001020010?000100??1?1010111?1101??002101?110111011200?121011002101010011210?1000100??1011011211?1010010001000122111101?001111?0001001?0??0011201011010011000?11221??0000??????????????????????0?????000001000??01??????????1???1??????000?00?000?0????????????

Centrosaurus_apertus 0100120100111011111100011000[1 2]001102001?01100011000100100010100120010010010?01101100000?10100111001101?011???2??0110????1101010000010211100??0000201001001001100?10101000110001100100?11?000?00111000110010411001011111110012211111000000??????21000011101100002100201?0?0000112111011????20101111?1?11101?000010111110210?01?0?011?1??1?1001000010???1???1102110111?1???0011000001100012000111?1????00???????????????111?1111021220?0??132?1210???2?0??????1??000011011102110??0???????11122110200011?00000100000010000000000001000010?01001011?200100??321001100?01110101??01110000001011020???1100000?00100210??0000000010111[0 1]1?001?00?1000010101?001111011?0?1?2?010111104??00?10000011000000000000100001003121001021010?001100??0?101101101101??0121010110101011200?12101100210101001121000000100??11110?1?11?10??010???00?122?111011??1110?000100??1?0?0?0?????10?0?11?01?11221?10?000100110001?101??0110?10??0?00000010000?0100?1?01011100010000?1000?00?000?0????????????

Triceratops_horridus 0??01201011110111110000110011?0[0 1]101001?011000110101001000101[0 1]012[0 1]0100100100011011000001101001110011?1?0??11?2??0110??0?1101010000010211001??0[0 1]002010010010011001001010001100011000000111000101111000110010411000111111110012210111000000??????21??00012?11000121010012000000112111011100?20101111110111?10000010101110210?01???011011010101[1 2]100010??11?1?111211001111???0001?000010100120001???1????00???????????????1111111102122000011321121000020000????11000?0110111021100000?1110?1112212020???????????110?001?00??????000???0010?01?01010?2??????????????????????????????????????????????????????????????????????????????????????????????????????????????????????????????????????????????????????????1??????00?020?10?000100??01111011101201??0121012110101?11210?121[0 1]1200210??1001221110000100??1011??????????????????????????????????????????????????????????????????????????????????????????????????????????????????????????????????????????????00?0???????????????????

Hypsilophodon_foxii 000000100000200????0101100011?0101200101000110100001000001011001110110001000000110000120100110000010000100100100000?00?10001000000000?10000?000000100100000000012000111101000001010000000000?0100000220000001000100?????0000000000010100??????11011001001000002000000001111001001000??00?000001?00000101000000101?11100010?100?011010001000000?01010100111010110100011?000010110000000010001000100000500001100110000010101101[0 1]212000111102112101012?001011001010?010?0010011020010110111?????????1000000001000?00001001010?00001010010100100000020111100211001[0 1]001001101000101011000001000100????111100?0010[1 2]00100011000200000[0 1]112001001100001100010000011111[0 1]00101010010000?0?0???0000000000000000001001?00003?2?00?020000?000000??10010001001101??0021001100100?11201000101[0 1]0001???0000120010100002110?1010021021010110111001122011111100101110200101?0??00000010[0 1]00200000110112210100000100111101010110011?010110000001010000?10100100101100000000001000?00?000?0????????????

Gideonmantellia_amosanjuanae 0?????????????????????????????????????????????????????????????????????????????????????????????????????????????????????????????????????????????????????????????????????????????????????????????????????????????????????????????????????????????????????????????????????????????????????????????????????????????????????????????????????????????????????????????????????????????????????????????????????????????????????????????????????????????????????????????????????????????????????????????????????????????????????????????????001?100????0?0????????????????0?0??1???0??0?0?1000???00?0?0???????????????????????????????????????????????????????????????????????????????????????????????????????????????????????????????000000?????10001001101???021001100000?11?0101?????00???????????????????????0???10021021?1?110?????11220111121001?11?0??0?00?0??00?0?0??00020000?1??1?221?1000001001111??0??1?00?1????????0000001100??10100?001???00?00[0 1]0000?0???????????????????????

Convolosaurus_marri 00?0??1??000200????0101100111?010020010???000010010100?0010?10011??1000010?0000110000??10100?0001011001101100000000?00?11000000?01000?1000??01002010010000000?012?0011?1010000010101?00100?0?0?00???1?0??000101000??????000??00??00101?????????10???2110111?00??0??0010011?0?1????????????????????????????000010101000001001?0?0110????1000[0 1]000010????01?1001110100?1???????011??????0010?01?????0?0040000010111100011?1?11011212000111002112101?12010100100?1[2 3]0?010??0?02110200101??111?????????10???00001000??0011002010?00001?1?0????01?0?0???0???0??2??100??1?0??100?0100111100000?00?0?100?111010??0010210001001001000000011?00100000???1?000000011111111?01010100?01003?100110000?0001021100?0010000000[0 1]201?00??20100?010000???0??0?020011?1??0021001100100?11200?1010?000?1??00000020010?11002110?111002102101?0?0??????122?101121??1??0?0200101?100001010101002000000101?2???100000111110001010110011001???00000000100???1?1001001011000000000?1000?00?000?0????????????

Tenontosaurus_dossi 0000?0100001200????010111010200100210?0100?00010?0000000011110?1000?000??01011?11000002100001000101000?1??1?0?1?000?00??00?1000100000???0???0?00201?0000000001002000111?0100000112?1?0010000???1??001?0000101010000?????0000000?00010100??????11?0002120??10?02?00??00001?00???????????0??????????1?0?????00001010100100101101001?0100?20011000010?????1?1101110100?????000101?0100000010001?????0??01???????????????0011110?1212??0?1?002112?0???2??01??1??1120?010?00?02??0?00??1111?1?????????20???0000100??000?1???0?0?000010100???00?01?11?????0???210100????00??1010?0012010??101???1011011110100?0????????000100?0000000111001000?0??00?0?010000111111?00100?10010?0021100010?0??????0???0?0???????????1???000020100?0100?0??10000002001101??00210?1100100?11210?1??0??0001???000002011?011002110?2111?21021???1????????1?211?11?1001?????201????1??1000??2?????0??1???11?2????00000?1?1????????110???00?00???00000010??0?100??1??1011100?0[0 1]?00?1000?00?000?0????????????

Tenontosaurus_tilletti 000000100011200????010111010200000210101?0000010000000000111100100010000101011011000[0 1]021000010011010001111100110000?01?10011000100000?10000?010020100100000001012000110001001001020100010000?0100000120000001010000?????0000000100010100??????11?010212?11100020000001001100011010101110?0000010011001000000001010100100101101001101001200110000101010011110111010011??1000101101000000200010100000000???????????????011111011212000111012112100?1201010110011[1 2]0?010?00102110200101111?11112120102000000001001?00001002010?0000101001??011011110100?00??21010010100011101000012010001010000011011110100?001021000?0010?1010000011000100010000000001000011211110010001001011021100[0 1]101000011102100000[0 1]1100100012110000020100?010000??10000002001101??0021001100100111210010101000110?00000020000011002110?210112102101011?011001122110111100111010201101?1001000102?200100010011112210110000111110?01??01100110010010000000010000?100001101[0 1]11100000000?1000?00?000?0????????????

Iani_smithi 0?000010001?100????01??1?0101?0100??01?1?00000??????????0????0??????????????????????0??1000010001010000101?00?1?000?01?1001100010000??10?1010?00000001000000010020001000????????0?0111010000??100000220000001010000?????0000000100010?00??????1102??21001??0000?01??010001?????????????0??00001???110100??0000101011110010110??011????1200010000101011?11110111010???????0?101101000?00?????????????03?????1??1??0????111110112120001111?2??2100?120100[0 1]1100??20?010?001021102[0 1]01011?1?1?????????20?1??????00100000100201100000???001010[0 1]?01101??0??????2??100?01???????00?001101?????????0?????????????00002100??00?0010000?0????0????????????0?010001???111???101?100?????????????00?????????0?????????????????????????????????????????????????????????????????????????????????????????????????100211222??1121021010?1002?00?122110111100111?1020110??10?00?01?201?????????????????????????????????????????????????????????????????????????????????????00??0?000?0????????????

Ampelognathus_coheni 0???????????????????????????????????????????????????????????????????????????????????????????????????????????????????????????????????????????????????????????????????????????????????????????????????????????????????????????????????????????????????????????????????????????????????????????????????????????????1??????????1???01101001200000000101011?1?11111101???????????????????????????????????????????????????????????????????????????????????????????????????????????????????????????????????????????????????????????????????????????????????????????????????????????????????????????????????????????????????????????????????????????????????????????????????????????????????????????????????????????????????????????????????????????????????????????????????????????????????????????????????????????????????????????????????????????????????????????????????????????????????????????????????????????????????????????????????????????????????????????????????????????????

Burianosaurus_augustai 0???????????????????????????????????????????????????????????????????????????????????????????????????????????????????????????????????????????????????????????????????????????????????????????????????????????????????????????????????????????????????????????????????????????????????????????????????????????????????????????????????????????????????????????????????????????????????????????????????????????????????????????????????????????????????????????????????????????????????????????????????????????????????????????????????????????????????????????????????????????????????????????????????????????????????????????????????????????????????????????????????????????????????????????????????????????????????????????????????????????????????????????????????????????????????????????????????????????01210210?01100?01???????????1001101?0??1?01?101001000101????????????????????????????????????????????????????????????????????????????????????????????????????????????

Vegagete_Ornithopod 0??????????????????????????????????00??????????????????????????????????????????????????1?????????????????????????????????????????????????????????????????????????????????????????????????????????????????????????????????????????????????????????????????????????1?????????????????????????????????????????????????????????????0?1?????????1???0??0????1????????????????????????????????0???????????0????001110??1?2??011??011?12000?110?211??00?120[0 1]1111??0103???10??0???11021?10?1?101?????????????????????????????01??0????????????1??10??0??????1??????????????????????0????2?????????0??????????????????????????????0?0??10??0?2??????????1?0??0???????11101??0????????????????????????????????????????????????????????????0??????????????????????????????????????????????????????????????????????????????102101011?0110011221?011210011011??0??0??10000??21?1[0 1]0?1?0000111??221???0000?11?110?????1??1??????????0000?011??0?1?100?001011000?01000010???0??00??0????????????

Matheronodon_provincialis 0??????????????????????????????????????????0??0?????????0101?0120??000??1000000110?1??2?????????????????????????????????????????????????????????????????????????????????????????????????????????????????????????????????????????????????????????????????????????????????????????????????????????????1?????0?????????????????????????????????????????????????????????????????????????????????????????0?????????????????0111?011?020001110021?2101?12?1012110010????????0??21101201111???101??1??1????????????????????????????????????????????????????????????????????????????????????????????????????????????????????????????????????????????????????????????????????????????????????????????????????????????????????????????????????????????????????????????????????????????????????????????????????????????????????????????????????????????????????????????????????????????????????????????????????????????????????????????????????????????????????????????????????????????????

Mochlodon_suessi 0?????????????????????????????????????????????????????????????????????????????????????????????????????????????????0??????????????????????????????????????????????????????????????????????????????????????????????0?????????????????????????????????????????????????????????????????????????????????????????????01??????????????01101111000010000100011?1?11111111?0?????????????????????0???????????0?????????????????????10112120001110????2?0??120101111001?30?010?0010?110110101?0??????????????????????????????????????????????????????????????????????????????????????????????????????????????????????????????????????00?11??0??????????????????????????????????????1????????????????????????????????????????0??????????????????????????????????????????????????????????????????????????????????????????0???????????????????????????????????1??10???????????????????????????2??????????????????????????????????????????????????????????????????????0???0???????????????????

Mochlodon_vorosi 0???????????????????????????????????????????????????????????????????????????????????????????????????????????????????????00110?010???????????????????????????????????????????????????01000000?01??0002??????????????????????????????????????????????????????????????????????????????????????????????????????????01??????????????01101111000010000100011?1?11[0 1]1111100?1???????????????????????????????0?????????????????01[0 1]1?011?120001110??????01?120101111001?3??010?001021101101011???1?????????????????????1??00?000??????0?????00???00????0???????????11?????0000???????1?????????????????????????????????1????0???0?000000110?001?00010?????011000111111120?102010?????????????????????????????????????????????????????????????????????????????????????????????????????????????????????????????????????010210?1011010110001122111201100111010100001???????0???0??02000?001?1?210??0?????????????????????????????????????????????????????????????????????????????????????????

Obelignathus_septimanicus 0??????????????????????????????????????????????????????????????????????????????????????????????????????????????????????????????????????????????????????????????????????????????????????????????????????????????????????????????????????????????????????????????????????????????????????????????????????????????????????????????010????0??0?1??00101010???1110010100?????????????????????????????????0?????????????????0???101121200011????????????????????????30?????0?1?21??1?0?0?????1????????????????????????????????????????????????????????????????????????????????????????????????????????????????????????????????????????????????????????????????????????????????????????????????????????????????????????????????????????????????????????????????????????????????????????????????????????????????????????????????????????????????????????????????????????????????????????????????????????????????????????????????????????????????????????????????????????????????????????

Pareisactus_evrostos 0????????????????????????????????????????????????????????????????????????????????????????????????????????????????????????????????????????????????????????????????????????????????????????????????????????????????????????????????????????????????????????????????????????????????????????????????????????????????????????????????????????????????????????????????????????????????????????????????????????????????????????????????????????????????????????????????????????????????????????????????????????????????????????????????????????????????????????????????????????????????????????????????????????01010??0110??011110?0?1????????????????????????????????????????????????????????????????????????????????????????????????????????????????????????????????????????????????????????????????????????????????????????????????????????????????????????????????????????????????????????????????????????????????????????????????????????????????????????????????????????????????

Rhabdodon_priscus_type 0??????????????????????????????????????????????????????????????????????????????????????????????????????????????????????????????????????????????????????????????????????????????????????????????????????????????????????????????????????????????????????????????????????????????????????????????????????????????01??????????????011??0?1200?10000100011?1?110111010??????????????????????????????????0?????????????????0???10112?2000?1?????????????????????????0?0???0?1?21??11??0?1????????????????????????????????????????????????????????????????????????????????????????????????????????????????????????????????????????????????????????????????????????????????????????????????????????????????????????????????????????????????????????????????????????????????????????????????????????????????????????????????????????????????????????????????????????????????????????????????????????????????????????????????????????????????????????????????????????????????????????????

Rhabdodon_priscus 0??????????????????????????????????????????0??0?????????01?1?01??????00??00002?110?1??2?????????????????????????????????????????????????????????????????????????????????????????????01000000??1??0?0220?????101????????????????????????????????1??0?211011?0?02?0100011?00100?2020?1???????????????????????????01??????????????01101001200[0 1]10000100011?1?1111110100????????1011010000000???????0????0?????????????????0111101121200011100???2101?120101111001030?01??00102110112?01111?1???????????100??0??0010000010010?00?000??10010?012011100?00?????31020010020001??01?10[0 1]1[0 1]00000010??0?0????11010???00021100?[0 1]000011110[1 2]01[0 1]1?001000010?????0110011111110200121110??111?????????00????????0???????????????????00?0212010010000??10000001111201??0021000100101111?00001?01000??????001[0 1]210100[0 2]10021100[1 2]101021121010010?1000?12211121111?110??010[0 1]0[0 1]1?1000011?1101?020?10001111211?11?0001???????????1????1????????0000?0?0?00?100?????11??1[0 1]??00?????0???0??000?0????????????

Rhabdodon_Quarante 0????????0?1?00????00??1?01???00?2?001?????0?00?????????01011012??11000??000??0??001????????????????????????10????0????1?????????????????????????????????????????????????????????????1??000???10?0?021????????????????????????????????????????????????????????????????????????????????????????????????????00???01??????????????0110100120011000010101??1?1111110100?????????????????????002?????????00????????????????0??1101121200011??0???210???201?1111?0??20?01??0?102110110101111?1????????????????????????????????????????????????????????????????????????????????????????????????????????????????????????????????????????????????????????????????????????????????????????????????????????????????????????????????????????????????????????????????????????????????????????????????????????????????????????????????????????????????????????????????????????????????????????????????????????????????????????????????????????????????????????????????????????????????????????

Transylvanosaurus_platycephalus 0???????????????????????????????????????????????????????????????????????????????????????????????????????????001?000?01????????????????????????????????????????????????????????????????????????????????????????????????????????????????????????????1???101?01?02?00?00110010000201001??1?????????????????????????????????????????????????????????????????????????????????????????????????????????????????????????????????????????????????????????????????????????????????????????????????????????????????????????????????????????????????????????????????????????????????????????????????????????????????????????????????????????????????????????????????????????????????????????????????????????????????????????????????????????????????????????????????????????????????????????????????????????????????????????????????????????????????????????????????????????????????????????????????????????????????????????????????????????????????????????????????????????????????????????

Zalmoxes_robustus 0000?0100?00210????0001110101?0?022001010?0000??000?10?00101?002000?0000100000?110010?210000?00010??????00?01011000??1?1001?00110?001?1001??00000000010100??01002000110001000000011101010000??101000220000001010000?????0000000111000100???????102?12?1?11?0?0000?1?0110000001201001?01?????0???????1?????0?001010100000000101?01101001[1 2]0001000010[0 1]011?1?11[0 1]1110100?1????0?101101000?00000010???????00???????????????001?11011212000111002112?01?120101211001030?010?001021101201111?1?1??????????1?0?000?10010000000010???1100?0?0010?002?11001?01?????3111000002010??????10?0?10000?????0??????????????01[0 1]11000?1000?1000010101100?0?0?????????110001111110200112110???11????????????????????????????????????????0?0212111000010??11010011001201??01110011??101????????????????????0?01120000001000??1?11001[1 2]1021?100?0??????12211111111?11?1?0101101?10000[0 1]02110?0?20??0???11?211?10?00010?1100?1??01?0111?????????00??????????????????1??????????????0??00?000?0????????????

Zalmoxes_shqiperorum 000??0??????????????????????????????????????????????????????????????????????00?????1????????10???????????????011000??1?100?100110?00??1001?00?00???0010100??01002?0?1?01?1????????1??1010?00??10100?220??0?01?100?0?????000?0?0111000100????????????????????????????0?????????????????????????????????????0????0101?000000?????01101001200010000100011?1?111111010011????????1???0??????0???????????0????????????????001111011212000?110??1?2?0???201012110?1?30?010?00102110120111111?1???????????????????????????0???0??????????0??0?012????01????????310??0??0?00?1?????1????10????????0???????????????11201?0100?001010000[0 1]11?001?00010?????11100011?111???01?2110?????????????????????????????????????????????0?0202111010010??110100120?12?1??0121001110101?11201??1?????0?????01011201?0000[0 1]00??0?21?01[1 2]1021010[0 1]1001000112211011111?1[1 2]10?0101101?1?0001[0 1]2111000200100?011?2111[0 1]0?000?001?00?????1?01???????????00?????????1??0????????1????0?????00??0??000?0????????????

Iyuku_raathi 0??????????1??0????00??1?010???00?11?101??00?010????????0????00????000001010??011000??2??????0???0?????????001100?0?01??0100?0000?00???000??0?????0001010?0?0??????0110????????????????1?0??11???0??220???00??????????????????????????0????????????1?????110??????????????????????????????????????????????00????1??????????????0110100?10001000010101001?101001010????????0101000?000001000??????0??00????????????????11110011212000111001122100??21000????11?30?010?00?021002001011???1???????????????????????????0001???????0???001010???0?????00?????????????0??????????1?????????????????????????????11121100100?00000010010??0????????01???001?02?0?11112101?00????01?????????????????????????????????????????00020?00?010010??110001110?1111??00210??010?00111211101101?0001?10000012110??20002110??110011121010010001001122011212101121010[1 2]00110?1001000112010020010011?11?010101000?00111001010??????????????00000010?0???0??0???0???????0??????0?0?0???????????????????

Kangnasaurus_coetzeei 0??????????????????????????????????????????????????????????????????????????????????????????????????????????????????????????????????????????????????????????????????????????????????????????????????????????????????????????????????????????????????????????????????????????????????????????????????????????????????????????????????????????????????????????????????????????????????????????????????????????????????????11??01??120000111????????????????????1?????11??????110?1010?1?1?????????????????????????????1??2???????????????1??????0?????????????????????????????1????2?????1?????????????????????????????????????????????????????????????????????????????????????????????????????????????????????????????????????????????????????????????????????????????????????????????????????????????????????00210210100100100011222101111011211102?0?1??1001001112121?20011011011221??????0?101120?????11111?????01???000??????10???10?10?0?1????0????010???????????????????????

Gasparinisaura_cincosaltensis 00?0?0??0??????????0??????????????????????????10??????0?01011002000??0001???10?1100000?1110010000?1000000??00?0??00????10000000?0?000?0000??00001000011?000?00012000110101000001011000010010?11??0?0??00?0?0??00?00?????0?00??0100010000???????101??010??000?0?10???020?0?????????????????0???????????????0000?????????????????0110????2000100?010???????1?0?11000??1???00000100000000020001????????0????????????????00?1110102120000111111121011?2?011000111?20?010??010?10????1011???1???????????0?0??0010?00000000021?0??100????00??00??0?0?0???1?0??21000??00?00110??001010110000?11??001111?110100????0????0?0000??100120102?001000?0??????00000011?001?210?0000??????????????????????00200?????????1???1?????00020?00?000000??1?00000200111???00210?110010011120100110100001?1??0000200100?000211011?1?0210210100100101011221111101011210?0200100?0??00101100010201110010112210??00001?0112001??01111?101101???00001010?0111011011011??011?0[0 1]0000?000?00?000?0????????????

Notohypsilophodon_comodorensis 0????????????????????????????????????????????????????????????????????????????????????????????????????????????????????????????????????????????????????????????????????????????????????????????????????????????????????????????????????????????????????????????????????????????????????????????????????????????????????????????????????????????????????????????????????????????????????????????????????????????????????????????????????????????????????????????????????????????????????????????????????????????10?00000021?00?100????00??00200?0?????????????0000?0?0????????1??????????????0???????????????????????0???0??00100?11?001000?0???1??001002001011121010100???????????????????????????????????????????????????????????????????????????????????????????????????????????????????????????????????????0??1?2101011000110112211011210112111????????1?0?0?011?10102001?001?11201010?0001???1?????10111111????????????????????????????????0??000?????0???????????????????????

Anabisetia_saldiviai 0???????????????????????????????????????????????????????????????????????1???????1?????????????????????????????????????????????????????????????????????????????????????????????????????????????????????????????????????????????????????????????????????????????0?0???0?01011010?????????????????????????????????????????????????0?1????01???1?0?0101010?1?10?0?????0?????????????????????????????????0?????????????????????1011212000[0 1][0 1]1[0 1]???12?01[0 1]12?[0 1]11?000?10?0??10?00??21002?0??11????????????????????????100100000021?100110?????????????????????????????????????????????0??22?11??10??0???????1?????0?101000??00?00020?120111?001?0010????0000100200111112101010?0?1000????????0000?0001021000000????100?????0001120?00?01101???1?000101001111??0021012000101?11200111?01100010?000000200100100021?0021100210210100100[0 1]1101122111[1 2]11101121110200110?100000011211102???1001?11201010?00[0 1]11?11??01?111?111100?0????001010111111101101101011001000?0??10???0??000?0????????????

Weewarrasaurus_pobeni 0???????????????????????????????????????????????????????????????????????????????????????????????????????????????????????????????????????????????????????????????????????????????????????????????????????????????????????????????????????????????????????????????????????????????????????????????????????????????1??????????????0?1?????10?00000??0???0?1???????0????????????????????????????????????0?????????????????11???0112120000110?????????????????????????01??00??21002001011?1??????????????????????????????????????????????????????????????????????????????????????????????????????????????????????????????????????????????????????????????????????????????????????????????????????????????????????????????????????????????????????????????????????????????????????????????????????????????????????????????????????????????????????????????????????????????????????????????????????????????????????????????????????????????????????????????????????????????????????????

Atlascopcosaurus_loadsi 0????????????????????????????????????????????????????????1???00????0??0?1???1??110?0??2?????????????????????????????????????????????????????????????????????????????????????????????????????????????????????????????????????????????????????????????????????????????????????????????????????????????????????????????????????????????????????????????????????????????????????????????????????????????0?????????????????1111?1112120100111221?2101112?0111101110?????0?0??????????????????????????????????????????????????????????????????????????????????????????????????????????????????????????????????????????????????????????????????????????????????????????????????????????????????????????????????????????????????????????????????????????????????????????????????????????????????????????????????????????????????????????????????????????????????????????????????????????????????????????????????????????????????????????????????????????????????????????????????????????

Diluvicursor_pickeringi 0?????????????????????????????????????????????????????????????????????????????????????????????????????????????????????????????????????????????????????????????????????????????????????????????????????????????????????????????????????????????????????????????????????????????????????????????????????????????????????????????????????????????????????????????????????????????????????????????????????????????????????????????????????????????????????????????????????????????????????????????????????????????????????????????????????????????????????????????????????01?0?1010221000011000010001?0?00????????????????????????????????????????????????????????????????????????????????????????????????????????????????????????????????????????????????????????????????????????????????????????????????????????????????????????????????????????????????????????????????????????1?1201???1000111112001011111?1100?011100000?0111111[0 1]?11011010110011?[0 1]???01????????????????????????

Fostoria_dhimbangunmal 0???????????????????????????????????????????????????????????????????????????????????????????????????????????101?000??????????????????????????????????????????????????????????????????????0????10?0?0210?0?1?1????00?????????????????????????????????0?????0????????????????????????????????????????????????0??????????????????????????????????????????????????????????????????????????????????????????????????????????????????????????????????????????????????????????????????????????????????????????????????????????????????????001??01101?????0?????????????????????????0????2??????00?????????????????????????00?00020011111??0?????????????????01?0??1?????????????01?????????????????????????????????????????????????????????????????????????????????????????????????????0??????0????????001012110?1???????????????????????????????????????????0??1001?0021201????????????1201010??0???????????????????????????????????????0???????????1??0?[0 1]?0???????????????????????????

Galleonosaurus_dorisae 0?????????????????????????????????????????????0??????????1???00????0?00?1?001?111000?01?????????????????????????????????????????????????????????????????????????????????????????????????????????????????????????????????????????????????????????????????????????????????????????????????????????????????0???????????????????????????????????????????????????????????????????????????????????????????0?????????????????11?1?1112120100111221?2101112?0110001110?????0?0??????????????????????????????????????????????????????????????????????????????????????????????????????????????????????????????????????????????????????????????????????????????????????????????????????????????????????????????????????????????????????????????????????????????????????????????????????????????????????????????????????????????????????????????????????????????????????????????????????????????????????????????????????????????????????????????????????????????????????????????????????????

Leaellynasaura_amicagraphica 0???0?????????????????????????????????????????0??????????1??100?0100?00110001?011000001?1?0110000???0??????0????????????????0?????0001?000?10000000?01010001?001200011??010?010?121010000010?1?000001?????????????????????????????????????????????????????????????????????????????????????0?00??0???01????000???????????????????????????????????????1???????????????????????????????????????????????0?????????????????1111?1112120000111221?2101112?0110001110?????0?0???????????????????????????????????????????????????????????????????????????????????????????????????????????????????????????????????????????????????????????????????????????????????????????????????????????????????????????????????????????????????????????????????????????????????????????????????????????????????????????????????????????????????????????????????????????????????????????????????????????????????????????????????????????????????????????????????????????????????0??????????????????????

Muttaburrasaurus_langdoni 0???0?0????0????????????????3?0??????????????????10??0?0???1??1????00?0????1120110?00?2?0????0??10???????1?110100?0????1???1000??0001?0001??00000000010100000??02000111001000?00?21?????00??1110?0?0210000001000100??????0?0?0????????????????0?????0?1??10?002?0???010111?0??1???????????????????????0????00?????????????????????????????????????????????00?????00?1???????01001?00?0010001????????0????????????????0111?11112120000111321?2?011?2?011[0 1]001110????????????100?1???11?1???????????1?0000??0?0????0001??[1 2]????????00[1 2]000??011?1?1??????????2??????????????????????????0???????????????????????1110???0???0020?111101?0????0?????1??10100100?11??20010011001010021?0011010??????????0???????0?????3?3??0?02?001?000010?????0?1120?1201??0120002010101?11??0?11?0?1?0??????00???????????????????010210210100100111011221111111011011102001?1?100100011101002?0??011?1?201010?000111112001??1111??1???001000000?01???0?00??0?10????10?????0?0??????0?000?0????????????

Qantassaurus_intrepidus 0???????????????????????????????????????????????????????????????????????????????????????????????????????????????????????????????????????????????????????????????????????????????????????????????????????????????????????????????????????????????????????????????????????????????????????????????????????????????1??????????????01101000100000000101110?1?10001101?0??????????????????????????????0??0?????????????????111111112120000111??????????????????????20?010?001021012101011?1??????????????????????????????????????????????????????????????????????????????????????????????????????????????????????????????????????????????????????????????????????????????????????????????????????????????????????????????????????????????????????????????????????????????????????????????????????????????????????????????????????????????????????????????????????????????????????????????????????????????????????????????????????????????????????????????????????????????????????????

Trinisaura_santamartaensis 0????????????????????????????????????????????????????????????????????????????????????????????????????????????????????????????????????????????????????????????????????????????????????????????????????????????????????????????????????????????????????????????????????????????????????????????????????????????????????????????????????????????????????????????????????????????????????????????????????????????????????????????????????????????????????????????????????????????????????????????????????????????????????????????????????????2??????????????????????????????????0?12???00?10????1?1?????????002?1?01???01000200111101?10??00?00????000??0200001???0?????????????????????00?????????????????????????????0?020?01?011010??1?0100120?1111??00210?0000101111[1 2]?10?1101100011??0001020000000002111?21?002??21????????????122?11211?011????02?01?0?10000001??10?????????????2?1?????????????????????????????????????????????1?1?????????0????0?0???????????????????????????

Isasicursor_santacrucensis 0???????????????????????????????????????????????????????0????0????????0???????011???????????????????????????????????????????????????????????????????????????????????????????????????????????????????????????????????????????????????????????????????????????????????????????????????????????????????????????????????????????????????????????????????????????????????????????????????????????????????0?????????????????1????0112120000111????2101102?01100011??????????????????????????????????????????????????????????2???????1?????0????2???0??????????2???????1??????????1????2?????1?000????????????????????????????0?001?0??????????????????0???02?0????1???1?10??0??????????????????????????????????????????????????????????????????????????????????????????????????????1?????????????????????????????????1?2????????????1122?10?1110112?110????0??0??1?00112001020001000111201??????????????????????????????????00???????0?[0 1]?1???1?1???1???00?????0???????????????????????

Macrogryphosaurus_gondwanicus 0????????????????????????????????????????????????????????????????????????????????????????????????????????????????????????????????????????????????????????????????????????????????????????????????????????????????????????????????????????????????????????????????????????????????????????????????????????????????????????????????????????????????????????????????????????????????????????????????????????????????????????????????????????????????????????????????????????????????????????????????21???0????0110111100022?100001??10012?012001000?11?11112??001001?0011?????1????2?00??1???0?1111?11????????????????????????????????????????11??????????????????????????????????????????????????????????????????????0?020?00?0?0000??110?0??20?1111??0021001010100?112100111010000111100000200?0020?0???1?21??????????????????????????????????????????????????????????????????????????????????????????????????????????????????????????????????????????????????0?000??????????????

Mahuidacursor_lipanglef 0?????????????????????????????????????????????????????????????????????????????????????????????????????????????????????????????????????????????????????????????????????????????????????????????????????????????????????????????????????????????????????????????????????????????????????????????????????????????????????????????????????????????????????????????????????????????????????????????????????????????????????????????????????????????????????????????????????????????????????????????????1?????????110111100022??0000110?0000?00200?0??011?0101?????????????????????????????????????????1????0?001111000?000000200110101?111000?0?11?0?00100200111111101011100101?0?0?0?0?000?00??10200??0???0????????????????????????????????????????????????????????????????????????????????????????????????????????????????????????????????????????????????????????????????????????????????????????????????????????????????????????????????????????????????????????000?0????????????

Morrosaurus_antarcticus 0??????????????????????????????????????????????????????????????????????????????????????????????????????????????????????????????????????????????????????????????????????????????????????????????????????????????????????????????????????????????????????????????????????????????????????????????????????????????????????????????????????????????????????????????????????????????????????????????????????????????????????????????????????????????????????????????????????????????????????????????????????????????????????????????????????????????????????????????????????????????????????????????????????????????????????????????????????????????????????????????????????????????????????????????????????????????????????????????????????????????????????????????????????????????????????????????????????????????1021010110010001122210111101121110???????1001???11201??20??????011?0??101??????????????????????110????0000??????0?[0 1]?110?101???0??????????????????????????????????

Sektensaurus_sanjuanboscoi 0??????????????????????????????????????????????????????????????????????????????????????????????????????????????????????????????????????????????????????????????????????????????????????????????????????????????????????????????????????????????????????????????????????????????????????????????????????????????????????????????????????????????????????????????????????????????????????????????????????????????????????????????????????????????????????????????????????????????????????????????????????????????????????????????????0???012???0????????????????0?1??????????1??????????1???0?????????????????????????????????????????????????????0010020?????1210??????????????????????????????????????????????????????????????00?0???????????????1??00210?1010100???????????????????????????????????2????2?????1?2?????????????1221?01111??1??1?????????????????????????????????????010??00????????????111111?????????00?????????1??????????????????????????????????????????????

Talenkauen_santacrucensis 00???????0?1?00????000?1?0111?00001101010?000010000010?0?1???00?0??0000?1100110110?????????????????????????????????????????????????????????????????????????????????????????????????????????????????????????????????????????????????????????????????????????????????????????????????????0????001?00???10???00??101011110000110110110100020000?000101010?1?1000110100?1????0?101011100?00?0??1?????0??02???11?11?3????0?11111[0 1]112120000111221121011020011000111?10?010?00??21001101011?100?????????11???01001011011110002211000011010010?01?000000?111?1102???01??1??????????1????????????0???1?11?1101?0?10011101010010002000101111002100000????0001?020011111210???????101??????????????????????????????????????????0020?00?01001???1?0000020111?1??0021001000100?1121001?????00?1??1??????????????????1?2110021?2?????????????1221111111011??1102001?????????0?????00200?100100120101010001111110010111111110110110000?0?010??????100?1?101?1??00000??1?00?00?000?0????????????

Callovosaurus_leedsi 0???????????????????????????????????????????????????????????????????????????????????????????????????????????????????????????????????????????????????????????????????????????????????????????????????????????????????????????????????????????????????????????????????????????????????????????????????????????????????????????????????????????????????????????????????????????????????????????????????????????????????????????????????????????????????????????????????????????????????????????????????????????????????????????????????????????????????????????????????????????????????????????????????????????????????????????????????????????????????????????????????????????????????????????????????????????????????????????????????????????????????????????????????????????????????????????????????????????0011021010110001001122110112100111110200110?100100001?0?????????????????????????????????????????????????????????????????????????????????????????????????????????????

Dryosaurus_altus 0?????1?????2?0????001??00?02???????01?????0??0????0????0?????????????0??0?0000110????0???0????00????????0?0000?000?????01??000?0?000?000010000000000?01000100?02100?1?1010???00?211?001000011?01000??????????????0?????0?0???0?100??100?????????00?????1???001?0?0?0101?10001101??0??????????????????????00??0????????????????01101??01000100?010101??1?10?0110100?1????0??00?00??0?00?0001????????00????????????????1111?[0 1]11212000111021112101?121001011011010?01??????21002010?1101?100201111111?????????1?0100010011?0?0?001?10000?00??010011?0?00??2??1000?????01?0??0001?22001??1100000??????00?0?0011121???101000010100111100200000?00100101001101101110010000001000????????0000000010000??0001?0010001????000020000?010000??12000112001111??0021001100100111200110101000011?11000020010010002111?2110021021010010001001122011212100111110200110?1011000012120020101011001221000?00011111200101111011100101100000000110010[0 1]011011010110010000001100??00?000?0????????????

Dryosaurus_elderae 0??0001?0?11200????001?100?020000021010100000010??0100000101000210100000101000011010000011011000001000?010100000010?01?10100000100000100001000000000?1010?0100?0?1001101010???0012110001000011001000210010001000000?????0000000110010100??????111?0001201?10?00?010000011?00111010001100?00000???01?010?0?000000101111001?0101101101??01000100001010?0?1?1000110100?1????00100000100000100010????0??00???????????????011110[0 1]112120001110[1 2]111210??1210010110?1010?01???01021002010?1101?1??????????10?000001?1??100???????0?0?0???1?00???1?001001100?00??21?100000?0001?????????????????????????????0??0?111112100?0010001?010011?10010???0?0010010??01?01?01??00???????????0?0?0???000000001???000?0010?10??????21?0?020000?010000??1200010[1 2]001111??0021001100101?1120010010??00?????????????1???????????????0?1021?????0?????1122?112121??1???102??????101?0?0????????????????????????????????????????????????????????????????10?01????0?0??????????????00?00?000?0????????????

Dysalotosaurus_lettowvorbecki 0?10?0100011200????001?100002?0000110101000000100000?0?0010110020010000010100?0110100?00110110?000100001?0100000000??0?100?0000101000?0000100000?00001010001000021001100?1??????12111001000011001001220000001000000?????00000?0110010100??????11100?011011100010010000011110011010001100????00??00??01?0??00?00010111100110101101101000100000000101010?1?1010110100?1???000100?001000001000??????0??00???????????????011110[0 1]11212000111021112101??21001011011020?010?001021002010?1101?1?????????100000010?01[0 1]0100?1001110?01001?10000101??01001?0??0???211?0000000011??00100112201100???0?0?????110??0?011111000?000000010100111?002000?0?001?0101001101101110010101001000?????????????0??????????????????????????00020100?010000??12000102001111??0021001100100?1120?10010100001???1000020010010002111?2110011[0 1]21010010011001122111212100111110200110?10[0 1]1000012020020101011?012?1?10?000111112001?10110111?1?0110000000011??10[0 1]01111101?110???0000?11000?00?000?0????????????

Elrhazosaurus_nigeriensis 0???????????????????????????????????????????????????????????????????????????????????????????????????????????????????????????????????????????????????????????????????????????????????????????????????????????????????????????????????????????????????????????????????????????????????????????????????????????????????????????????????????????????????????????????????????????????????????????????????????????????????????????????????????????????????????????????????????????????????????????????????????????????????????????????????????????????????????????????????????????????????????????????????????????????????????????????????????????????????????????????????????????????????????????????????????????????????????????????????????????????????????????????????????????????????????????????????????????0021021011110010001122110212100111110200110?111000001?02????????????????????????????????????????????????????????????????????????????????????????????????????????????

Eousdryosaurus_nanohallucis 0???????????????????????????????????????????????????????????????????????????????????????????????????????????????????????????????????????????????????????????????????????????????????????????????????????????????????????????????????????????????????????????????????????????????????????????????????????????????????????????????????????????????????????????????????????????????????????????????????????????????????????????????????????????????????????????????????????????????????????????????????????????????????????????????????????????????????????????0????????1???0?0011220010?????00?????11000??????????????????????????????????????????????????????????????????????????????????????????????????????????????002??00?011000??11?0010200111???002100?111100??????????????????????????????????????????1001102101111001110112201121211?1211102?0?10?100???0????2?010001001011221010?00011111200101011011100101100000000110?1000100110101101100[0 1]000110???????????????????????

Valdosaurus_canaliculatus 0?????????????????????????????????????????????????????????????????????????????????????????????????????????????????????????????????????????????????????????????????????????????????????????????????????????????????????????????????????????????????????????????????????????????????????????????????????????????????????????????????????????????????????????????????????????????????????????????????????????????????????????????????????????????????????????????????????????????????????????????????????????????0????10021??0???0???000??01200?0???????????11?????0?????01?0?101122001101100011011?11010??????????????????????????1??????????????????????????????????????????????????????????????????????????????????01?2??1??010?????1?000101001111??00210001?0100?11200?00101100?1?1?1?0??2????0??0021111211002102101[0 1]110010001122111212100111110200100?11110000120[1 2]1011[0 1]1100?01122??10000011?112001?101101110[0 1]10????000001????10[0 1]011111011??0???00?0?0?0????0?000?0????????????

'CM_1949' 0????????????????????????????????????????????????????????????????????????????????????????????????????????????????????????????????????????????????????????????????????????????????????????????????????????????????????????????????????????????????????????????????????????????????????????????????????????????????????????????????????????????????????????????????????????????????????????????????????????????????????????????????????????????????????????????????????????????????????????????????????????????????????????????????????????????????????????????????????100?0??01011???0010??110?????0?00?????????????????????????????????????????????????????????????????????????????????????????????????????????????01020000?010010??11000002001101??012101?1??101??????????????????????????????????????????100110210??0100?????122111212???1?10?0200110?10110011121?1010?10011?112?1????000?0?11200???0?????????????????????????????????????????????????????????????????????????

Camptosaurus_dispar 001000100011200????000?10000200010210101?000001?00000000010100020000?0001010001110000000110110000010000100101011000?01?10001000000000?00?0010000000001010001000020001?0101000000111?0000001011?11000210000001000100?????0000000100001100??????110?1021101110000?1000010001101120200001?00???0???????0?????00001010110100111101?01101000[2 3]001[1 2]000010101?01?1101110100?1???001000000100000200010?0??0??00???????????????011110111212000111021122100?121011101?11110?01??00102101201101111?10020121111100?000010?00000?100?10000000102001010010111110?0100??3111[0 1]0[0 1]0010001?000100111100?0010001110101101000?0011110000001001110100112?00100110?001001010001011[0 1][0 1]1010101111011?0121100101100111000000000011110100013121001020?00?010010??110100[0 1][1 2]0011?[0 1]??012100[0 1]1[0 1]0101111210000101100010111000020[0 1]00010102111121011110210101100010?11221101121001??110201101?10110[0 1]111?120020??00011112110110000111112001?10100??101000?0000000011000?[0 1]001000011??1010000000?000?00?000?0????????????

Cumnoria_prestwichii 0?????1?????????????0??1?000??00?02?01?????0001?00??0??00????0????0??00?????0?0110??0?0??????0??0???????????101?000????100??????00?????00??1??????????????????????00??????????????????????????????????0???001000?00????????????????????????????1????2?1?????????1???00000100?120????00????????????????????0????0?????????????????1????0????1?0?01010?0???1???11??0?????????000000????0??????????????00????????????????111?11112120001110?1?22100??21011001?1?1?0??10?0???210?2011011???1?????????21????????0??0????10010???0??0???001?10000??1??????????211?????0??0?100???10??11??0??1?00????????????0?001111000?0??????1010?1011?010?1100?????101101101100101010100?1??10?????????????????????????????????????????????????0?00?0???0?00011001101??0121002100001?1?????1??01??????11?10?02001????00???112?????1021010110011001122011112100121110???????1011??1012010?200100010112??01100001011120010101?0?1101???????000?0100?0????00?00?1??1????0?????0????0??????????????????

Draconyx_loureiroi 0???????????????????????????????????????????????????????????????????????????????????????????????????????????????????????????????????????????????????????????????????????????????????????????????????????????????????????????????????????????????????????????????????????????????????????????????????????????????????????????????????????????????????????????????????????????????????????????????????0???????????????????????1?21?????110?????1????2?011011?????????0???????????????1???1???????????????????????????????????????????????????????????????????????????????????0?1????????????1?0?????????????????????????????????????????????????????????????????1????????????0?1?0?1??????????????0??????????0??????0??????????????????????????????????????????????????????????????????????????????????????????0??????????????????????????????????????????1010?0?011010020110000?11201001?000101111????111?0?110?00????0000?0110011[0 1]?0??1??11??111?0000???0???????????????????????

Emiliasaura_alessandrii 0?????????????????????????????????????????????????????????????????????????????????????????????????????????????????????????????????????????????????????????????????????????????????????????????????????????????????????????????????????????????????????????????????????????????????????????????????????????????????????????????????????????????????????????????????????????????????????????????????????????????????????????????????????????????????????????????????????????????????????????????????????????????????????????????????0010??010????????????????01?00????????0??001?12111???0??1?0??????0???????????????0?00?1001?1??110110?0?0?????01?10?11000???100???????????????????????????????????????????????????0??2??0???000????010??0010?1111??002100????101?11?1000???????1???0?????????????002?11?2?0101102101??10??????122?111111??11?1?010110??11110110?211??2010???1?11211??0?00010?1100?10101?0111?01?????00?001????0??001?0101???1???0[0 1]000??0??????000??????????????

Oblitosaurus_bunnueli 0??????????????????????????????????????????????????????????????????????????????????????????????????????????????????????????????????????????????????????????????????????????????????????????????????????????????????????????????????????????????????????????????????????????????????????????????????????????????????????????????????????????????????????????????????????????????????????????????????????????????????????11??111?120001110??????????????????????????????0??210020110110??1???????????????????????????????????????????????????????????????????????????????????????????????????????????????????????????????????????????????????????????????????????????????????????????????????????????????1???????????????????????????????????????????????????????????????????????????????????????????????????011110???10?10??11011222111121????1110??1????1010?01?1?1?1020100001?11221001?00011111?0010111?0?11??????????????????????????001???1???0??????0???????????????????????

Owenodon_hoggii 0??????????????????????????????????????????????????????????????????????????????????????????????????????????????????????????????????????????????????????????????????????????????????????????????????????????????????????????????????????????????????????????????????????????????????????????????????????????????????????????????01101000200100000100010?1?1111110100?????????????????????0????????0????????????????????111111112120001110??????????????????????10?010?001021012010?1111??????????????????????????????????????????????????????????????????????????????????????????????????????????????????????????????????????????????????????????????????????????????????????????????????????????????????????????????????????????????????????????????????????????????????????????????????????????????????????????????????????????????????????????????????????????????????????????????????????????????????????????????????????????????????????????????????????0???????????????????

Uteodon_aphanoecetes 0???????????????????????????????????????????????????????????????????????????????????????????????????????????????????????????????????0?0??0??0000?00???0?0?00?0??????????????????????????????????????????????????????????????????????????????????????????????????????????????????????????????????????????????????1??????????????01?01000300021?0010?01??1?110111?100?1????0??????????????0???????????0?????????????????11?1?11?2120001110??????????????????????10?0???0?1021012011011?????????????11????0?01000?1000[0 1]0020?0?00001020010?012001111?101?0??211000?00?0001?00??0011210001010??0??????1010?0?0010100101001001110110002100100010?000001010011011001110101010?1110121100111100111000100000011110100?1312100?020?00?000010??12010012001101??0121002110100111200010101100?1?1?100002000002000211112101011021010110001001122110[1 2]12100111110201101?1011001112020020100001111221??10000?01111001011??????????????00000?????0?[0 1]?0?0?001??????????????????00?000?0????????????

Hippodraco_scutodens 0??0??0?????????????????????200???????????????????0000?001011012000000001???120110000??0010010?000???????1200011000??1?10001000001000?0000??0000000001110000000?2000110?01000100121000110000???11000??000000101??00?????00000001000?0100????????0????11??1101?????????00?000????????????????00????????????0000?????????????????0?10??????002?0?010???0???11?1110100?1???001000100?00000200010????0??0?????????????????1??1?11?212???11?0??1?21????21001?010???????1??????2101?01?????????????????????????????10?00?10010?000??????0010?01??11?1??00????????00110?100?1???????????????????????????1?1????0111110???00?10021111110??0????????01????01000101111?00????????????????????????????????????????????????????????????????????????????????????????????????????????1????1??0??????000020000?????211??????????2?0???????????1?2?11???1??1????0???????????????????????????????1201?????00111111001010110011?11?????0000??????0???0??1?011??2???10000??00??0??000?0????????????

Iguanodon_bernissartensis 000000110011200????000111010310010211101?0000010000000000101101200010100101111?110110000010020100011000001101011000?01?10001000000011010?0210000000?010100000000200001000100010012100011000011111000110000001010100?????0000000100010100??????21?0012120111010?010?01200000011202001?0?0?2?00010?01001000?00001010100100111101101101000310120000101110?11111111011111??1000100100100000100010000000000???????????????111111111212100111031122111?121001011011100?010?000021012010?1111?11120111012000001111001000001001110011001020010?012011110200100??3102011012001101001001211000101000010???1111000?0011000000000100200111112100111110?01010?010001011111210112100111?014100011110011100110000011?211101112131001021000?000020??11001011001101??0121000100101011200?201011001101000000200?0010002111121011210210??[0 1]100??0?112211?1111001??0?0101101?111101000?0?0021??1???111211?110000111112001?1011001110001100000000110?0?100?001011??211?100000?000?00?000?0????????????

Mantellisaurus_atherfieldensis 000000110011200????0001110103[0 1]0010211101000000100000[0 1]0000101[0 1]01200010000[0 1]011120110110001010020100010000000201011000?01?10101000000011?10002100000000010100000000210011000100010012100001000011101000210000001010100?????0000000100010100??????21?0112?20?1101?0010201000000011212???1????200???????00?????00001010110100?11101100101000310110000101111?11111111011111??000010[0 1]100100000100010000000000???????????????011111111212100111031122111?121001011011100?010?000021012010?11?1?1?????????210000111100[0 1]010001001[0 1]100[0 1]1001020010?01201011?200100??310211101100110100100121100010100?110???1111000?01110[0 1]000?000100200001102000111010?01[0 1]111010001011111200112?00?[0 1]1?001100011110011100110000010?21110111313100102[0 1]000?0[0 1]0010??10000011001101??0121001100101011200?20101100110100000?2000002000211112100[0 1]21020010[0 1]10010101122110111100111010101101?111100100212002111110101122101110001111120010101100110000110000000011000?1001001011??211?100000?000?00?000?0????????????

Ouranosaurus_nigeriensis 000000100011200????000011010200010211101?00000??0100000001010012000100?000111201101100?1010020100010000111201011000?01?1000100000000100000210000000001010000000020001101010001001210101000001111100?210000001010100?????0000000?0?0?0100??????11000121201110112?100000000000112120110000????00???0??01????00001010010100111000?0010100031012100010111001?1111110?1011??0000101100100000100010????01000???????????????01111?111212100111031?221?1?12100101101?100?010?0?102101?010?1111?1?????????21000011[0 1]10010100?1001110011001?10010?00201101020?1?0??2002[0 1]0101?00?101011101211000101010120???0[0 1]0?000?00110001000000002111111021[0 1]01?10?0?01001?01000101111100010210011000141?00111100111001100000?0??110?11?30?1001021000?000020??11000012001101??0121000100100011210020101[0 1]00110100000021[0 1]0002000211102100121120010[0 1]10010001122010111100101110101100?1111001002010021111001011221[0 1]101000111112001010110111????????000001????0?1001001011??2???1000?0?000?00?000?0????????????

Altirhinus_kurzanovi 00?0?0010011200????0001110002100102101?1?0000010010100?01??1?012????0?0??001120110110?010100201000100000002?1011000?01?1000100000?011?00002101000000010100000000200001000100010012101011001011?11??????0???01?????0???????0??001?0010100???????????1???????????????????????????????????0??0?00?0?0??11??00000010101101001011011011010003101200?0101?1??111112110011?1??100010110010000010001??0???0?00???????????????011111111212100111031122111??21001011011?00?010?100021012010?11???1???????????0?0????????????????????????????????????????????????????????????????01???001111???0010??0??????????0??011100000?001100200001112?001010?0?01?1??01000101111100?112?00?00?0011?00?1?10?????01100000?0?21100111313100?020?00?010020??20000111001101??0?21000110101?1120002010110011010000??2?00?020002111??1??0????????????????????????????????????????????????????????2????????1?2??????00????????????????????????????000???????????????01?????????0?0???00?0???????????????????

Probactrosaurus_gobiensis 000000110011200????000111000??00?0210101???000100????0001??1??12????0?0????1120110?1?????????0????????????????1?000??1?100??000000??1???0??100000?0001?100????002?000?00???????0?2100011000011111??0110000001010000?????00000001000?0100???????1???1??????????2??000???????0?1?12?????????????????????????000010101?010011??0??01101000310110000101111?1?1112110111?1???000100100????00?0??1????????00????????????????1111111121220011103112211??121001011011?00?010?100021012010?1111?1??????????0?????????????0??1??1???????0??2?0?????10??1??????????301???101?0011??????01211000??100?0?????????????011101000?0001?0?0000111??001110?0?01011?010001011111000102?00?00?0?????????100??1001??0000???21?0?1??????001021000?010010??1?000111001101??0121001100101011210120101?00110100000121100010002111121010210200??110?????11221101111001??1?0101101?1111001?0?1???21??1???011221?1??00??1?111001?????????????????00000?????0?110?00?011??211?10000??000?00?000?0????????????

Hadrosaurus_foulkii 0??????????????????????????????????????????????????????????????????????????????110??????????????????????????????????????????????????????????????????????????????????????????????????????????????????????????????????????????????????????????????????????????????????????????????????????????????????????????????????????????????????????????????????????????????????????????????????????????????????0?????????????????11???111212200101031?22111?1210000???11??????0?10??21000000?1111?1???????????????????????????1001???????0???001??0?10????????????????????????????????001?10?000?1?0??????????????????????????????????????????????0??0????11010001011111000100?0010000????????????????????????????????????????0?020000?0?0010????000?010?1201??0121000110101??????????0??????????000?2?0??0000021?11?1001210?0010?100000001221100?01001011?0101?10?1111001001100021111001011221?11100???????????????????????????000?0?????0?11???1101???2??????????????????????????????????

Saurolophus_osborni 010000100011100????0001110002100202101????000010000000001??1?112????0?0??0010201100100?10100201000111?001?202001000?00?10010000000011?10012100000000010100000000210011000100010012111111001010100000210001001010100?????0000001100010100??????210011??2?0110100000000?0?00001?212001?0?0??00001?0????1???0000010101101000?11?1?011?10??21000000010??1????1112110011?1???000100000100000000010???0???00???????????????011?1?11?21020??0103?????????????????????00?010?1?0021000000?1????1??????????????0111?0?1?0??0??01000011??102?010?????01?1?200100??3???00???????11?101001211000001000?20?????1100??021101000?00?101011021?0??0??????????1?1101?0011111110?0111?0010010?????????00??????????0??????????11??????01020??0?000020??1?000001011201??0121001110101011200?2110110011010?0002201100????2111??10?1?????????????????122?100101??1????0??1????11??????0????????1???1?112??????000???????????0????1?????????000001????0?110?00?011??2???100000?000?00?000?0????????????

Lambeosaurus_lambei 000000100011200????00011100021002121[0 1]1????000010000?00001??1?112????0?0??0010201100100?10000201000111?001?2?2001000?01?10011000000011?1001??0000000001010000000?21001100010001001111110000101010000???0001001010?00?????000000[0 1]100010100??????2????1?????1101??0?000??????00????1001???0??0??011????110?1?00001010110100001100?0110100031011000010?111??11112110011?1??00001000001000000000100000???00???????????????[0 1]11?1111121220010103112211??1210?00??????00?010?100021000000?11???1?????????20???01?1?0?1?00001001000??1001020010??110111??200100??311[1 2]10??1?????1?10??01210000001000??0?????1?00??021101000000?10101102110??0???????????11111?001111111??11?0?0010010?4???????00?????0???0001?????1001112131001020?00?000010??1?000001001201??012100?110101111200?211011002101010002200?100000211??21001210??0???10???0??1221100?01??1??1?01011?0?111100??01110?21???001?112????0?00011?11100???01??011????????000001??????11??00?011??2???10000??000?00?000?0????????????

;

ccode + 12 63 99 160 177 197 202 203 238 258 261 331 375 410 414 427 439 446 452 462 481 500 502 503 506 513 528 536 537 539 558 560 586 588 608 612 621 622 642 678 686 688 708 724 742 744 752 762 785 795 833 838 849 866 909;

hold 200000;

proc/;
