# Supplementary material for: Exploring the diversity and disparity of rhabdodontomorph ornithopods from the Late Cretaceous European archipelago
Source: Sci Rep. 2025 Apr 30;15:15209. doi: 10.1038/s41598-025-98083-z (PMC12044058; doi:10.1038/s41598-025-98083-z)

**Supplementary Information VII for:**

**Exploring the diversity and disparity of rhabdodontomorph ornithopods from the Late Cretaceous European archipelago**

Łukasz Czepiński and Daniel Madzia

**Figure S1.** Parsimony analysis using equal weights. Strict consensus tree. Numbers on nodes show Bremer support values.


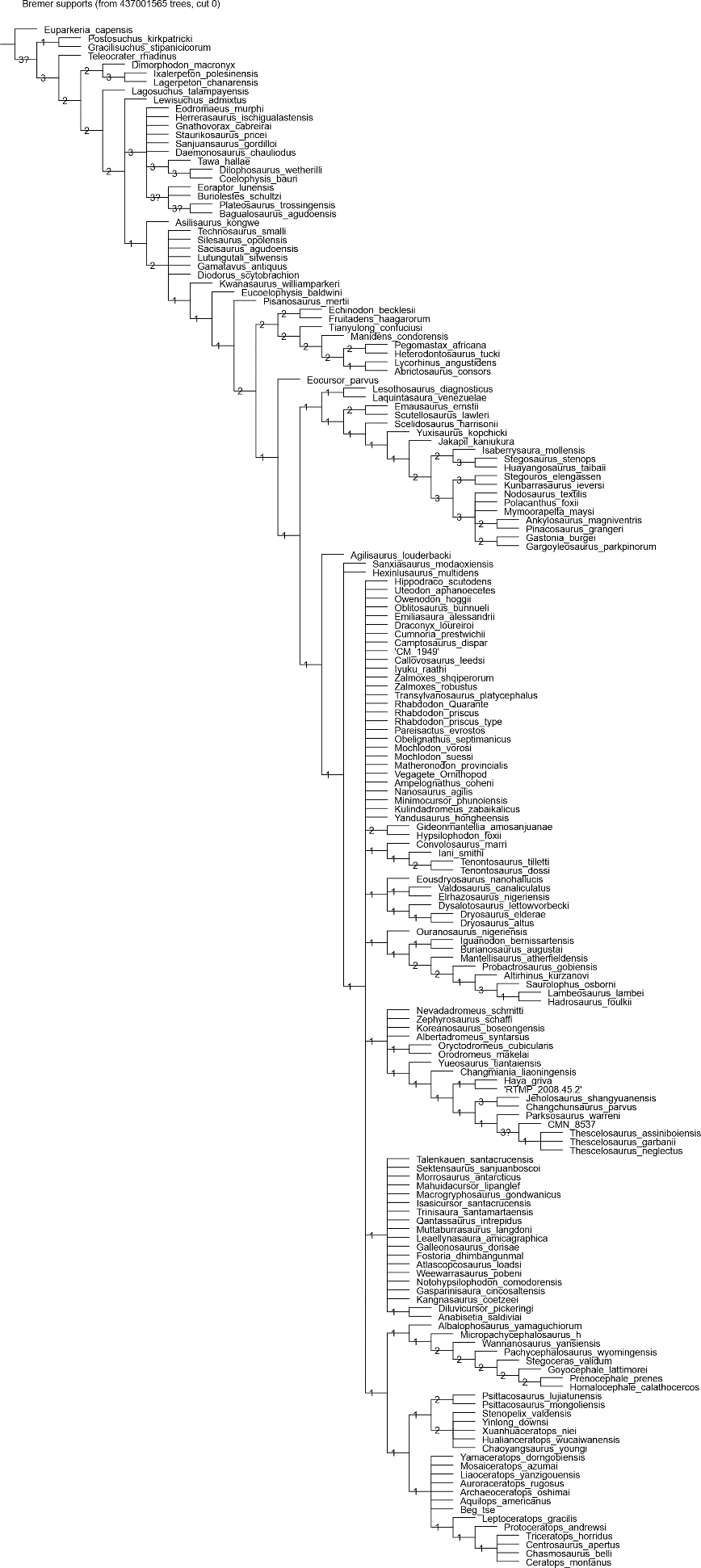


**Figure S2.** Parsimony analysis using equal weights. Majority rule consensus tree. Numbers on nodes show the percentage of the most parsimonious trees that found the nodes.


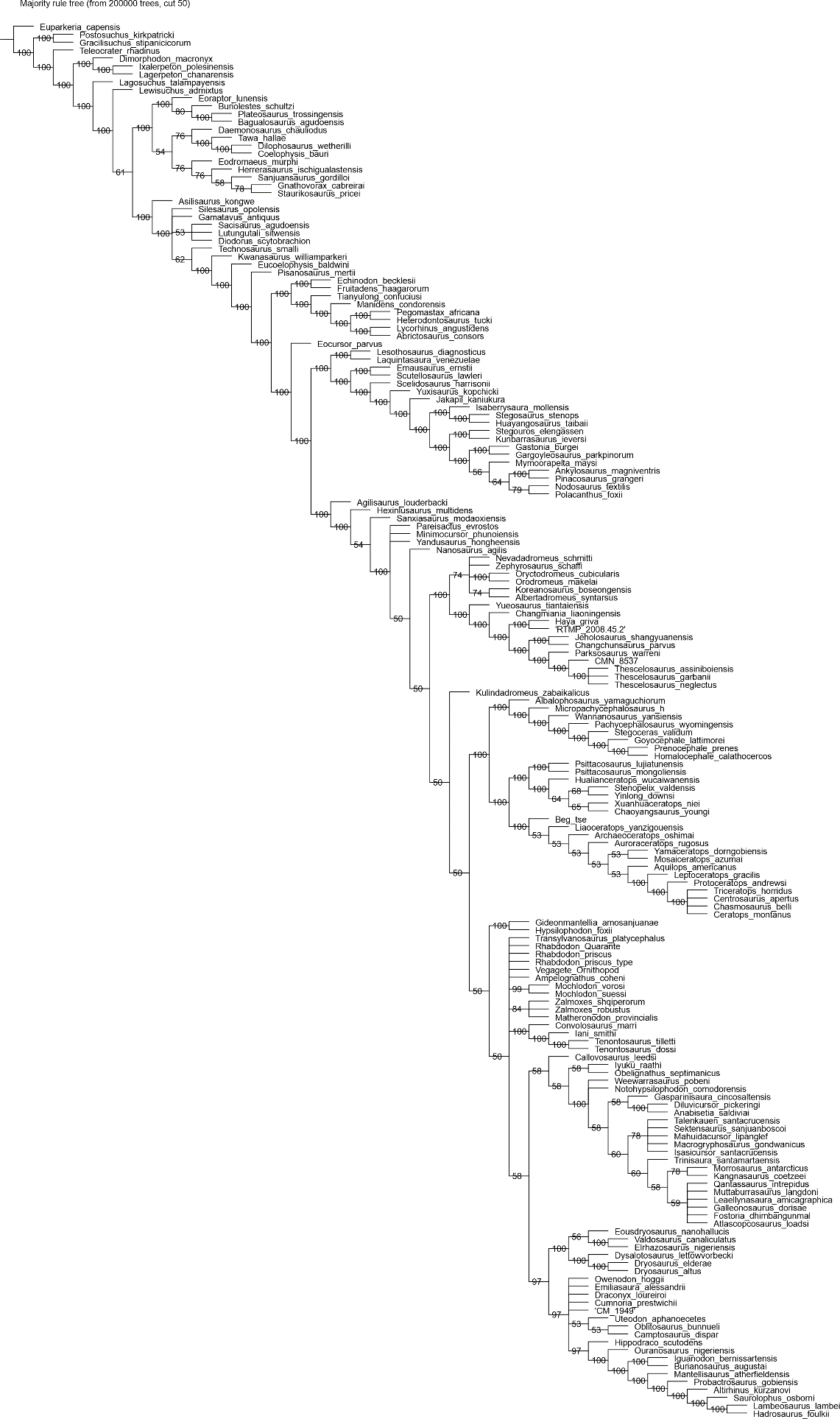


**Figure S3.** Parsimony analysis with implied weighting (*K* = 12). Strict consensus tree.


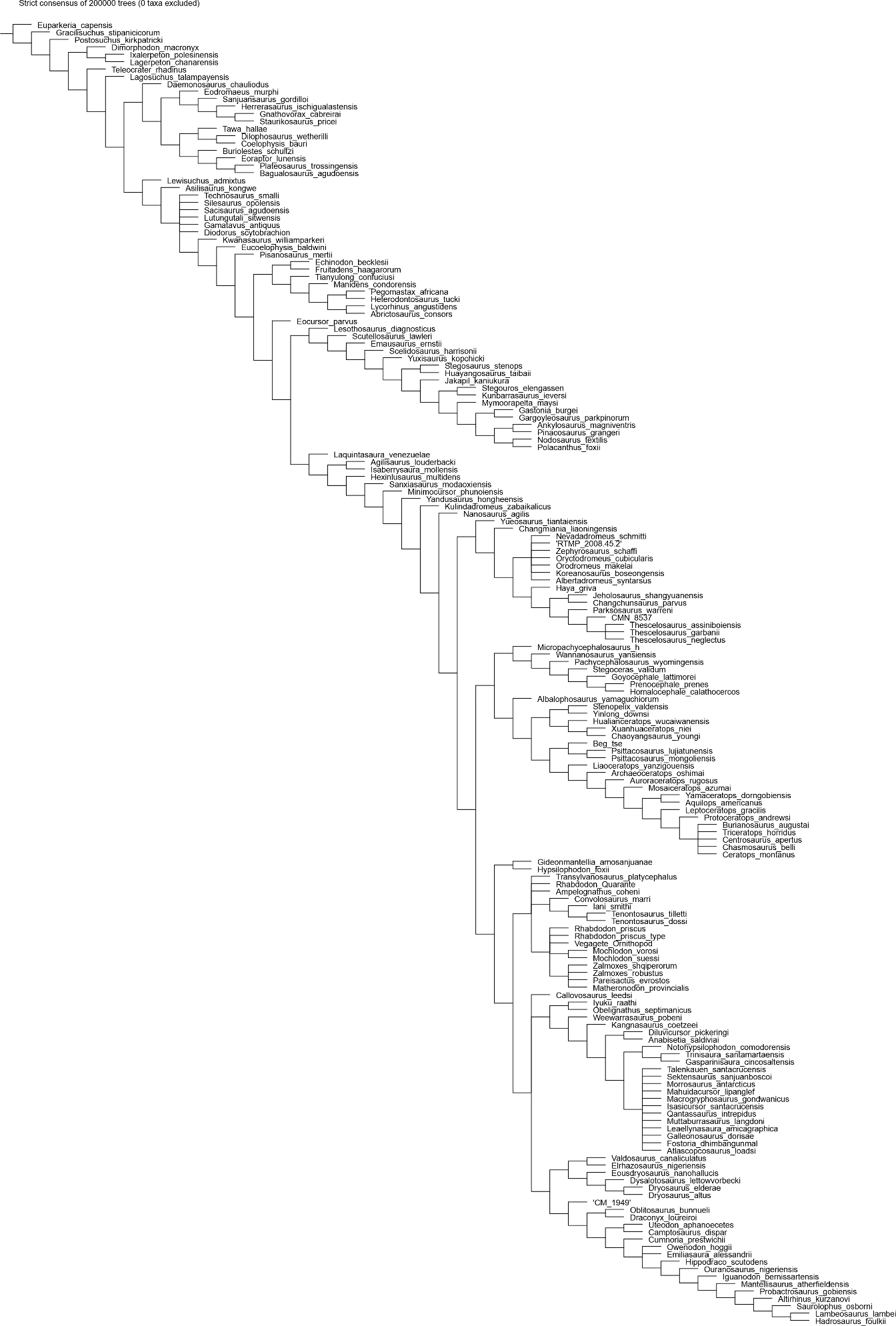


**Figure S4.** Parsimony analysis with implied weighting (*K* = 12). Symmetric Resampling.


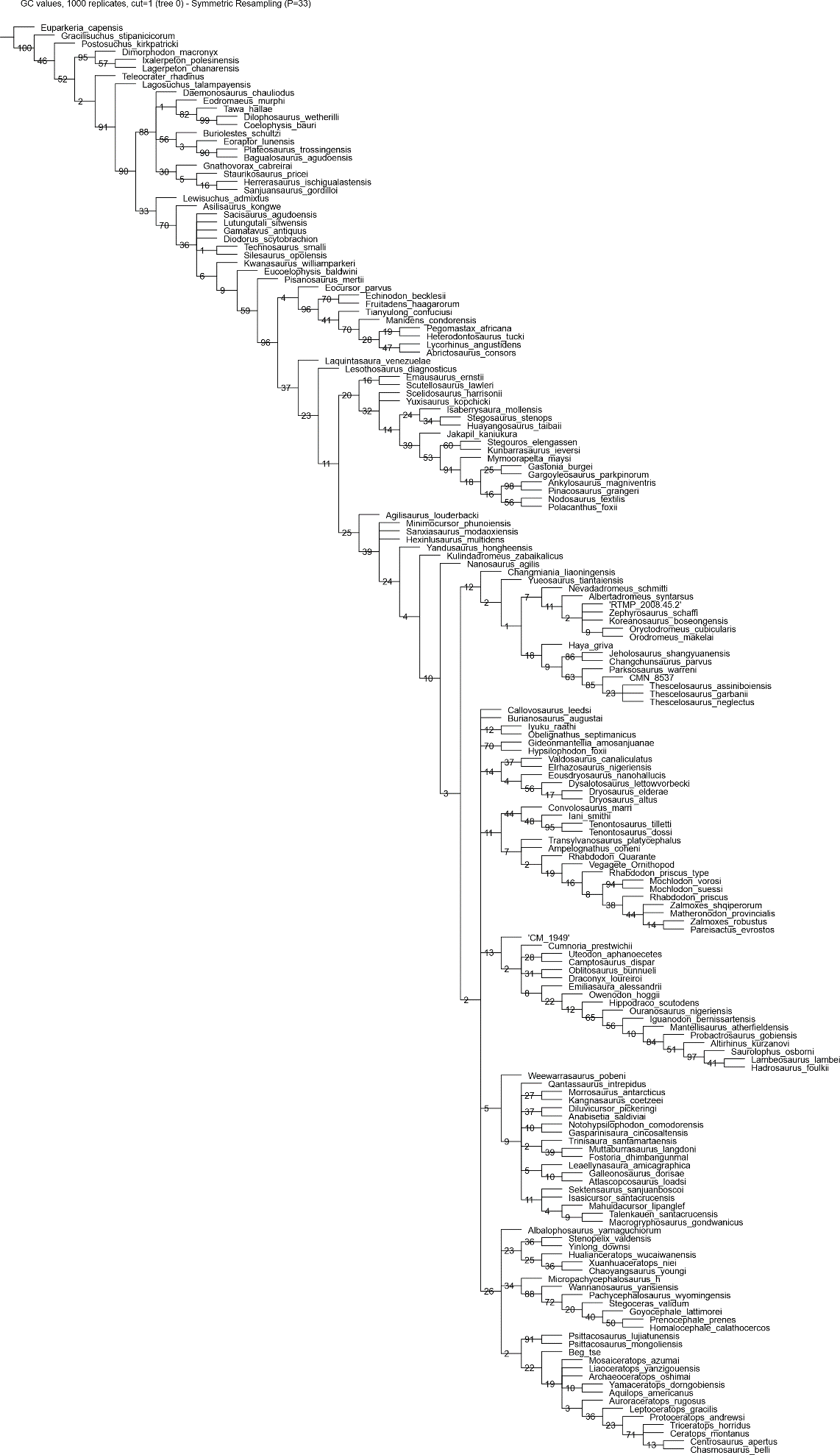


**Figure S5.** Parsimony analysis with implied weighting (*K* = 15). Strict consensus tree.


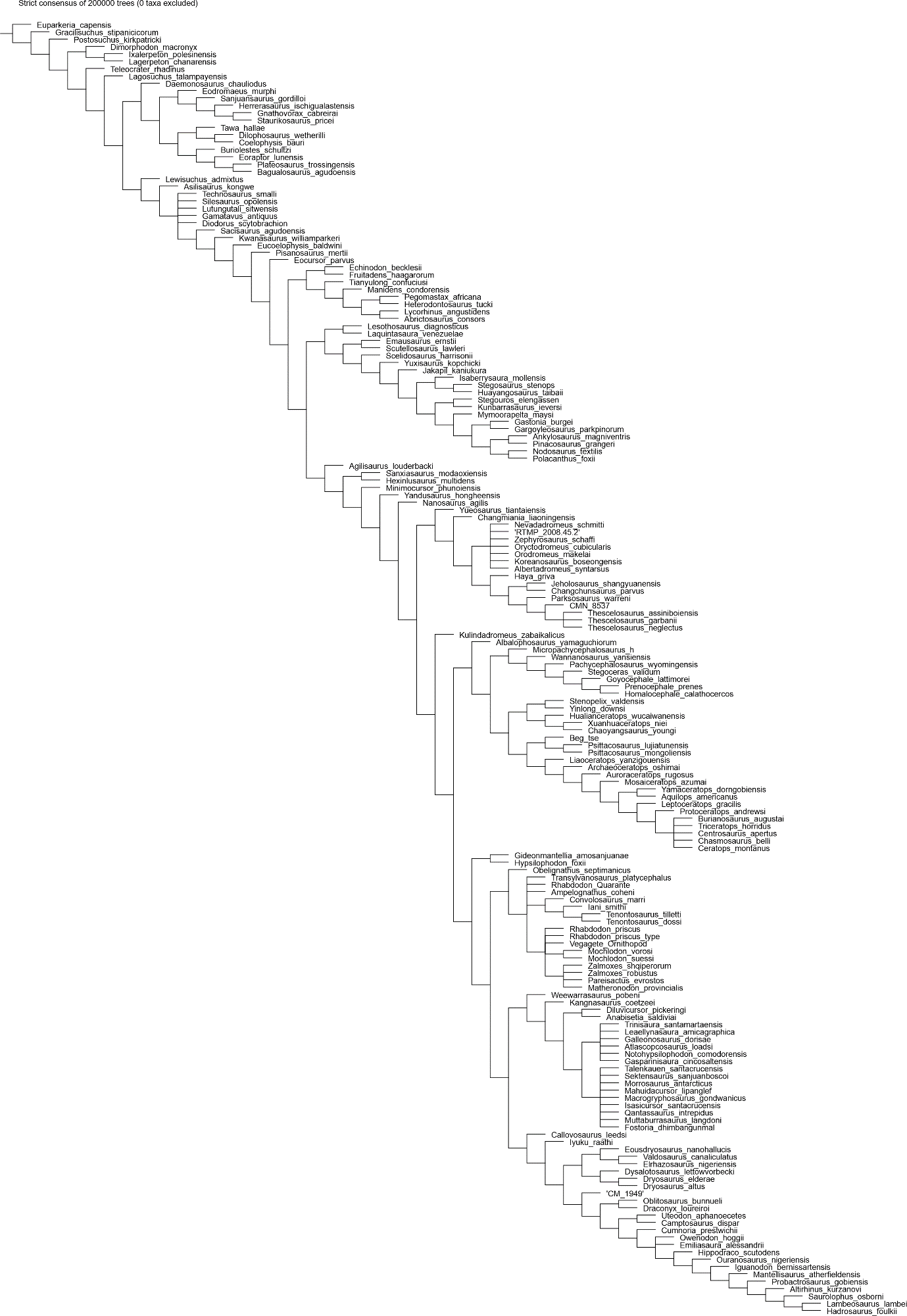


**Figure S6.** Parsimony analysis with implied weighting (*K* = 15). Symmetric Resampling.


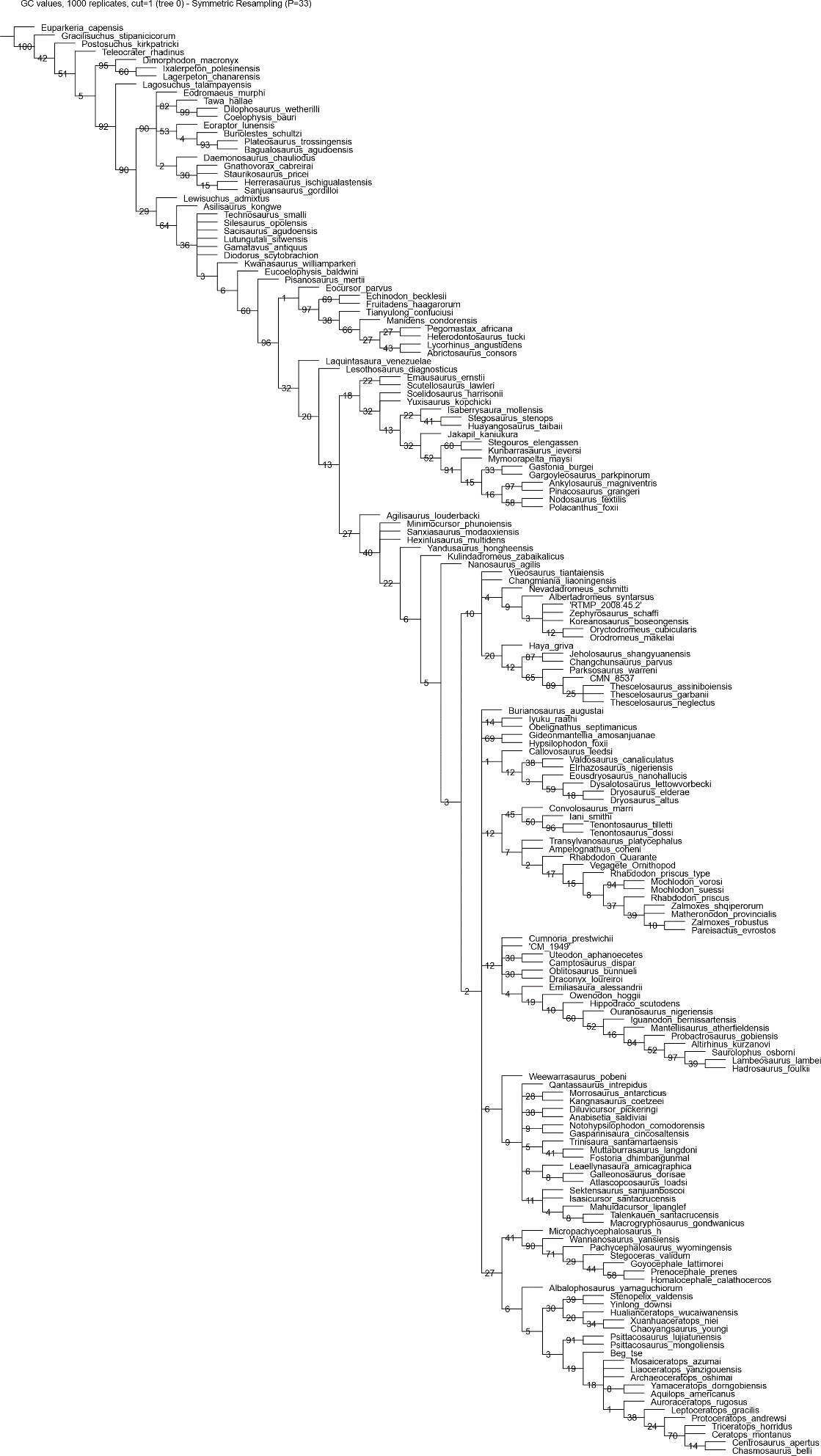


**Figure S7.** Parsimony analysis with implied weighting (*K* = 21). Strict consensus tree.


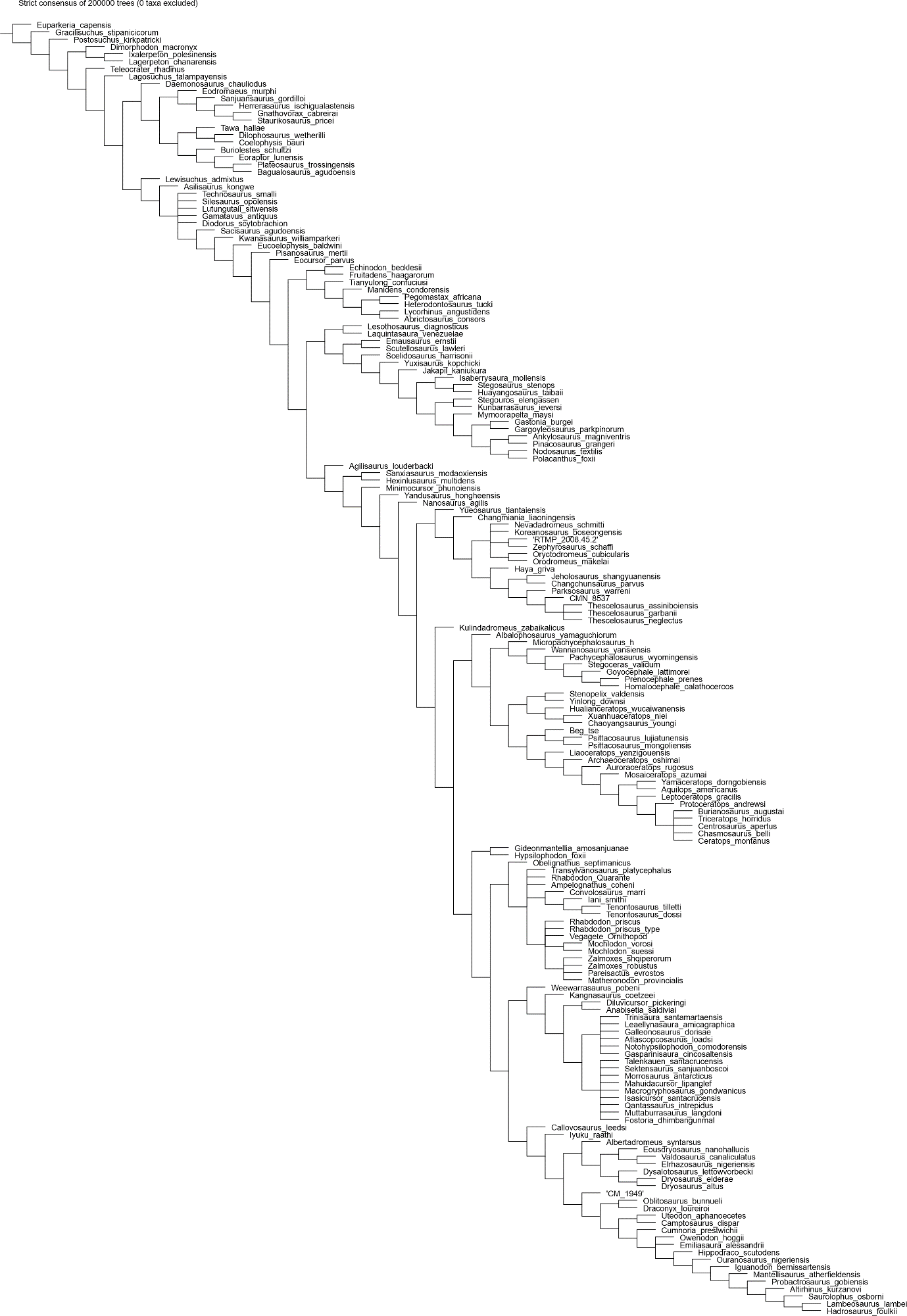


**Figure S8.** Parsimony analysis with implied weighting (*K* = 21). Symmetric Resampling.


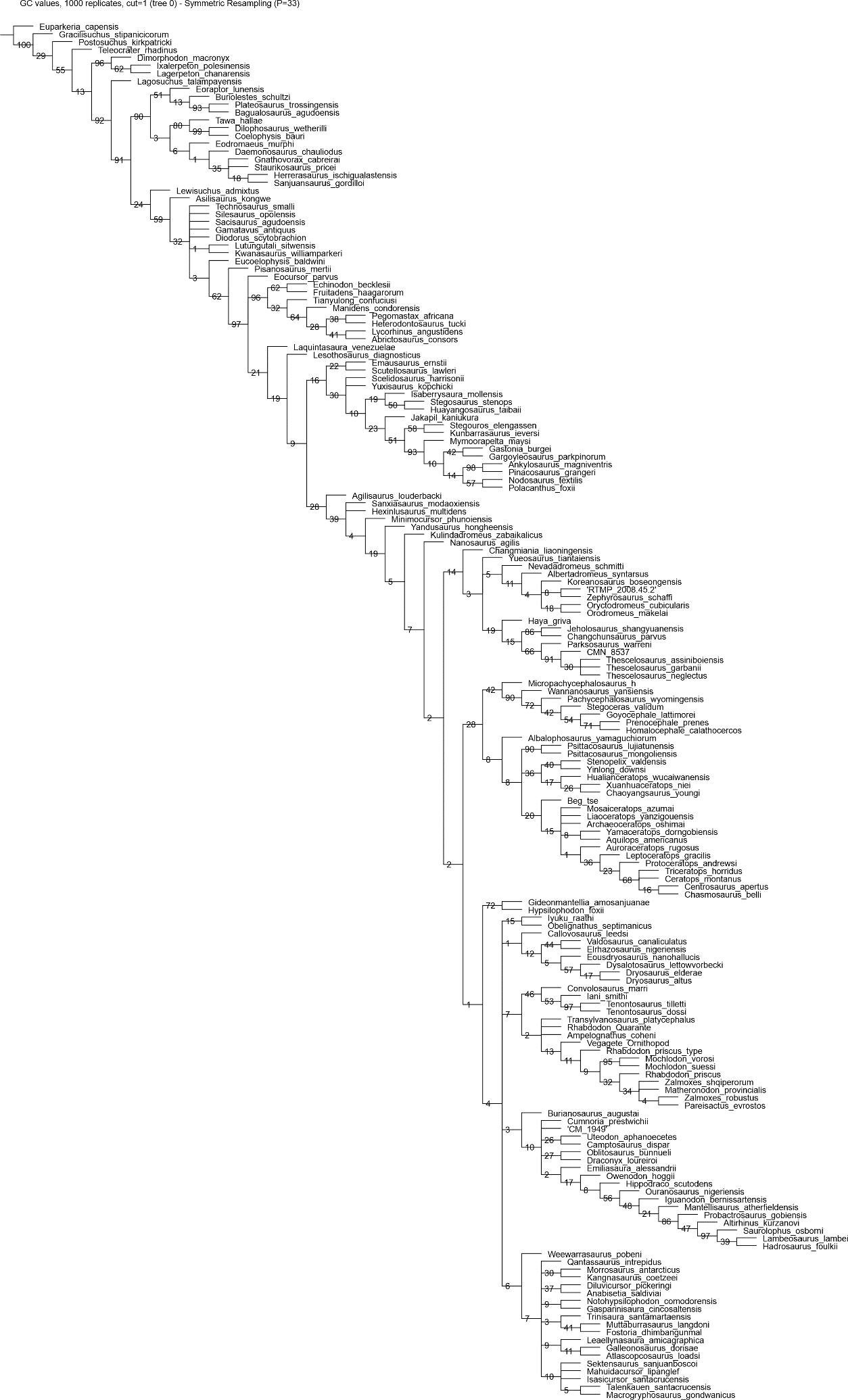

Supplement: Supplementary file 7 — Supplementary Material 7 [file 41598_2025_98083_MOESM7_ESM.docx]
